# Pursuit Eye-Movements in Curve Driving Differentiate between Future Path and Tangent Point Models

**DOI:** 10.1371/journal.pone.0068326

**Published:** 2013-07-22

**Authors:** Otto Lappi, Jami Pekkanen, Teemu H. Itkonen

**Affiliations:** 1 Cognitive Science / Traffic Research Unit, Institute of Behavioural Sciences, University of Helsinki, Helsinki, Finland; 2 Cognitive Science, Institute of Behavioural Sciences, University of Helsinki, Helsinki, Finland; University of Muenster, Germany

## Abstract

For nearly 20 years, looking at the tangent point on the road edge has been prominent in models of visual orientation in curve driving. It is the most common interpretation of the commonly observed pattern of car drivers looking through a bend, or at the apex of the curve. Indeed, in the visual science literature, visual orientation towards the inside of a bend has become known as “tangent point orientation”. Yet, it remains to be empirically established whether it is the tangent point the drivers are looking at, or whether some other reference point on the road surface, or several reference points, are being targeted in addition to, or instead of, the tangent point. Recently discovered optokinetic pursuit eye-movements during curve driving can provide complementary evidence over and above traditional gaze-position measures. This paper presents the first detailed quantitative analysis of pursuit eye movements elicited by curvilinear optic flow in real driving. The data implicates *the far zone beyond the tangent point* as an important gaze target area during steady-state cornering. This is in line with the future path steering models, but difficult to reconcile with any pure tangent point steering model. We conclude that the tangent point steering models do not provide a general explanation of eye movement and steering during a curve driving sequence and cannot be considered uncritically as the default interpretation when the gaze position distribution is observed to be situated in the region of the curve apex.

## Introduction

Car driving is one of the most studied forms of visually guided self-motion in a real physical environment, and driving through bends one of the most extensively researched and modeled forms of human locomotion in naturalistic settings. The central questions are what visual cues drivers use to control their speed and heading. Many studies have examined the gaze position in the road scene during real and simulated driving tasks. Two main classes of steering models have been proposed. The first class of models posits that drivers use the tangent point (TP) on the road edge [1–3]). The second class postulates that the gaze targets are points on the future path (FP), parts of the road surface the driver intends to pass over [4–7].

For nearly 20 years, *looking at the tangent point* has been the most prominent approach in models of visual orientation in curve driving, and the most common interpretation of the frequently observed behavior that car drivers orient visually towards the apex of the curve. Indeed, in the visual science literature, this behavior has come to be called *tangent point orientation* – even though it remains to be empirically established whether drivers are actually looking towards the inside of the bend because of the tangent point, or because they are targeting some point on the future path close to it.

**Figure 1:**
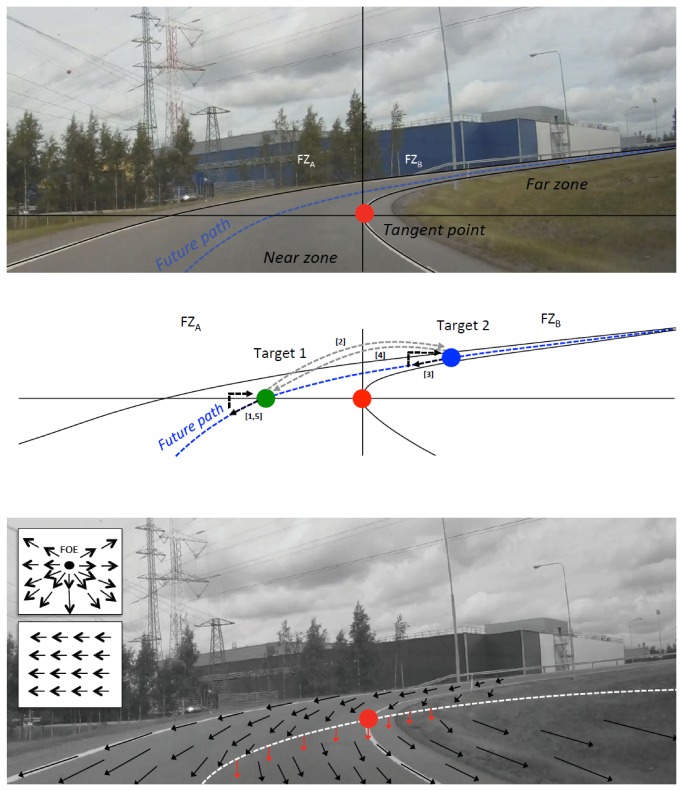
Near zone, far zone, tangent point (top), and the concept of optical flow lines (bottom). *Top*. The tangent point (TP), the near zone (NZ), and the far zone (FZ) beyond the tangent point on a motorway on-ramp. Dotted blue line: future path. Here, we define the near zone as the visible road surface and edges up to the tangent point level and the far zone as road surface and road edges further away than the tangent point. We further divide the far zone into the far zone *adjacent* to the tangent point (FZ_a_, the part of road surface visible in the top left quadrant), and the far zone *beyond* the tangent point (FZ_b_, the part of road surface in the top right quadrant). *Middle*. Potential target points on the future path. Target 1 (green): future path reference point of Boer’s model [3]. If the location at this point is fixated and tracked, this would create OKN to the left of TP (fixation Target 1, smooth pursuit of the corresponding point on the road, re-setting saccade to Target 1). Target 2 (blue): a hypothetical target point in the far zone beyond the tangent point. Targeting this point in the far zone beyond TP would create OKN above and to the right of TP. Gaze polling ( [18], see text for further explanation) could take the following form: (1) OKN around Target 1, (2) a polling (out) saccade to Target 2, (3) OKN around Target 2, (4) a polling (in) saccade to Target 1, (5) OKN around Target 1. *Bottom*. Schematic illustration of optical flow in the road scene. Compared to the simple radial optic flow emanating from a focus of expansion (FOE) during linear translation in the direction of the visual axis (inset, top), or the homogenous horizontal optic flow during observer rotation at a stationary point of observation (inset, bottom) the optic flow pattern during curvilinear motion is rather complex. The main picture gives a schematic illustration of the flow pattern (for geometric analysis see 3). The tangent point falls on an imaginary circle (dotted white curve) from the current vantage point through the curve center, and inversion of the horizontal component of optical flow in the flow field occurs at this curve (at which optical flow is vertical). In the far zone adjacent to and beyond the tangent point, optic flow has a horizontal component opposite to the direction of the curve (to the left in right hand bends), and down; below the curve, below the tangent point, the flow has a horizontal component in the direction of the curve. Note that the TP is not a fixed physical point in the scene, and hence does not follow the local direction of flow – the physical point on the road edge corresponding to the tangent point travels forward as the vehicle moves into the bend.

It has proven difficult to test these alternatives (TP vs. FP) quantitatively in real (or even simulated) driving. The main reason is probably that it is quite challenging to differentiate between the hypotheses by area of interest (AOI) methods that rely on quantifying the relative frequency of gaze landings in an AOI centered on a putative target point. This is because with realistic AOI sizes and typical curve geometry, the AOIs frequently overlap: when the future path traverses within a few degrees of the tangent point (compare Figure 1), tangent point orientation and future path orientation are essentially equivalent for AOI methods.

The point of departure for the present paper is the idea that the different hypotheses of visual orientation during curve driving (tangent point vs. future path) can be related to *different eye-movement patterns* as well as different gaze target locations. Specifically, we take advantage of the properties of optical flow and optokinetic pursuit movements which have been recently demonstrated to occur during real [8] and simulated [9] curve driving.

These are slow eye movements (SEMs) that follow a moving target object or track a location moving in relation to the observer (*smooth pursuit*) and/or local optic flow around the point of regard (*optokinetic reflex*). They are usually followed by a saccadic eye movement that re-sets gaze to target, creating a repeating pattern known as optokinetic nystagmus (OKN) of altenating slow phase (SP) pursuit movements and quick phase (QP) saccadic movements. OKN is generally considered to be a compensating mechanism that maintains image stability during global flow in the visual scene caused by self-motion through a textured environment. Optokinetic reflex (OKR) is typically regarded as an automatic process, driven by retinal slip of the image. *Smoothpursuit* of a moving a target, on the other hand, is considered a more complex process involving top-down predictive control. The underlying visual mechanism responsible for eliciting these eye-movements during the driving task have not been determined, so we have opted for the neutral term optokinetic pursuit eye-movements (“optokinetic pursuit”, or “pursuit” for short) to designate the pattern of eye-movements, without taking a standpoint regarding the underlying neural mechanisms.

### Tangent point models and future path models

The tangent point is the point in the driver’s visual field where the apparent visual orientation of the inside lane edge or road shoulder is reversed (see Figure 1, top). We use the term *tangent point orientation* for the behavior displayed by car drivers that they orient visually towards the apex of the curve (the inside kerb or line edge of a bend, in the general region of the tangent point). This highly reproducible naturalistic visual behavior has been extensively studied theoretically [1–4,6,7,10], as well as empirically both in the field [11–14] and in simulators [15–17]). As of yet, however, there exists no universally accepted functional *explanation* for this behavior. Two main classes of models have been proposed.

We call *tangent point models* all steering models that explain tangent point orientation by postulating that

1. tangent point orientation is mainly a result of *tracking* the tangent point (rather than contiguous points on the future path).
2. the tangent point is tracked *because* it provides preview information of road geometry relevant to adjusting steering

Tracking the tangent point, in turn, means that the tangent point is the gaze target of foveal vision – that the driver is “fixating” (or trying to fixate) the TP. Various models based on these assumptions (providing different theoretical accounts of the relevant information obtained from the TP) have been put forward by Land and Lee [1], Land and Wann [3], and Authié and Mestre [3].

Other models in the literature do not assume that the driver is targeting the tangent point. Instead, they are based on the use of a target point or reference point on the *future path* – points on the road surface the driver wishes his locomotor trajectory to fall on. Steering models based on targeting points on the future path have been put forward by Boer [4], Kim and Turvey [5], Wann and Swapp [6], and Wann and Wilkie [7,18].

*Future path models* posit that:

1. a target point on the future path is tracked because it provides preview information of road geometry relevant to adjusting steering
2. tangent point orientation is mainly a result of contiguity of the future path reference point(s) and the tangent point.

The tangent point models and future path models predict similar qualitative behavior: orientation in the general region of the tangent point, but for different reasons. In the future path models this is a result of the spatial contiguity of the future path and the tangent point in the driver’s visual field, not active “fixation” of the tangent point.

### The models’ predictions of gaze behavior

In their seminal study, Land and Lee [1] found that 0–1 s after turn-in the drivers’ gaze was within three degrees of the tangent point over 75% of the time, and that the distribution of fixations in the curves was "centered within a degree of the tangent point"; i.e. the area of highest fixation density was within 1 degree of the tangent point. Follow-up studies conducted by Underwood [11] and Kandil [13,14] showed that gaze dwell time in a 2 degree radius AOI could vary from 10–15% (Underwood) to 50–60% (Kandil). Differences might be due to individual differences, differences in road geometry, and/or methodological differences in defining operationally what counts as “fixating the tangent point”. In any case, if the tangent point is fixated we would expect to find the modal orientation of the visual axis to be the same for all individuals (when referenced to the tangent point), and a substantial portion of gaze to be concentrated in a fairly narrow region (a few degrees across) around modal position at the tangent point.

Unlike the tangent point, the future path presents no singular geometrical reference point that would act as a target point for the driver (or center of an AOI for the researcher). A reference point must be chosen by some criteria.

In Boer’s [4] model a reference point on the desired future path “next to the tangent point but slightly into the road” is chosen as a target point for steering and fixation (See Figure 1, middle). This choice is not derivable from the requirements of the steering model as such: any point on the future path point far enough to provide a meaningful constraint for planning trajectory curvature, i.e. any future path point visible in the far zone, would do. Boer’s particular choice of reference point is motivated empirically, by the Land & Lee results [1].

The active gaze strategy proposed by Wann & Swapp [6] (see also 18) was originally developed in the context of steering a slalom course. There, gates provide clear physical reference points and in that particular context, Wilkie et al. [18] observed that usually the next gate n is targeted, until about 1.5 s before entering the gate when a gaze shift to the gate *n*+1 is made, but occasionally gaze polling would occur where an out saccade to gate *n*+1 would be made, followed by a return saccade to gate n. The essential *general* idea is that gaze is primarily directed to the most immediate target point, interspersed with rapid fixations of future targets further ahead, when necessary, and can be applied to locomotion in the road environment. This *gaze polling* strategy would predict that drivers gaze sample stationary points on the road, tracking each for a while and *shifting* to a new point, but occasionally *polling* points further up the road (with an *out* saccade) and then returning (with an *in* saccade).

Note that we use the term “gaze polling” for the directly observable *behavioral* pattern described in [6,7,18]. To be specific, by gaze polling we refer to eye-movements tracking (often the term *gaze sampling* is used) in sequence multiple fixed target points on the road (leading to an OKN pattern with occasional polling saccades to another target point further ahead, and back, see below). Because this is a description of a behavioral pattern it should be kept distinct from the retinal flow hypothesis ( [5,6]). Retinal flow is the pattern of optic flow on the physical projection surface of the retina, not projection of the full optic flow field to an imaginary surface perpendicular to the locomotor axis. This concept must not be confused with the concept of optic flow discussed in this paper, and the tangent point model of Authié & Mestre [3]. To make matters even more confusing, Kandil et al. [13] name the visual strategy the participants are instructed to use in one of their experimental conditions “gaze sampling”. This, however, does not refer to the kind of OKN on the future path considered in the present paper. We feel that behavior is sufficiently different from the postulates of the future path models [4,6] to warrant a different name; hence, we make a distinction between “gaze polling” and “gaze sampling”. (In fact, the instructed gaze *sampling* behavior is very much like the visual sweep model where the target point “next to TP” sweeps from an eccentric position in the visual field to alignment with locomotor axis as the vehicle rotates, so perhaps visual sweep would have been a more apposite title for that particular experimental condition). Importantly, future path models are quite compatible with the kind of nystagmus observed in [8,9] – and investigated in the present study – whereas the Kandil et al. [13] “gaze sampling” instruction clearly defines a very different gaze strategy even at the behavioral level, and regardless of whether or not gaze polling / future path orientation / OKN is based on a steering strategy relying on retinal flow.

In what follows, we will further interpret the future path models in terms of the distinction between *near zone* and *far zone* of Salvucci and Gray ( [10]; Figure 1, both top and middle). We say a future path steering point would be any point in the far zone from where the driver receives visual preview information for *guidance level control* [19]. In terms of vertical position, gaze would in this case be predicted to be concentrated *above* the tangent point (far zone), and in terms of horizontal position we would expect gaze to be distributed along the future path in the far zone – sometimes at a reference point *adjacent to* the tangent point but sometimes “polling” the road in the far zone *beyond* the tangent point.

### Optokinetic eye movements

**Figure 2:**
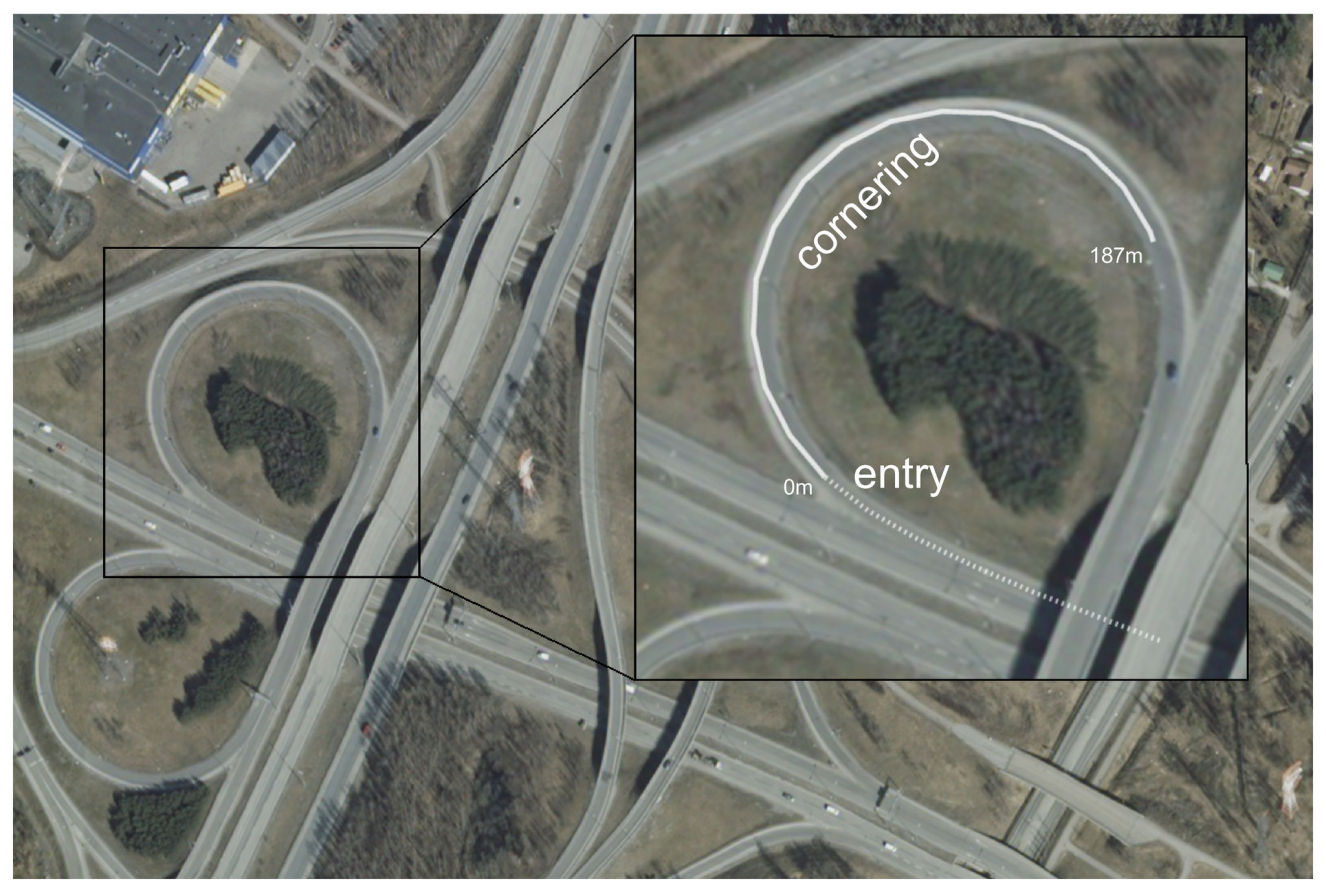
The motorway on-ramp used in the study (Kehä III - Lahdenväylä, N 60.274643; E25.086422). Inset: the entry (dotted line) and cornering (solid line) phases of the curve, based on GPS data from an individual run. For explanation of sequencing the curve see main text. The cornering phase is 187 m in length. Image source: National Land Survey of Finland open Topographic Database. license version 1.0 -1 May 2012.

If a point on the future path is being tracked (FP models), then the slow phase component of OKN represents tracking of a target location – a fixed physical point in the scene moving in the egocentric frame of reference due to self-motion of the observer – and the quick phase component represents saccade to a new target location. A clear prediction of the direction and magnitude of the pursuit eye movements can therefore be derived: since movement of the target is optic flow, SP should track the fixated point, which at the same time means following local optic flow around the point of regard. As illustrated in Figure 1 (bottom), on the future path in the far zone the optical flow has a rather large horizontal component (due to vehicle rotation) and a small vertical component (due to vehicle translation). Quantitatively, the (horizontal) rate of rotation of the eyes should be approximately one half the rate of rotation of the vehicle. (For geometric analysis that shows this, see 6, Figure 2 and the Supplement to that paper. The key idea is that the analyses in Wann & Swapp show that horizontal gaze rotation nulls the horizontal component of optic flow when the rate of rotation is -V/(2*R*), where *V* is velocity and *R* is the radius of path curvature. The result for yaw rate (ω) follows from the identity *V*/(2*R*) = ω/2). The vertical direction should be slightly downwards, reflecting the forward movement of the vehicle.

From the point of view of the models that posit fixation of the tangent point, the presence of optokinetic pursuit movements thus introduces added complications, because movement of the target (TP) is not identical to optic flow around the target point. The movement of the TP is mainly horizontal and into the curve (depending instead on the vehicle’s lateral position and longitudinal direction of motion with respect to the lane edges), while the optic flow around TP is vertical.

Unlike the future path models, TP models do not predict a specific direction and magnitude for the SP. Because the SP does not represent tracking of the target (maintaining fixation of the TP), but an extraneous influence of retinal flow via OKR, the prediction concerning the SP will depend on the specific details of how fixation and OKR are assumed to interact during tangent point orientation.

The default hypothesis would be that fixation is maintained: the flow pattern around the tangent point would not affect the rotation of the eye and these would only follow the movement of the tangent point. This could happen if the optokinetic reflex is suppressed while the tangent point is fixated.

However, that OKN is in fact reliably elicited during tangent point orientation [8,9] suggests that in so far as the drivers are “trying” to fixate the tangent point there is a powerful OKR working against maintaining “fixation” and dragging the gaze away from the fixation target (hence OKN SP) and requiring re-setting saccades to restore fixation (hence OKN QP). QP characteristics may be predicted from the assumption that the visual system is “trying” to fixate the tangent point.

Because the tangent point falls on the line of inversion (zero crossing) of the horizontal component of optic flow, the flow at the tangent point is vertical (downwards). The simplest prediction would then be that the SP pursuit movements follow the local flow at the point of regard which, with perfect TP fixation and the vertical local flow at the tangent point would means a vertical downward SP, and QP vertical upward QP.

Unfortunately, the situation may be more complicated than that, because OKR follows “global” or “regional” flow of image pattern around the point of regard, which will not perfectly coincide with the exact direction and magnitude of flow at the target point. Also, if the fixations do not land exactly at the tangent point, the local flow will not be perfectly vertical.

The re-setting QP hypothesis would thus need to be modified according to the assumed dependency of OKN SP on regional optic flow (the QP would be in opposite direction to whatever direction the SP is). Another possibility would be to launch gaze “upstream” in the flow field, so that the slow phase pursuit OKR will bring gaze back to the TP (These two interpretations were suggested by M.F. Land (personal communication) and an anonymous PLoS ONE referee, respectively. Note that for these strategies to work, the brain must be able to predict the pursuit in order to assign the appropriate landing location. The most straightforward way that this could happen is if SP follows local coherent regional flow around the point of regard).

For these reasons, SP direction and magnitude is underspecified by a hypothesis that postulates only reflexive optokinetic capture of gaze superimposed on tangent point fixation. Assumptions about the size and shape of the relevant region assumed to determine the OKN SP need to be incorporated before a definite prediction of SP direction can be derived from any TP model. While ultimately the dependency of SP on regional flow is determined by properties of the neural systems underlying OKN, at the moment such assumptions can only be derived from experiment.

When Authié and Mestre [9] demonstrated optokinetic nystagmus during tangent point oriented curve negotiation, they found that the direction of pursuit was generally not in the direction of the local flow at estimated gaze position, but systematically had a large lateral component in the direction opposite to the curve (whereas the local flow was often in the direction of the curve, indicating fixation to the inside of the kerb – gaze was typically displaced from the TP by 2–4 degrees). If the participants were in fact looking at the tangent point (or the inside of the bend), and not the contiguous areas of road surface (future path) this suggests that perhaps image stabilization was quite poor. Either that, or else the pursuit follows flow in some as-yet-unspecified flow field around the point of regard. It is physically impossible to stabilize the whole image because the flow around the fixation point is only locally coherent: there is shearing within the complex flow pattern where different elements move in different directions, compare Figure 1, middle. (In their simulator study, a bi-ocularly viewed screen image was partitioned into flow fields of different sizes – circular fields of 1 degree to 7 degrees radius, and a 49 degrees x 40 degrees rectangular field representing the whole simulator screen – but none of these resulted in a good match between OKN SP orientation and flow orientation. Instead, they found that the difference between the direction of motion of the flow field and OKN SP, a.k.a. angular bias, was quite high: over 20 degrees in 75% of cases. The lowest value was in fact obtained for the whole screen for which a coherent flow field does not exist.

We would point out that in binocular viewing, smooth pursuit follows flow of scene elements at the plane of fixation [20,21]. This would suggest that the local flow field in real 3D environment may also incorporate depth information by tracking retinal image elements with zero disparity. To our knowledge it has not been analysed theoretically or investigated experimentally whether disparity is actually used in maintaining fixation on tangent point / future path target point in a locomotor task, e.g. whether disconjugate eye convergence occurs during SP or whether the QP is executed before this becomes necessary. As a final note, the participants in that study were also instructed to drive ”as fast as possible without ever leaving the right lane” which – especially in conjunction with the lack of stereoscopic depth information – might have led to a different gaze strategy compared to normal everyday driving (cf. [22]).

Based on this analysis of how these different hypotheses of visual orientation during curve driving can be related to different *eye-movement patterns* as well as different *gaze target locations*, we can se how a more detailed understanding of optokinetic pursuit movements can be employed as a complementary methodology for differentiating between predictions of the tangent point vs. future path models. This is the task we set ourselves upon in this study. For this purpose, we develop a novel method of identifying pursuit movement in noisy on-road eye-movement data, which allows us to analyze the direction and magnitude of OKN SP’s in in real curve driving.

## Methods

### Subjects and equipment

A convenience sample of 21 subjects participated in the study (11 M, 10 F, age 22 y -46 y, mean 27 y). In the end, data from four subjects were omitted due to poor signal quality, leaving a sample of 17 participants. Participants were recruited through personal contacts and university mailing lists. All participants held a valid driver’s license, and the experience level of the participants varied from 1000 km to over 1 000 000 km self-reported lifetime kilometrage. The subjects reported no medical conditions that might affect eye movements, and had normal or corrected to normal eye-sight (The participants with corrected eyesight wore contact lenses in the experiment).

The study was covered by a written approval from the ethics committee of the Faculty of Behavioural Sciences, University of Helsinki, for the use of human subjects in real traffic conditions. Written informed consent to participate in this study was obtained from each participant. This was done, in accordance with the approval of the ethics committee, in the form of a fixed-format consent form explaining the purpose of the study, the procedure, and intended use of the data (for scientific purposes only). Paper copies of the consent forms were archived.

The instrumented car was a model year 2007 Toyota, Corolla 1.6 compact sedan with a manual transmission. The passenger side was equipped with brake pedals and extra mirrors, as well as a computer display that allowed the experimenter to monitor vehicle speed, the operation of the eye-tracker and the data-logging systems. The car was equipped with a two-camera eye tracker operating at 60 Hz (Smart Eye Pro version 5.5 www.smarteye.se), a forward looking VGA scene camera and a GPS-receiver. Vehicle telemetry (speed, steering, throttle, braking and horizontal rotational velocity, i.e. yaw-rate) were recorded from CAN-bus. Gaze position accuracy during calibration can be seen to be about 1–2 degrees; on the move, however, a more conservative estimate of 2–3 degrees accuracy is more appropriate (See Supplementary Methods in Supporting Information S1 for more details on calibration).

All signals were synchronized and time stamped on-line, and stored on a computer. All data preparation, visualization and analysis was done using custom-made scripts written in Python, and using the NumPy, SciPy and matplotlib packages. The source code of the analysis scripts and the dataset used are available at https://gitorious.org/opt-pursuit-analysis under GNU AGPLv3 and Creative Commons BY-NC-ND licenses (see the website for license details).

### Route & procedure

The measurements were carried out on an on-ramp of a suburban dual-carrageway (Figure 2), with 80 km/h posted speed limit. The ramp was chosen because of its ideal curve geometry from the point of view of the present investigation: the projection of the road surface in the visual scene allows us to better resolve the tangent point from the road surface beyond it (the elevation produces differentiation of the vertical angle of the tangent point and the road), and, also, the curve has a distinct cornering phase where the curve radius remains relatively constant for a significant period of time, as the theoretical analyses of tangent point orientation [1–3] use the idealizing assumption of (locally) constant radius. The length of the curve (the car rotates 270^o^) and relatively large radius mean that the duration of the curve – and the cornering phase in particular – is therefore sufficient to provide abundant data on eye-movement patterns during cornering (over five minutes of pure cornering phase data per subject). Here, the road geometry also allowed us most reliably to identify the tangent point algorithmically. Theoretically, we also reasoned that given the reliable observation of high concentration of fixations near the tangent point immediately before and after the turn point [1], if there are indeed other gaze strategies at work during curve driving, we would have the best opportunity to observe them by looking at a later part of the bend.

The location was 15 km from the university campus, and the participant drove the car to the site in order to familiarize him with the vehicle. The ramp was driven 16 times, and the eye cameras calibrated on arrival and after the 5th and 11th run to maintain good calibration throughout the session.

All drives were carried out in daylight, sometimes in varying weather conditions (overcast or light rain). The participants drove at their own pace, and were instructed to observe traffic laws and safety. In addition to the participant, a member of the research team (TI) acted as experimenter. He was seated on the front passenger seat, giving route directions, ensuring safety, monitoring the recording and performing the calibrations.

### Curve phase segmentation

The data was segmented into discrete curve-driving events based on GPS coordinates and vehicle telemetry. We operationally sequence the curve negotiation episode into distinct *phases*, decomposing the physical geometry of the turn (or the vehicle’s physical trajectory through it) into the following segments. The curve *entry* phase begins when the driver begins to rotate the vehicle by turning the steering wheel at her chosen turn point. Both the steering wheel angle and vehicle yaw rate increase progressively throughout the entry phase (in normal everyday driving, assuming no rear wheel skid). In very long curves (which is what the turns analyzed here are), the entry phase is followed by a steady *cornering* phase where the steering wheel angle and vehicle rate of rotation remain relatively constant. The *exit* phase of a turn begins when the driver begins to steer out of the bend (to unwind the steering lock, the yaw rate beginning to reduce having reached a local maximum). The driver can be considered to have completely exited the corner, and having completed the entire cornering sequence, when he reaches an exit point where the vehicle is no longer in yaw. Curves where the exit point is visible during approach and turn-in (before the end of the entry phase) are considered *sighted*, curves where the exit point only becomes visible during the curve (after turning in) are considered *blind*. The on-ramp is a blind curve, the exit only becoming visible towards the very end of the cornering phase.

To render trials comparable, the data was given a location-based representation. One trial, with no traffic or other “incidents” was chosen as a reference. The vehicle trajectory in an allocentric xy plane (GPS latitude and longitude coordinates) was computed by interpolating the GPS signal. This interpolated trajectory would then be used as the template of a route-location value (meters from the beginning of the leg), with which all other signals could be associated. All participants’ trials were then mapped onto this frame of reference, by first best-matching the observed GPS values to the reference trajectory, and then projecting all observations onto the interpolated distance values. The transition points of entry, cornering and exit phases were established manually, based on median yaw rate.

### Analysis of eye-movements

**Figure 3:**
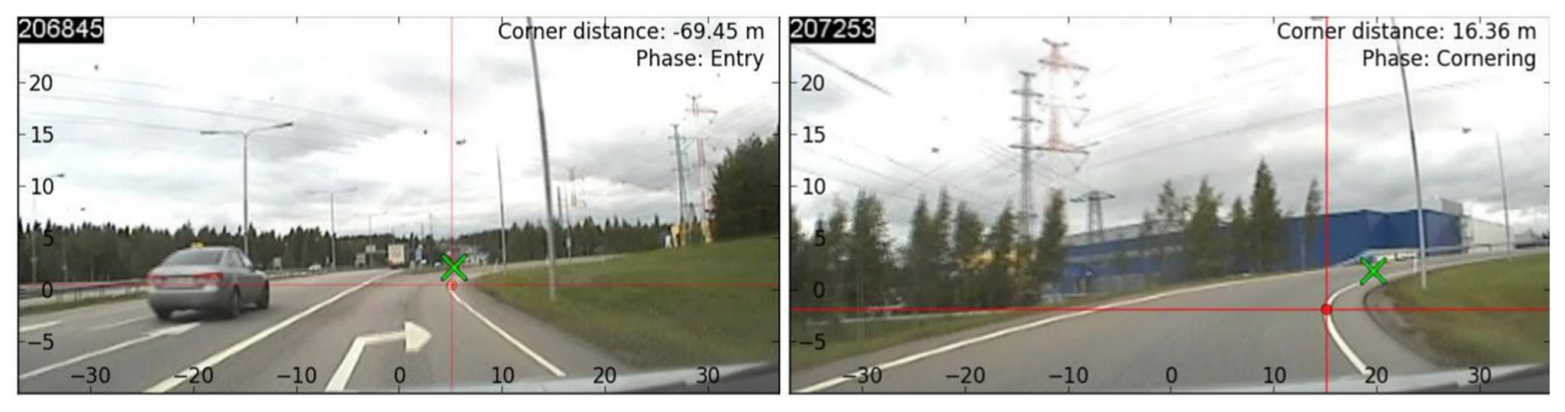
Algorithimic tangent point identification during curve entry (left) and cornering (right). The algorithm generally identifies the position of the tangent point to an accuracy of better than one degree. The green cross estimated the driver’s gaze position. Note also the lateral displacement of tangent point into the direction of the curve during entry phase (See also movie S1, S2 and S3).

To determine the presence of tangent point orientation, the tangent point was identified algorithmically from forward looking VGA video (from the SmartEye Scene camera), using an in-house developed lane detection algorithm designed specifically for finding the edgeline and tangent point in curves (Figure 3 see also movie S1-S3). This yielded time-stamped image coordinates, for each time point, which could be assigned a GPS location coordinate. The image coordinate system was physically calibrated to the angular coordinate system of the eye-tracker, so that horizontal and vertical angular displacement of gaze from the TP could be computed. After this, all gaze position data could be referenced to the vehicle frame of reference or a tangent point centered frame of reference, as required by a particular analysis.

Gaze angular velocity provides complementary information on the driver’s gaze strategy over and above gaze position. This signal also has the advantage that it is not sensitive to small measurement errors in gaze position, such as may arise from (linear) calibration bias (these afflict especially AOI based measurements, particularly when the putative targets are theoretically expected to lie within a few degrees adjacent to each other).

Ideally, a gaze velocity signal is, of course, the first time derivative of the gaze position signal, or, with discrete sampling, a time difference signal. In practice, differentiating (or taking the difference) from a noisy signal with a relatively low sampling rate is problematic. On the other hand, fixation detection algorithms suitable for on-road data with a low sampling rate rate and high noise levels (e.g. IV-T [23]), assume that during “fixation” gaze position remains stable in the egocentric (vehicle or head) frame of reference. This creates a problem when the “fixation” on the road is really a smooth pursuit (e.g. an OKN SP) with a horizontal angular velocity of considerable magnitude.

We therefore developed an optimal segmentation algorithm to partition the data into discrete eye-movement episodes (“SPs and QPs”) assuming linear eye movements, Gaussian axis-independent noise and Poisson distributed changes in the linear segments. The basis for the method is based on Optimal Partitioning described by Jackson et al. [24], with which we select the segmentation that maximizes the likelihood of the segmentation under the assumptions. However, due to outliers in the data (also assumed Poisson distributed), we expanded the method in a manner inspired by the Multiple Hypothesis Tracking method ( [25]; For details of the algorithm see the supplementary methods in Supporting Information S1). Samples categorized as outliers are left out from the local linear fit and its residuals.

**Figure 4:**
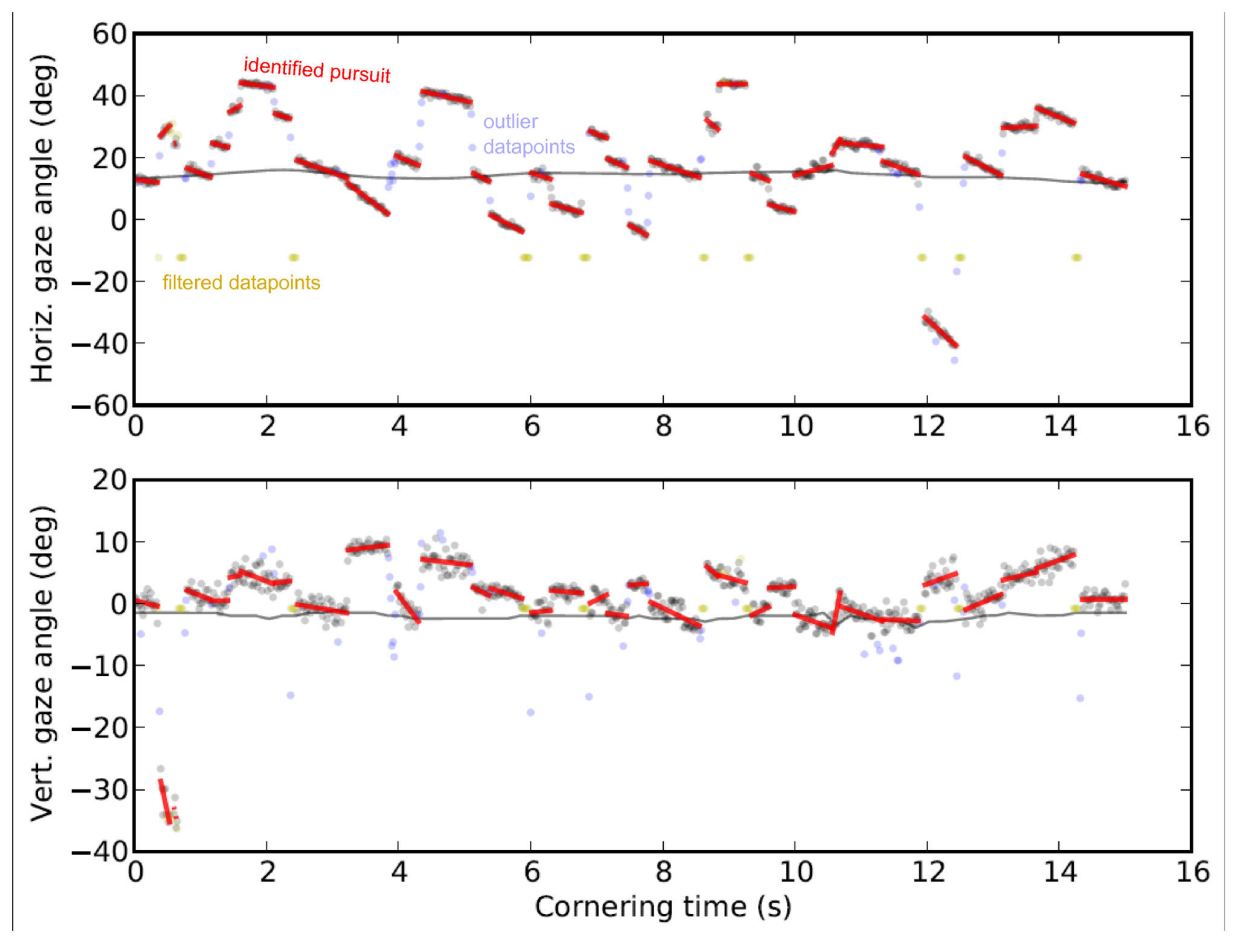
Example of raw gaze position signal and segmentation. Each segment (red) is a linear regression to the raw horizontal (*top*) and vertical (*bottom*) gaze position datapoints (gray) between the initiation and termination points of the segment, where the initiation and termination points are computed by a robust segmentation algorithm approximating a maximum likelihood linear segmentation. Blue datapoints indicate outliers not included in the regression. Yellow datapoints filtered out prior to analysis due to poor tracking/signal quality. Solid black line is the tangent point angular position as given by the lane edge detector algorithm.

The data is thus partitioned by fitting to the data points line segments representing pursuit movements. Free parameters are the expected segment length, noise and outlier frequency. The segmentation of raw data can be seen in Figure 4 where the linear segments are drawn superimposed on the raw signal.

These line segments’ slopes can then be used as an estimate of gaze velocity of each datapoint associated to the segment: non-zero slopes of different magnitudes represent smooth pursuits and saccades.

For *gaze position* we used the segmentation signal at the time coordinate of each valid gaze position observation (rather than the noisy raw gaze position observation) and *computed for each datapoint its displacement from the tangent point position giving us gaze displacement from the TP* for each point. The *gaze velocity* (horizontal and vertical components) at each point in time was likewise defined by the horizontal and vertical slopes of the linear segment associated to the datapoint.

For statistical analysis of the gaze pattern, density estimates were made of the gaze position (horizontal and vertical displacement from the tangent point) as well as change in gaze position (horizontal and vertical velocity components of gaze movement). We used a Gaussian kernel estimation, with bandwidth calculated by Silverman’s rule for the velocity density and fixed bandwidth of 1.0 for the position density. Modes were estimated from the density estimates by first finding an approximation by discretizing the density estimate and numerically optimizing the density estimate function bounded in the discretization bin.

## Results

### Driving behavior

The participants drove at a moderate pace. The average speed maintained in the cornering phase was slightly over 40 km/h (Mean = 42.7 km/h SD = 3.6 km/h, see Figure S3 and Table S1 in Supporting Information S1). During the cornering phase the vehicle yaw rate remained quite stable indicating steady-state cornering (Mean 13.8 degrees/s, SD 1.4 degrees/s, for individual data from each individual run, see Table S2 in Supporting Information S1). During curve entry, the tangent point area moves laterally (by about 15 degrees) from an initial eccentricity at the turn point of about four degrees (M = 3.8 degrees, SD = 0.9 degrees) to a stable eccentricity of 15–20 degrees (M = 17.0 degrees, SD = 1.6 degrees). The tangent point eccentricity depends on the driving line of the participant, but is quite stable during steady-state cornering and repeatable across runs.

### Gaze position and AOI results

**Figure 5:**
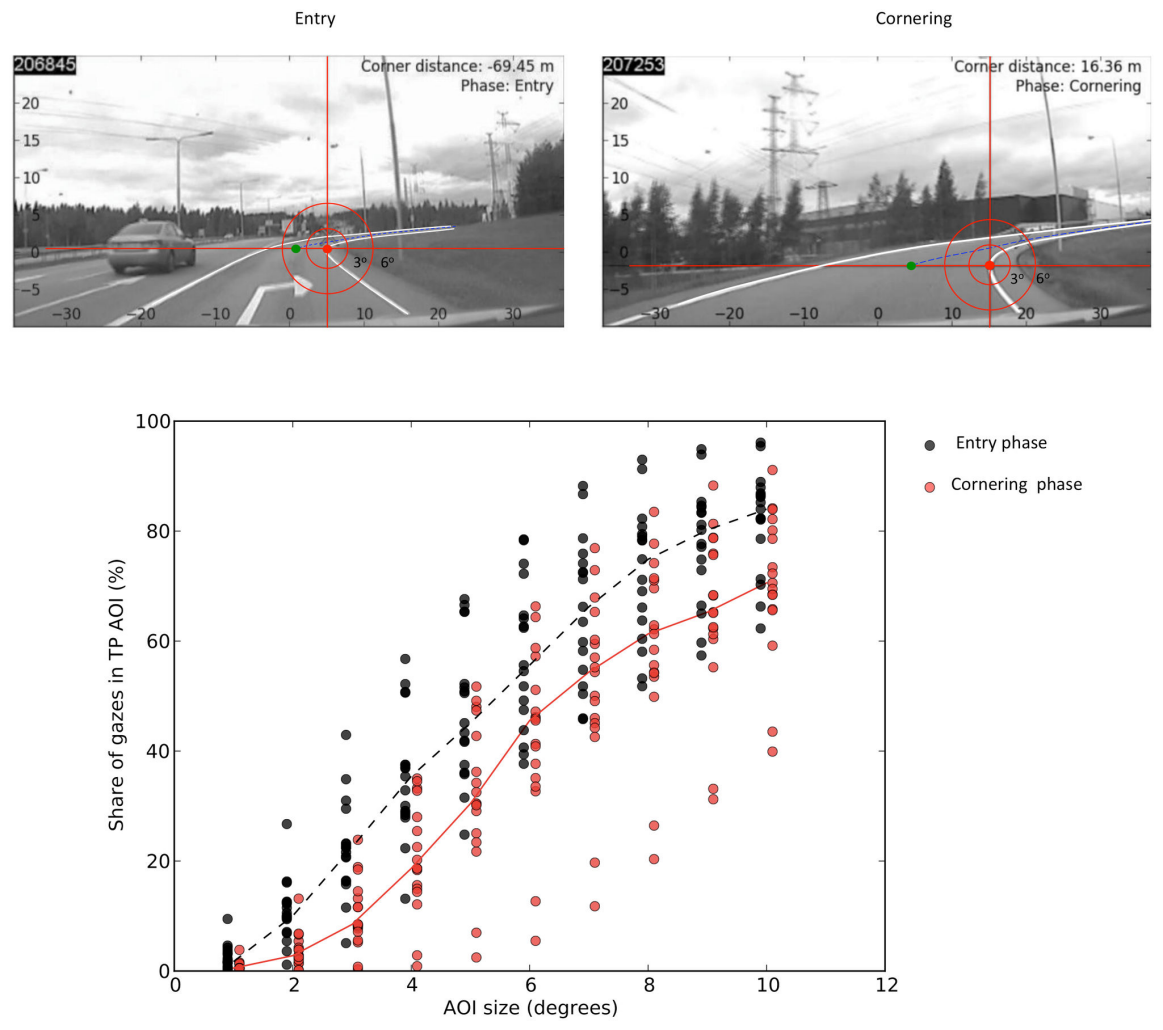
Top: 3 degree and 6 degree AOI cover much of the road surface, particularly in the entry phase. Bottom: Gaze catch percentage in different size AOI’s centered at the tangent point. Each black dot and each red dot in the top picture represents per subject median AOI catch % in the entry and cornering phases, respectively. Dotted black line (entry phase) and solid red line (cornering phase) indicate their averages, by phase. The bottom figures illustrate the problem of AOI overlap: AOIs centered on the tangent point also cover much of the future path in the far zone. Due to the projection geometry, this overlap is greater in the entry phase which may in part account for the higher gaze catch.

The proportion of gaze position observations within 2–10 degrees of the tangent point (2–10 degree radius AOI) are not dissimilar to those found in previous research reporting “tangent point orientation” (Figure 5, bottom), so the subjects may be said to be “tangent point oriented” (this is the kind of AOI catch method of presenting the data typical of the tangent point orientation literature). We see that the gaze catch percentages are higher for larger AOIs (which is logical as each smaller AOI is a proper subset of the larger one). Also, it appears that the catch percentages tend to be higher in the entry phase than the approach phase. Binomial tests for the for the sign of the variable *aggregate entry phase catch* % - *aggregate cornering phase catch* % computed for each individual show this difference to be statistically significant (*p* < 0.03) at all AOI sizes. The maximum average difference is 15.6 percentage points, in the 5 degree AOI.

It is important to note, however, that this result arises in the context of another important feature, illustrated in Figure 5 (top): large AOIs (more than 4 degrees) are completely ambiguous as to what gaze targets within the AOI are actually used, because there is considerable overlap between the TP AOI, and almost *any* AOI one might place on the future path. This is especially true during the approach and the entry, before optic flow has “opened up” the view into the curve. The apex of the bend – i.e. the tangent point region – thus appears to be an important reference direction but this, as such, is a very coarse picture of the behavior and does not tell us whether the apex region is important because of the tangent point. The AOI result as such does not tell us what gaze targets – let alone what steering strategies – the drivers might be using.

Movie S1, S2 and S3 give representative examples of three participants’ runs (chosen on basis of median gaze distance of gaze position from tangent point, i.e. in order of “tangent point orientedness”). It often appears that the gaze is not concentrated within a few degrees of the tangent point (with intermittent glances at the “scenery”). Especially once they are well set into the bend, the drivers sample the road ahead.

**Figure 6:**
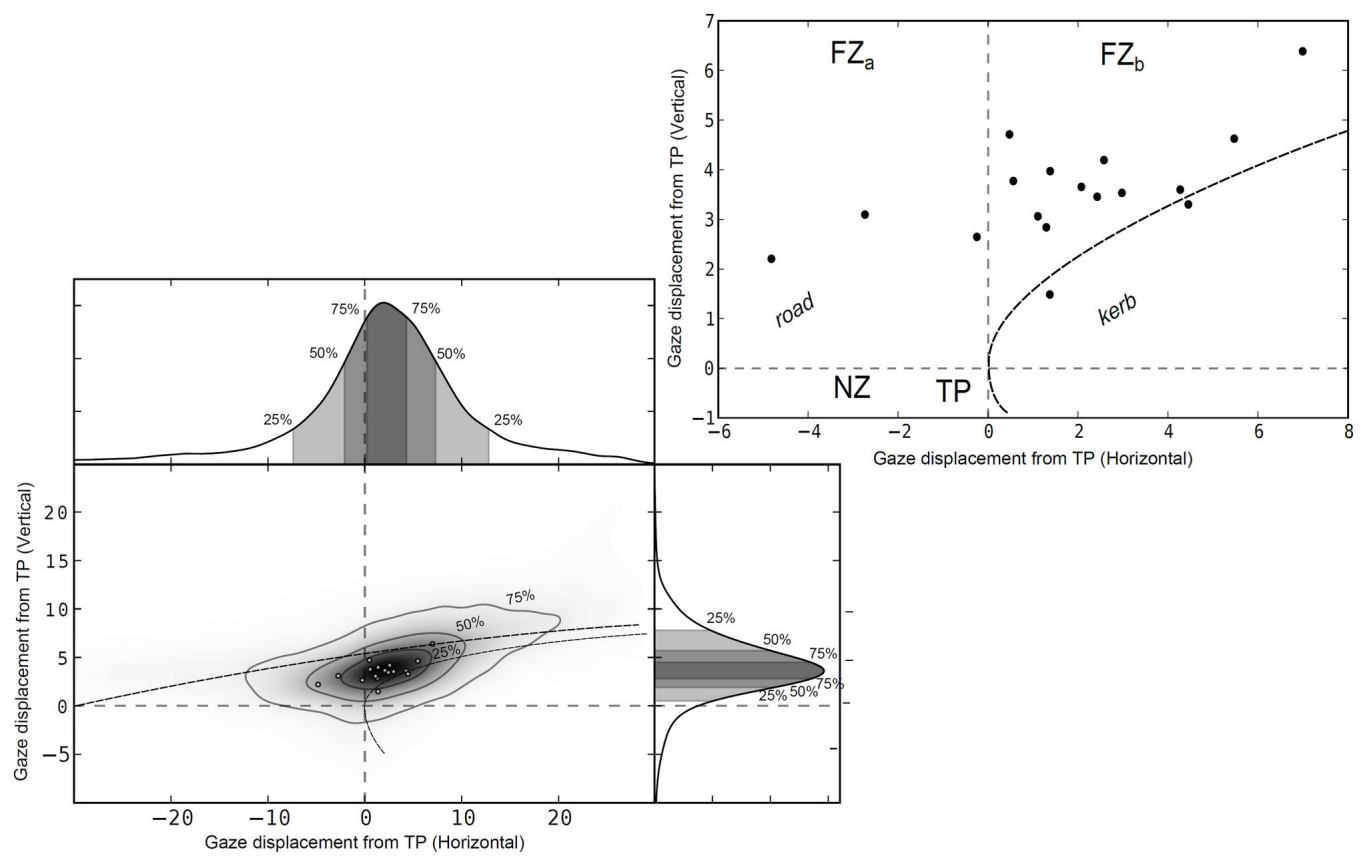
Gaze displacement from tangent point during cornering. Distribution of gaze displacement from the tangent point. Density estimate and marginal density distributions (horizontal and vertical). The tangent point lies at the origin, the near zone lies mainly in the lower left quadrant, and the far zone in the upper left quadrant (“adjacent to the tangent point”) and in the upper right quadrant (“beyond the tangent point”). Aggregate data for all subjects. The dashed contours in the main picture contain 25%, 50% and 75% of observations. Circles indicate mode of the gaze density distribution from individual subjects data. See Supplementary Results in Supporting Information S1 for individual subjects’ data.

Figure 6 displays the density distribution of *gaze displacement from the tangent point* in the data, and the modal displacement of each individual in relation to the tangent point (for individual participants’ data see Supplementary Results in Supporting Information S1). This data is gathered during the constant radius cornering phase. The figure represents some 60 minutes of data.

We see that the distribution is elongated, roughly following the visual shape of the road surface. In particular, we note that there is quite a lot of mass as far as 10 degrees to the right and to the left of the tangent point. Vertically, the mass is well above the tangent point level, especially on the right (beyond the tangent point), where the uphill bend rises above the horizon.

The typical gaze position during cornering represented by the mode does not coincide with the tangent point, and is also variable across individuals. But while there is variability in "typical" gaze position among the subjects - but consistently the overall pattern, both at the aggregate and individual level, shows gaze position to be concentrated in the far zone, above and often beyond the tangent point.

### OKN Characteristics

**Figure 7:**
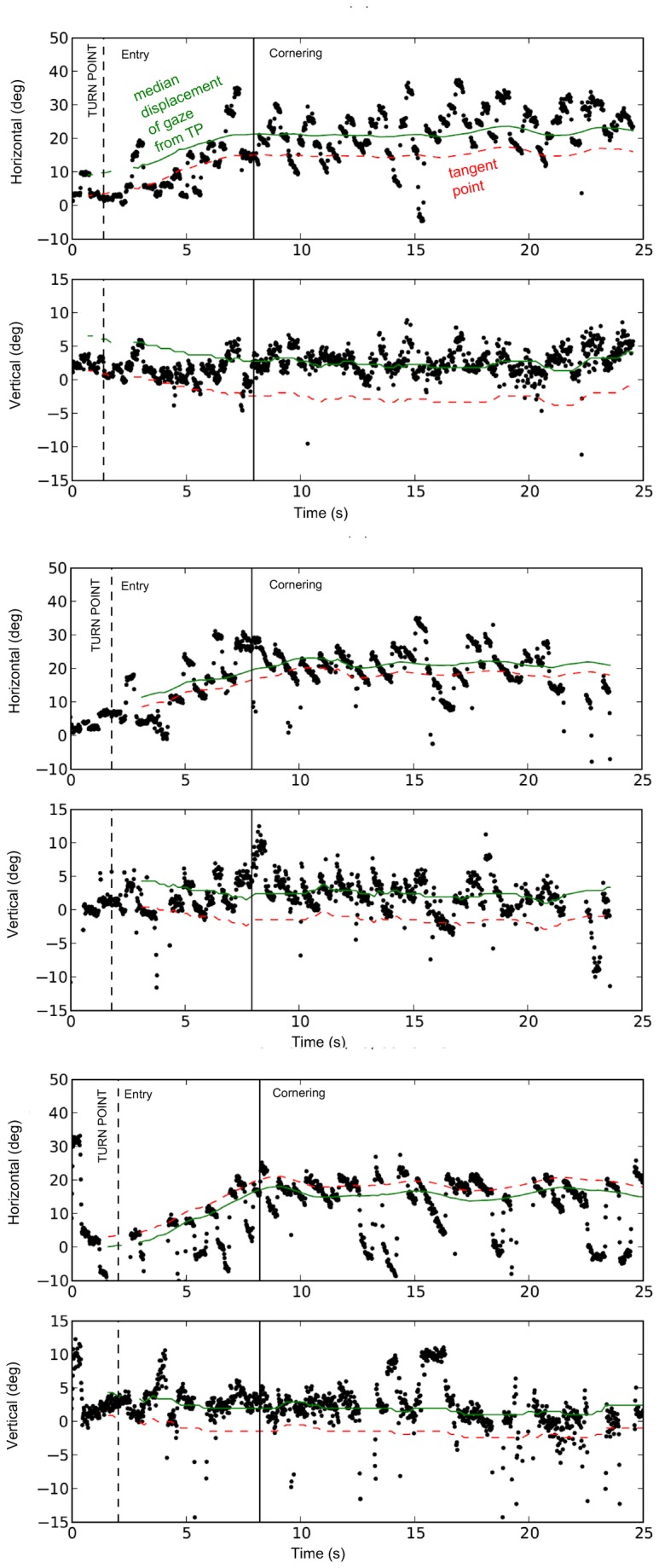
Individual trial gaze position time-series data. Horizontal gaze position in relation to vehicle centerline (degrees) plotted against time from three individual trials of representative subjects (see Supplementary Videos). Zero angle corresponds to vehicle centerline (approximately equal to instantaneous heading), positive is to the right (in the direction of the curve). Dashed line is TP. The general pattern is orientation towards the curve apex / tangent point region, with clear *optokinetic pursuit* superimposed. Vertical angle indicates gaze to be in the far zone. For the subject in the first image (*top*) horizontal angle indicates that gaze is in the far zone beyond the tangent point, in the bottom image adjacent to the tangent point.

When looking at data from individual subjects as a time series (Figure 7) it is immediately clear that the picture is more complex than a single statistic giving “typical" gaze position would suggest.

An individual can thus be characterized by a “typical” gaze position (e.g. density mode as used here, see Figure S4 in Supporting Information S1), which is located in the far zone and often within a few degrees of the tangent point (But sometimes as much as ten degrees, see Figure 6). The modal gaze distribution can then be said to be “tangent point oriented”, but on gaze position alone it would be equally well to say gaze is “apex oriented” or “far zone oriented”

On average gaze is offset by some small constant from the tangent point (a few degrees), but this offset does not arise from a small *constant* deviation of measured gaze position from the tangent point. Instead, it is generated by a process where the gaze *moves* in a systematic manner rather than being located at the tangent point or some point offset by a constant angle. Clearly the typical gaze *position* does not characterize the eye movements of that individual.

From Figure 7 one can see quite clearly that ”fixations to the road” are not stable, relative to the egocentric frame of reference of the vehicle, but pursuit movements. This means that while the eye may be comparatively stable relative to the external environment, the “fixations” resemble tracking or pursuit movements in the direction opposite to the curve direction. In other words, far from being static, they instead have a large horizontal angular velocity component. Superimposed on the coarse level pattern of far zone / tangent point region orientation is a high frequency pattern of optokinetic pursuit and saccade (nystagmus).

**Figure 8:**
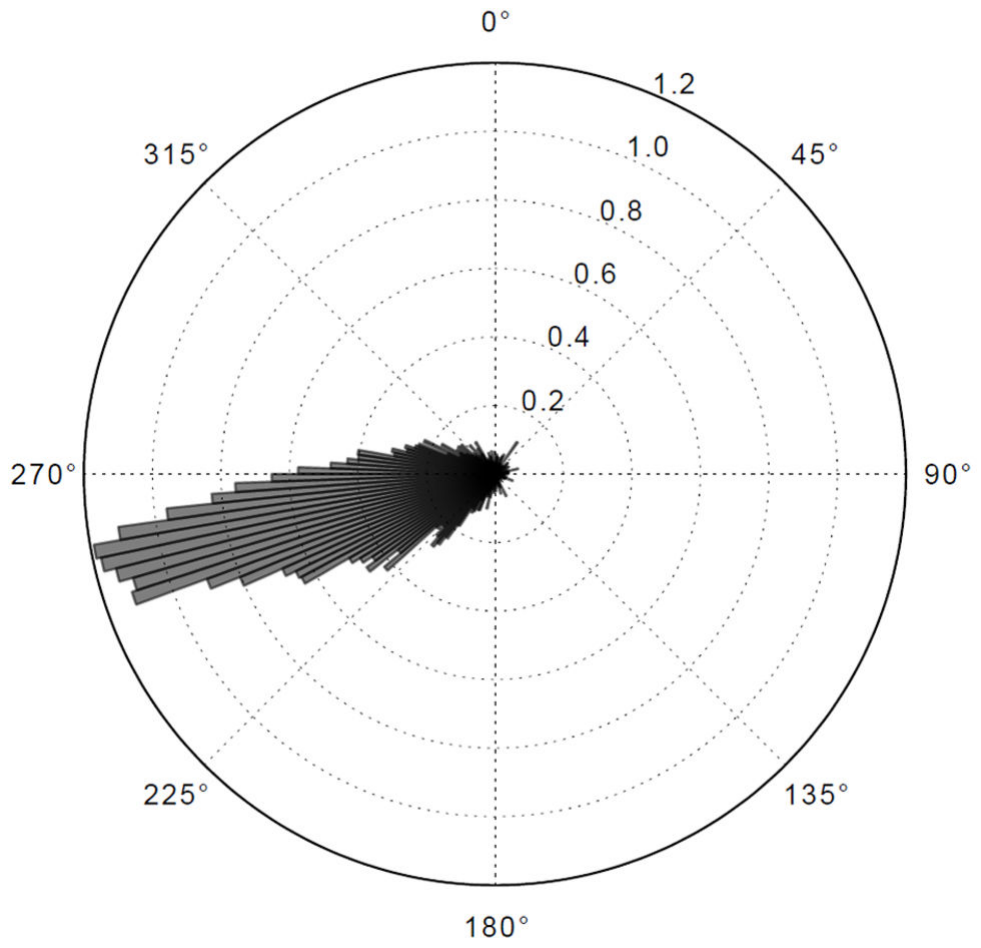
Orientation of OKN slow phases. Histogram of the orientation of all identified pursuit eye movements in the data. 0° is up. For individual subjects’ data see Supplementary Results.

What are the characteristics of this nystagmus? Figure 8 shows the distribution of SP orientation, which is clearly down and to the left. This pattern is highly consistent between subjects (individual subjects’ data is available in Supplementary Results in Supporting Information S1).

**Figure 9:**
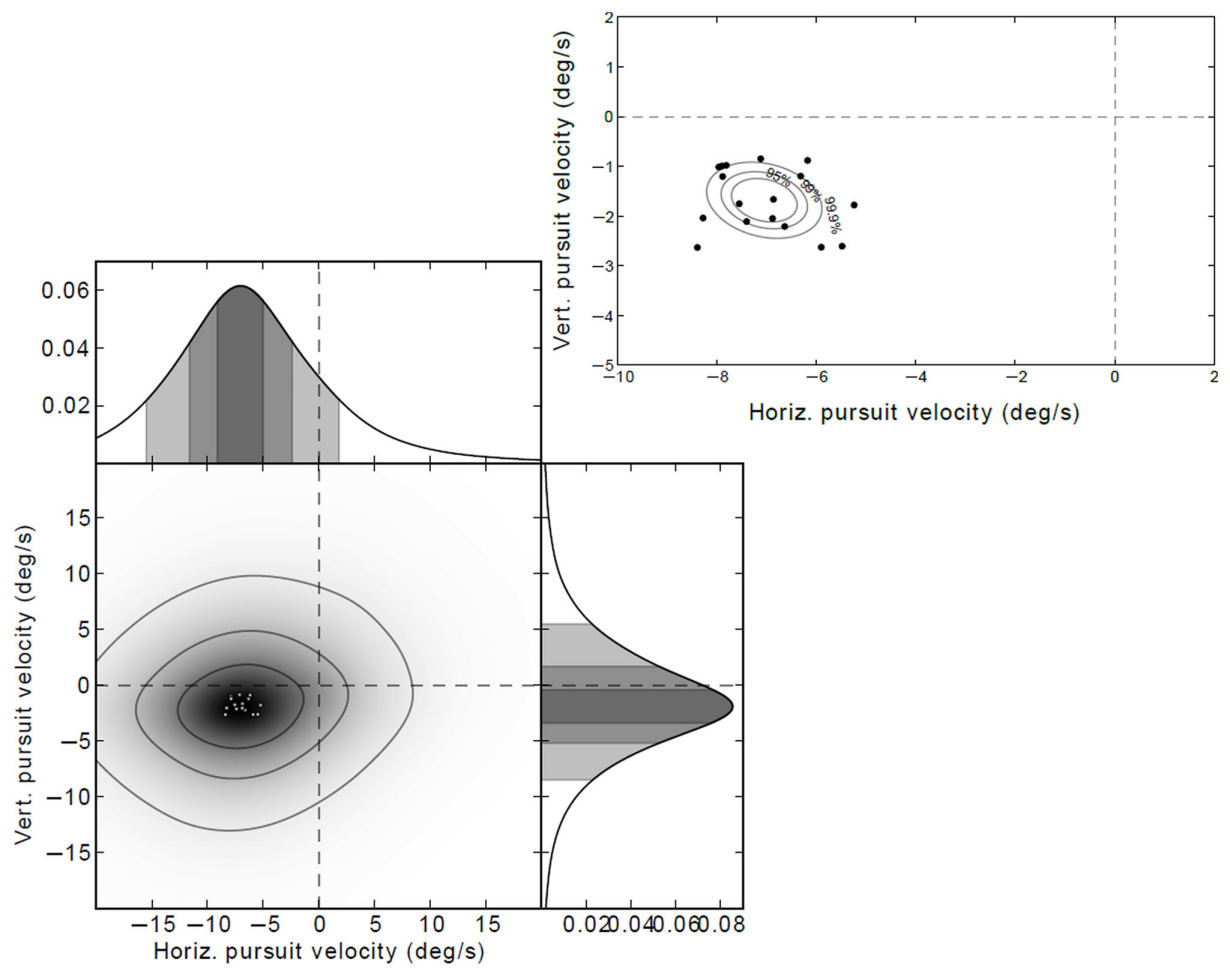
Density estimates of eye-movements plotted to velocity-velocity phase space. Density estimate and marginal density distributions (horizontal and vertical). Circles indicate individual mode values of individual subjects’ data. See Supplementary Results for individual subjects’ data. *Inset*: Enlargement, individual modes only. The ellipses indicate Hotelling’s T-squared 95%, 99% and 99.9% confidence regions. The distribution is not centered at the origin (which would indicate stable fixation data), but clearly clustered to the left (indicating a horizontal eye-movement component) and below (indicating a vertical eye-movement component). This is the pattern that one would expect from gaze following regional optic flow in the far zone.

To evaluate this pattern statistically, we represent the data as a density estimate distribution in the gaze velocity phase space (where the dimensions are horizontal and vertical components of gaze velocity, i.e. the slopes of the regression lines, negative horizontal and vertical velocity indicates movement down and to the left). We obtain the pattern shown in Figure 9 where the individual subjects modes (density estimate peaks) are all to the left (mode average -7.0 degrees/s) and down (mode average -1.7 degrees/s). See also individual subjects’ data in Supplementary Results in Supporting Information S1. The 95%, 99% and 99.9% Hotelling’s T-squared confidence regions for the mode of gaze velocity density clearly show that the distribution is significantly biased into the lower left quadrant.

This direction and magnitude of the pursuit is consistent with the measured gaze position, since the direction of the optical flow of the *road surface* in the far zone is down and to the left, and as the average vehicle yaw-rate varies between 12–16 degrees/s: future path models predict that the value of horizontal gaze velocity should be approximately half the horizontal rotational velocity of the vehicle. The lateral component of the pursuit is very close to that predicted from future path orientation, but difficult to reconcile with tangent point orientation.

This prediction is corroborated in our data also at the individual level, where per-subject heading velocity seems to correspond to half the per-subject average yaw-rate. The 95% CI for the mean of yaw-rate/2 – horizontal gaze velocity is (-0.4 °/s, 0.6 °/s), Shapiro–Wilk W = 0.98, p = 0.98. What is more the correlation between yaw-rate and horizontal gaze velocity, while small, is positive for every participant. The correlations are quite small (median 0.14), but it should be noted that variation in yaw-rate itself is in a very restricted range, because only a single bend was analysed, and in that bend only the cornering phase where the yaw rate remains very stable. So what is observed here is mainly an effect of small steering inputs and changes in speed. An experimental setting with variation of driving speeds and/or curve geometries would provide more robust evidence concerning this particular quantitative prediction.

With all methods for characterizing gaze behavior available to us we thus get converging evidence of orientation towards the far zone.

## Discussion

Our data indicate that during cornering, drivers’ gaze is concentrated in the far zone, adjacent to or often beyond the tangent point. This is shown in both gaz*e position* data (distribution of gaze displacement from the tangent point and modal gaze position relative to TP) as well as *eye movement* data which clearly show optokinetic pursuit movements consistent with the direction of optic flow in the far zone. This is predicted by future path models, but creates a complication from the point of view of the tangent point models. These results were found to be consistent at the level of individual subjects with considerable variation in driving experience.

We do not observe any clear indication that the drivers’ gaze is focused on a narrow region near the tangent point, which would be expected if the drivers are steering by the tangent point. Instead, the pattern is consistent with the future path oriented models [4,6]. The SP location and direction can be explained by assuming the drivers select target points on the future path (in the *far zone*) and track them for short periods. Analysis of OKN SP and QP in the eye-movement data (the direction and magnitude of optokinetic pursuit) is also consistent with the SP following the optic flow direction in the far zone, but not consistent with optic flow at TP, and difficult to reconcile with tangent point models.

This is not to say that, in light of our data, the tangent point hypothesis should be considered categorically false – but its domain of application may be much more restricted than has been heretofore assumed. We analysed gaze behavior in the *cornering* phase of the bend. By contrast, most studies which have reported substantial “tangent point orientation” have focused mainly on the *approach* and *entry* phases.

In the seminal study [1] Land and Lee found that the tangent point was fixated “before the car enters the bend”, and the fixation density at the TP was highest immediately after turn-in and up to about “~3 s into the bend”. As the cornering sequence progressed, the relative frequency of fixations in the general direction of the tangent point direction decreased which is clear from the figures. We studied the steady-state cornering phase (absent on a tight, winding road such as the one used in that study), which begins only after about 5 s and lasts in this case for 16-18 s. The most conservative interpretation of the difference between our results and the original tangent point orientation results would then be that distinct steering strategies may characterize different phases of the curve driving sequence: the tangent point could be important for the decision to initiate the turn, and for judging the appropriate amount of steering. Once the driver has entered a sustained, constant radius part of the bend, however, the tangent point seems to play little role in steering control and visual anticipation, according to our data.

## Conclusions

We find that gaze position is consistently above the tangent point, and at a population level the distribution quite spread out. What is more, the spread is in the direction of the turn, and the gaze position observations are much higher, vertically, in the higher eccentricities, consistent with the road being an uphill right hand bend. We also find that eye movements have a clear OKN pattern, and that this OKN is consistent with coherent optic flow in the area where the gaze appears to land (the far zone adjacent to and beyond the tangent point): it is to the left and downward, and the magnitude is very closely one half the vehicle yaw-rate. Of course, without an explicit model of the true regional optic flow at exact gaze landing point, this approximate correspondence is largely qualitative.

We interpret our data as corroborating evidence for the casual observation that car drivers often look at the road, where they are going (notwithstanding that for 20 years the default hypothesis has been that drivers do not look at the road, but at the road edge at a point where they are not going).

Tracking fixed target points on the future path in the far zone with pursuit eye movements would predict *gaze position* to be typically above, and display a *large horizontal variation in relation to the tangent point*, being spread into the far zone along the future path. The optokinetic pursuit should have a horizontal component in the direction opposite to the bend and the *magnitude of the horizontal component of the pursuit movements* should be approximately half vehicle yaw rate. We observe all these things.

Fixation of the tangent point would predict gaze to be located at the tangent point (within a few degrees). This is not the case. The gaze is clearly spread out in the far zone, and this is not only due to “smearing” of the gaze position distribution when all the gaze positions of the pursuit movements are collapsed by removing the time dimension. There are individual differences in where in the far zone the OKN SP’s are located.

Incorporating the “disturbance” created by OKR into tangent point models means that the predicted TP fixation may not be stable, but that there is local OKR superimposed on the global TP orientation, in the face of which the driver may be unable to maintain accurate foveation of the tangent point. Local optical flow around the point of regard (*ex hypotesi* in the tangent point region) “captures” gaze, and causes an involuntary (reflexive) pursuit. This takes the gaze away from the tangent point, and if the driver is “trying” to fixate the tangent point, this deviation would be expected to be followed by correcting saccades. While this could create a periodic eye-movement pattern resembling optokinetic nystagmus, one would not expect to find the strong horizontal component clearly present in our data (as well as the simulator data of Authié & Mestre [9]).

Accommodating the observation that the direction and magnitude of OKN SP matches optic flow *beyond* the tangent point – but not *at* the tangent point – requires that one assumes that (i) the subject is “trying” to fixate the tangent point but fails because of an OKR reflex elicited by optic flow that creates pursuit movements, and that (ii) the OKR also fails to match the direction or magnitude of flow at the gaze target (TP) location, possibly because it is “confused” by the complex flow pattern. These assumptions make the model more complex without predicting any new data. To make the integration of this finding into tangent point models more than a *post hoc* explanation requires some means to determine the dependence of OKN SP on regional flow that “should” occur during TP tracking. Either a theoretical argument form first principles or an independent empirical demonstration. At this point in time a tangent point model that would incorporate a specific hypothesis of the OKR remains yet to be developed (e.g. what is the shape and size of the local flow field that the OKR SP “should” follow, and does the SP terminate at TP or does the QP terminate at TP). Also, the ability to track the tangent point (suppress OKR) should be derived from known properties of the smooth pursuit/fixation system or determined experimentally.

Interestingly, when explicitly instructed to “stare” at the tangent point (maintain constant fixation for several seconds), the subjects in [13] were reported to comply “for more than 90% of the time”. This seems to suggest that it would indeed be possible to suppress the OKR successfully to maintain fixation on TP when instructed to do so. However, presence OKN was not analyzed in that study, so the question remains open (Also, even if this was the case, this is not the normal pattern observed in free gaze conditions).

We conclude that as long as TP models do not derive OKN SP parameters by theoretical argument, or unless they are established by analysis of the independently verified properties OKR system, the presence of horizontal SP and its being consistent with the coherent regional optic flow around the future path has to be considered to be in favor of FP models.

All of the above notwithstanding, we agree that the tangent point is occasionally foveated, and an important reference point in the visual field. Our main conclusion is that during steady state cornering gaze seems to already poll the road well ahead, with scant overt visual attention paid to the tangent point.

This is not to say that the tangent point might not be more often looked at while *approaching* and *turning into* the bend, which is what was reported by Land & Lee ( [1]; see also 13). However, the close proximity of the tangent point and the future path in these curve phases makes such AOI results ambiguous as evidence for TP vs. FP as gaze targets. Even if corroborated by complementary analysis methods – perhaps similar to the ones used here – this would at minimum suggest that different steering processes are involved in transitional steering (reported in the previous studies) and steady state steering (reported here). Hence, at minimum we would conclude that steering-by-TP does not give a comprehensive picture of visual orientation throughout the entire sequence through a bend (Actually, we would assume it is used particularly when judging appropriate entry speed and imminent changes in path curvature).

This points to the complexity of driver behavior: multiple gaze targets are present (perhaps at different parts of the curve), which implies that a single, simple, overarching control law purporting to explain “how we steer” is likely to be insufficient to describe the control of high speed steering in humans.

According to the tangent point models, optokinetic pursuit indicates that there are two opposing visual mechanisms at work during tangent point orientation. On the one hand, the visual system is ‘trying’ to *foveate* the TP. On the other hand, it is at the same time opposed by an *optokinetic reflex* pulling gaze into the direction of optic flow. The mechanisms maintaining fixation of the tangent point (or pursuit since foveation of the tangent point requires active tracking due to the lateral movement of TP) would thus be working *against* a lower level reflex. The tangent point hypothesis, then, assumes that the optokinetic nystagmus observed is generated in an *antagonistic* manner relative to the steering strategy (and, for some reason, the oculomotor system does not or cannot inhibit this counterproductive reflex action).

From the point of view of models positing future path target points, the situation looks rather different. If the driver is “trying” to look at the road, the neural circuitry generating the optokinetic pursuit movement can be recruited into the ongoing task. This circuitry can be a low level OKR, driven by retinal image slip or a higher-level predictive pursuit generator. The optokinetic reflex can moreover be considered to be recruited to aid in the active gaze pattern required for the steering strategy and *integrated* into the pattern of flow of visuomotor behavior in a synergistic manner. A point of regard on the road would be chosen as target location, designated by making a saccade to it. At this point the optokinetic reflex could "take over" maintaining foveation of the desired target point on the future path (in stereoscopic viewing the region determining the OKN could be the plane of fixation, which the visual system can retrieve from zero retinal disparity). A saccade would then designate a new point and the cycle would repeat. According to this view, the reflex does not need be inhibited at all, because the eye movement created by the reflex is in fact the eye movement that is required by the steering strategy.

## Supporting Information

Information S1 Supplementary methods, supplementary results, including individual trial data, and supplementary discussion outlining a more detailed qualitative comparison of various gaze strategies and their predictions. (PDF)

Movie S1 (MP4)

Movie S2 (MP4)

Movie S3 (MP4)
