# Supplementary material for: Pursuit Eye-Movements in Curve Driving Differentiate between Future Path and Tangent Point Models
Source: PLoS One. 2013 Jul 22;8(7):e68326. doi: 10.1371/journal.pone.0068326 (PMC3718775; doi:10.1371/journal.pone.0068326)

PLoS ONE doi: 10.1371/journal.pone.0068326

SUPPLEMENTARY MATERIAL

for Lappi O., Pekkanen J. & Itkonen T.:

Pursuit Eye-movements in Curve Driving

Supplementary Methods

Calibration & gaze quality

A nine-point calibration was performed by asking the subject to look at designated objects in the scene outside the garage. Successful calibration was verified by asking the subject to fixate the same objects again. If the online visualization of gaze position for some calibration points was off (by about two degrees or more), a recalibration was performed. On the road, maintaining calibration was verified by visual judgment between each run, by designating objects for the subject to look at. A gaze quality criterion of 0.2 supplied by the tracker software was used to exclude data before analyses.

Supplementary figure S1

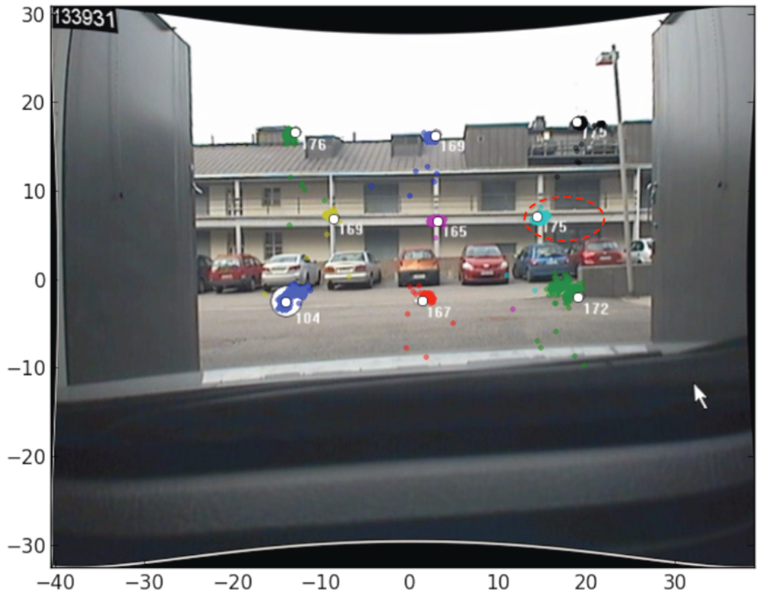

Nine point calibration used to calibrate the eye-tracker. White dots are placed at the designated calibration points. The colored dots represent data-points of gaze position. The red ellipse indicates the approximate part of the visual field where the tangent point falls in during cornering.

SUPPLEMENTARY FIGURE S2

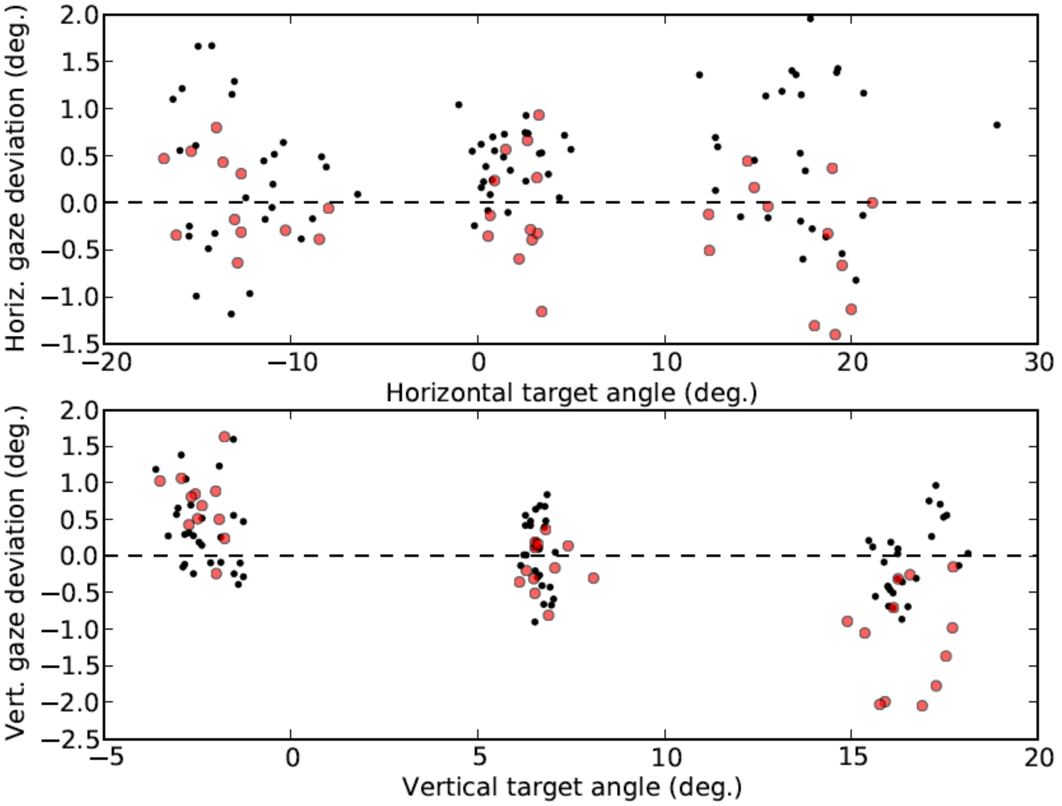

Horizontal and vertical, gaze deviation (difference of median observed gaze position from designated target point) is under 2°. Black dots: calibration datapoints from the current dataset. Open red circles: calibration datapoints from another simultaneously collected dataset.

Mathematical description of the segmentation algorithm

The system aims to maximize a fitness function, although it is not known if it actually reaches a (global) maximum:

$$L(S) = \sum_{s \in S} \left(P(s_0, \lambda) + \sum_{i \in s} \left(G(\hat{x}_i - x_i; 0, \mathbf{\Sigma}) 1_{i \notin O} \right) \right) + \sum_{i \in O} cP(i, \lambda)$$

where P is logarithm of the Poisson survival function for more than zero events with rate parameter λ for a new segment with i being time between samples i and $i-1$, s_0 denotes the first sample index in the segment, G is logarithm of the Gaussian probability density function with mean zero and (diagonal) covariance matrix $\mathbf{\Sigma}$, O is the set of outliers and c is a

“penalty coefficient” for outliers, is the signal value of sample and is its estimate based on the segment’s linear fit.

For the present analyses we used = 1/0.5 and = 0.6 based on tuning by hand. was iteratively estimated similarly to the Expectation Maximization method by calculating the ML estimate based on a run of the algorithm and then running it again with the new estimate until the segmentation does not change. We used initial noise variances of 1.0 for both dimensions.

Supplementary Results

Driving behavior

The following figures and tables quantify physically driving behavior in the cornering phase in the present study. The Supplementary Figure S3 and S4 display group level and individual driving speeds as a function of lap. Supplementary Tables T1 and T2 show individual participants’ yaw-rate and the eccentricity in the visual scene.

SUPPLEMENTARY FIGURE S3

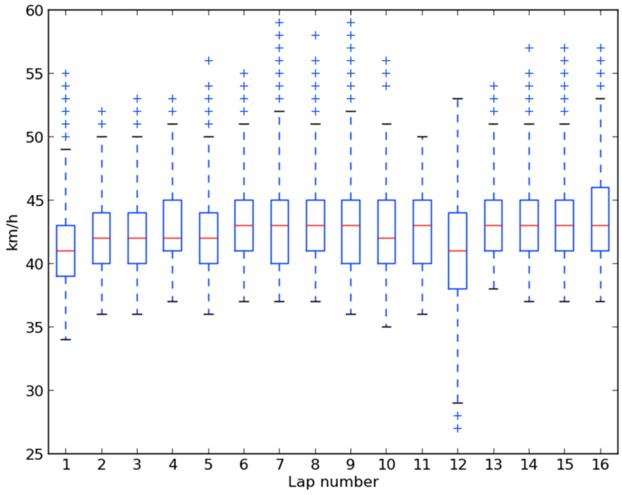

Boxplot showing average driving speed in the cornering phase as a function of lap.

SUPPLEMENTARY TABLE ST1

Per subject vehicle yaw rate in the cornering phase (Mean, SD)

Participant	Yaw-dot M	Yaw-dot SD
1	13.5	1.2
2	14.6	0.9
3	13.5	1.2
5	12.9	0.9
6	12.8	1.3
7	13.6	1.5
10	12.9	1.1
11	13.9	1.1
12	14.2	1.2
13	14.0	0.9
14	14.1	1.0
15	13.9	0.9
16	13.6	1.0
17	14.6	1.3
18	16.2	1.7
19	13.0	0.8
21	14.7	1.5

SUPPLEMENTARY TABLE ST2

Horizontal angle of TP in vehicle centered coordinates during cornering (Mean, SD).

Participant	TP° M	TP° SD
1	16.7	1.4
2	18.3	1.1
3	19.5	1.3
5	19.4	1.2
6	19.4	1.0
7	18.4	1.7
10	18.0	1.2
11	18.9	1.2
12	16.1	1.5
13	16.6	1.1
14	17.8	1.0
15	17.7	1.2
16	16.9	1.5
17	14.1	1.6
18	16.1	1.6
19	15.1	1.7
21	17.0	2.7

PLoS ONE doi: 10.1371/journal.pone.0068326

Supplementary Figure S4

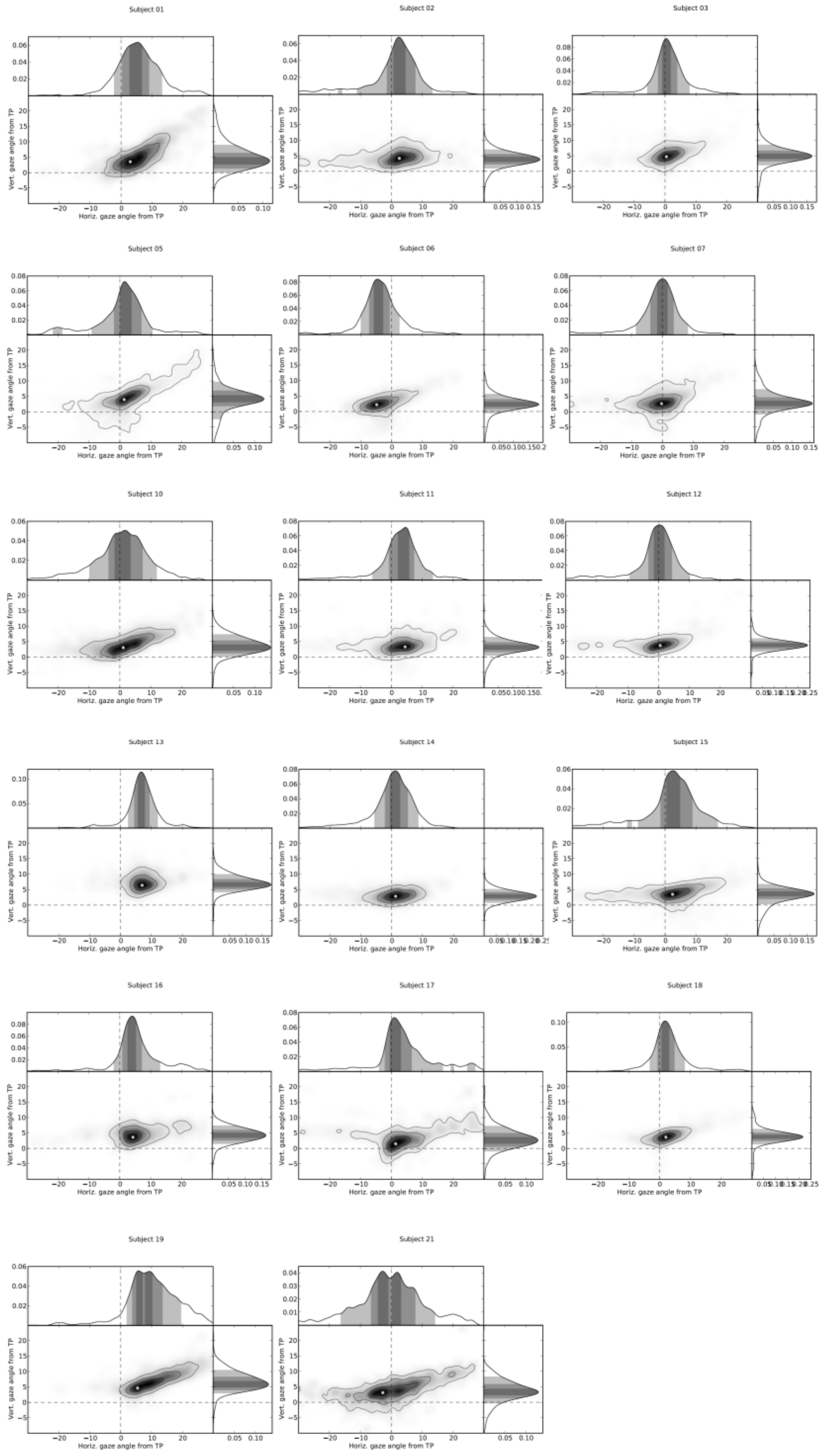

Density distribution of gaze displacement from the tangent point, with marginal distributions. The shaded areas represent highest-density regions with 75%, 50% and 25% thresholds. Individual subjects' data.

Supplementary Figure S5

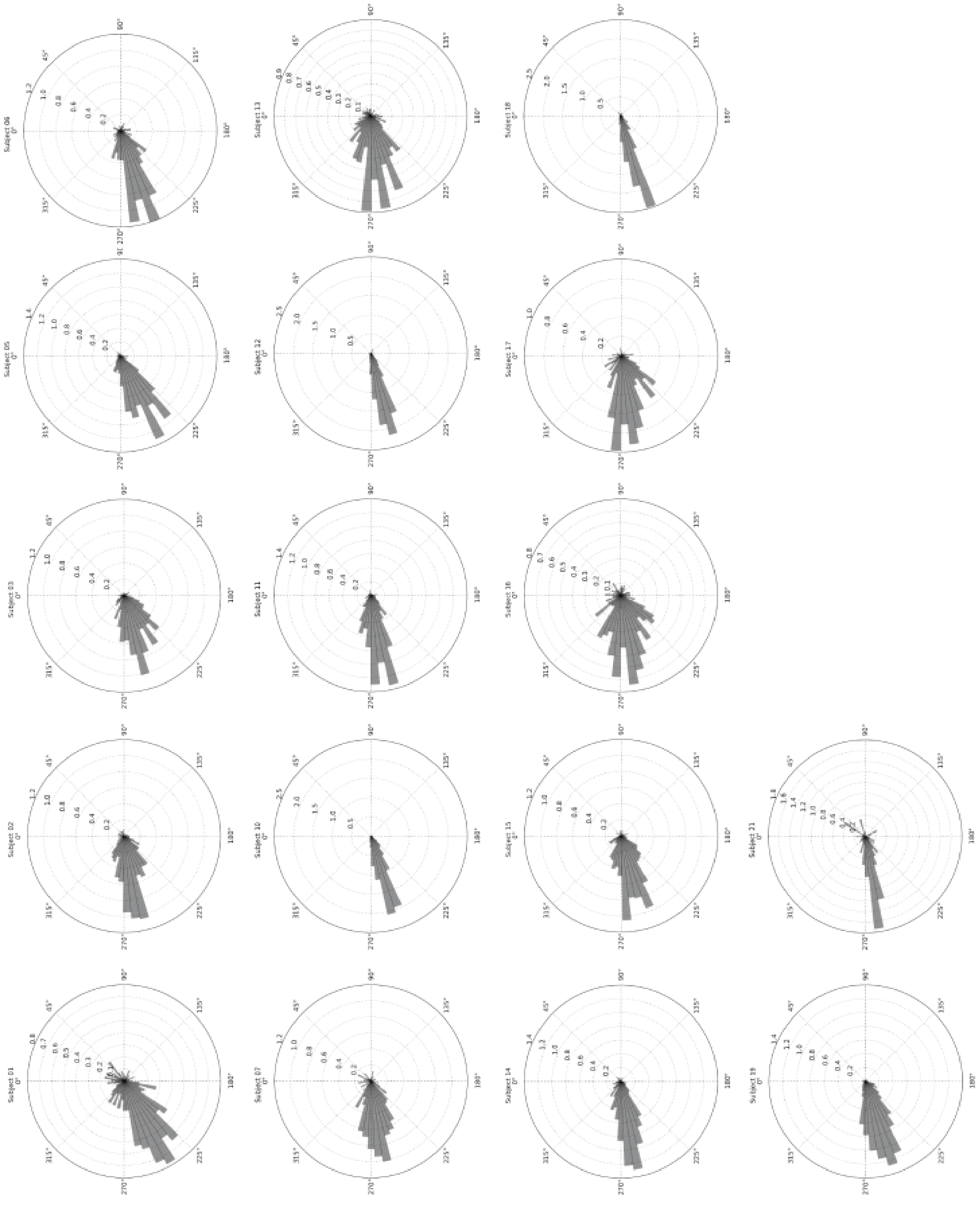

Histograms of the direction of each participants' pursuit eye movements.

PLoS ONE doi: 10.1371/journal.pone.0068326

Supplementary Figure S6

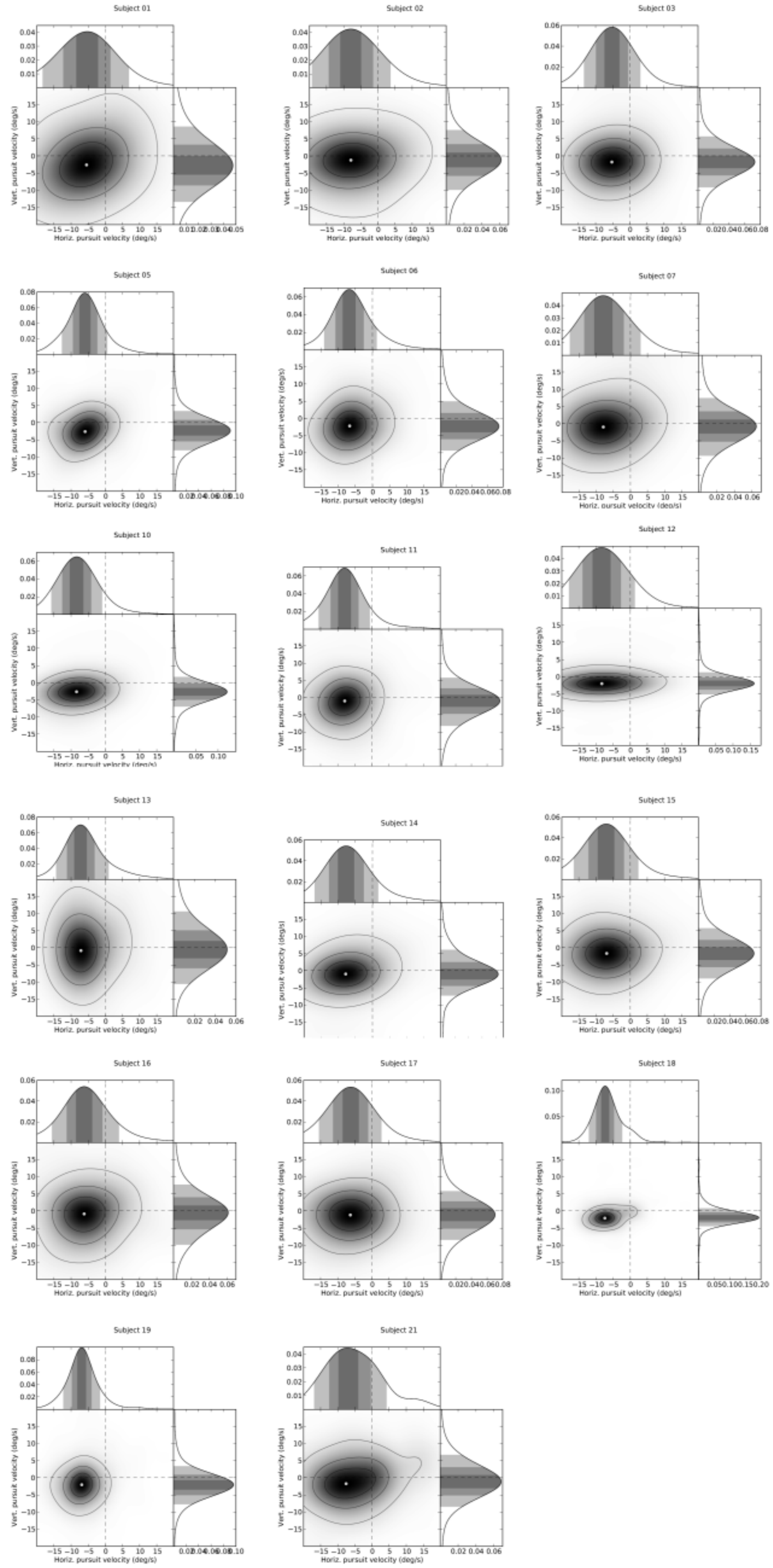

Density distribution (in velocity-celocity phase space) of horizontal and vertical gaze velocity. The shaded areas represent highest-density regions with 75%, 50% and 25% thresholds. Individual subjects data.

Supplementary Discussion

In this appendix to the discussion on the different models, we outline the different alternative predictions open to TP and FP models concerning gaze position and eye-movements (changes in gaze position). The derivations are explained in more detail here because, given that OKN was only recently demonstrated, most of the models do not discuss it explicitly. Especially reasoning out behind how OKN SP and QP "should" behave when the tangent point is being tracked is tricky because by the qualitative nature of the models and parameters of eye movement behavior cannot be derived quantitatively.

Tangent point models a postulate that

1. tangent point orientation results from a visual strategy where drivers *track the tangent point.* (rather than contiguous points on the future path)
2. the tangent point is tracked *because* it provides preview information of road geometry relevant to adjusting steering

Future path models posit that:

1. a *target point on the future path is tracked* because it provides preview information of road geometry relevant to adjusting steering
2. tangent point orientation is mainly a result of contiguity of the future path reference point(s) and the tangent point.

Assumptions of exact gaze target combined with known properties of optical flow and the assumption that *optokinetic pursuit follows regional optic flow regardless of which target point is being visually tracked* point to new ways of assessing the tangent point and the future path as drivers' gaze target in.

OKN & tangent point models

TP Hypothesis 0. The default prediction from targeting the TP would be that fixation is stable at the TP, and the flow pattern around the tangent point would not affect the rotation of the eye. It is currently not known from experiment whether it is possible for human subjects to suppress OKN while looking at the TP.

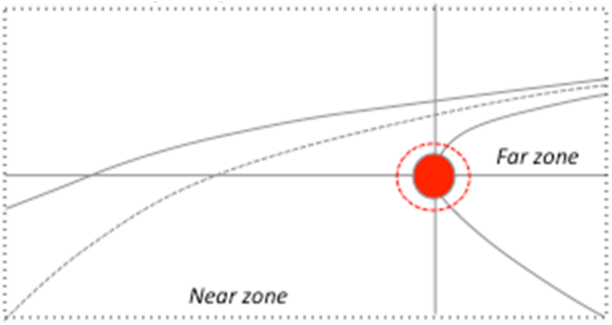

TP Hypothesis 0 (no OKN). Persistent fixation of the tangent point. Gaze is stable at the TP. Possibly observed in the TANG condition in Kandil et al. (2009) – although the presence or absence of OKN was not analysed in that study – but not in everyday driving.

That OKN is reliably elicited, however, shows that either the OKR is present while the TP is fixated (or that the drivers are not looking at the TP).

If the drivers' "attemp" to fixate the tangent point is hindered by OKR elicited by regional flow, gaze would move away from the fixation target and require re-setting saccades to restore fixation (hence OKN QP). QP characteristics may be therefore predicted if the dependence of SP on regional flow is known.

TP Hypothesis 1. Under the assumption that the OKR follows local flow, QP could re-set gaze to the tangent point (assuming the SP has drawn gaze away from it), or to launch gaze "upstream" in the flow field, so that the slow phase pursuit OKR will bring gaze back to the TP.

Because the tangent point falls on the line of inversion (zero crossing) of the horizontal component of optic flow, the flow at the tangent point is vertical (downwards). The simplest prediction would then be that the SP pursuit movements follow the local flow at the point of regard which, with perfect TP fixation and the vertical local flow at the tangent point would means a vertical downward SP, and a vertical upward QP.

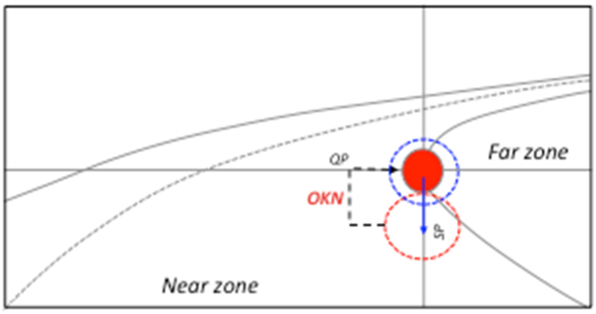

TP Hypothesis 1. OKN SP following local flow at the TP (vertical), with re-setting QP. A pattern not observed in the present study or Authié & Mestre (2011).

A vertical OKN is *not*, however, what is observed. Neither in the present study nor in Authié & Mestre (2011) study. Instead, a large horizontal component against the direction of the curve is observed.Therefore is must be concluded that *either* gaze does not follow local flow, or else drivers do not fixate the tangent point, but a point on the road beyond (where the flow does have a large horizontal component).

TP Hypothesis 2. If gaze is targeted at the tangent point, but is not stable at the tangent point because of OKR. But the as the SP does not follow local flow (it has a horizontal componens) the hypothesis needs to be adjusted.

The dependency of OKN SP on regional optic flow is not clear, and the assumptions of the TP hypotheses (above) do not give a specific prediction. Empirically, it is known that it is *opposite* to the direction of the curve and downwards. Thus,

although local flow at TP is downwards, the OKR would be affected by regional flow elsewhere, in particular above the TP:

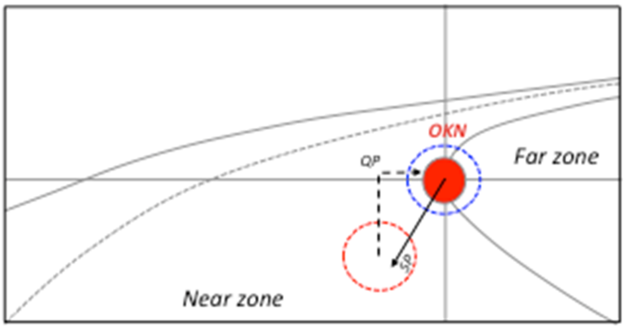

TP Hypothesis 2. Optic flow "captures" gaze. The direction of OKN SP does not follow local flow at TP, but flow of some region around the TP. This region needs to be determined to predict OKN behavior quantitatively. Empirically, it is known that the SP in fact takes gaze downwards and to the left.

TP Hypothesis 3. Another possibility would be to launch gaze "upstream" in the flow field, so that the slow phase pursuit OKR will bring gaze back to the TP:

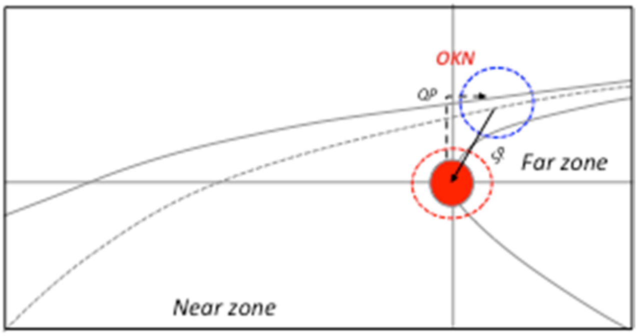

TP Hypothesis 3. Gaze is cast "upstream" in the flow field. OKN following (regional) optic flow re-sets gaze to tangent point.

There are thus many ways in which targeting the TP and OKN could be combined. Unless the size and shape of the relevant region assumed to determine the OKN SP need to be incorporated SP direction and magnitude is underspecified.

OKN & future path models

If a point on the future path is tracked the slow phase component of OKN should track a fixed target location. QP's are saccades to a new target location.

FP Hypothesis 0. Tracking a fixed location does not necessarily lead to OKN. If the location is tracked for several seconds (as in the "gaze sampling" condition in Kandil et al. 2009), a visual sweep is performed instead:

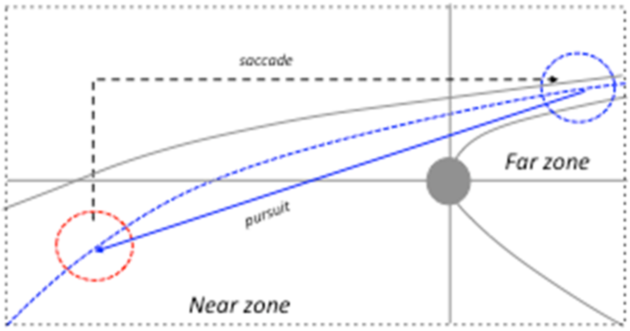

FP Hypothesis 0 (no OKN). Visual sweep of a future path target point. This gaze behavior was given as an instruction in an experimental manipulation by Kandil et al. (2009), but is not observed in normal driving.

The future path presents no immediately apparent *singular* reference point, and to determine OKN a model needs to specify where on the FP gaze is expected to land (the regional flow on the FP is not the same everywhere)

FP Hypothesis 1. In Boer's (1996) model, the target is selected adjacent to the tangent point:

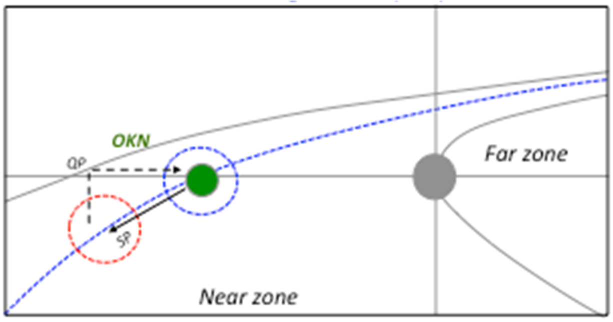

FP Hypothesis 1. Target point next to the tangent point is selected and tracked by a pursuit movement.

FP Hypothesis 2. We favor a hypothesis where a reference point in the Far Zone beyond the tangent point is used:

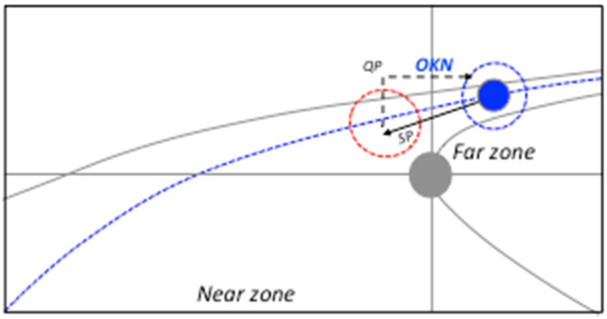

FP Hypothesis 2. OKN in the Far Zone beyond the tangent point.

FP Hypothesis 3. The two FP hypotheses can be integrated into a *gaze polling* model:

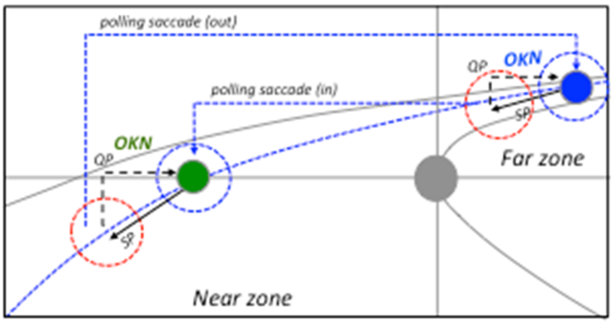

FP Hypothesis 3. Gaze polling in the far zone. Cf. Figure 1, middle, in main article.

APPENDED TO THIS SUPPLEMENT ARE INDIVIDUAL TRIAL RAW DATA SHOWING FIT OF THE SEGMENTATION OF THE LINEAR PURSUIT MODEL (SEE FIGURE 4 IN MAIN TEXT FOR EXPLANATION)

Subject 01, lap 1, noise std h: 1.195, v: 1.203

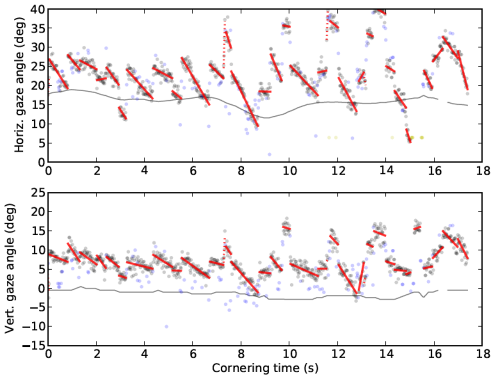

Subject 01, lap 2, noise std h: 1.202, v: 1.485

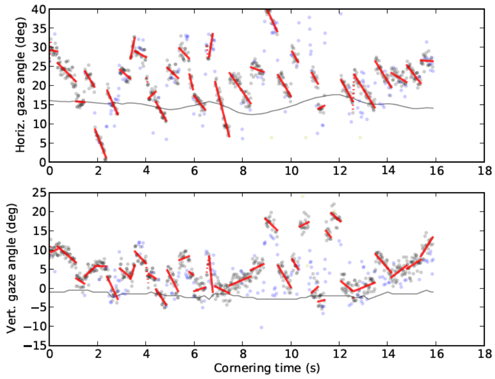

Subject 01, lap 3, noise std h: 1.302, v: 1.392

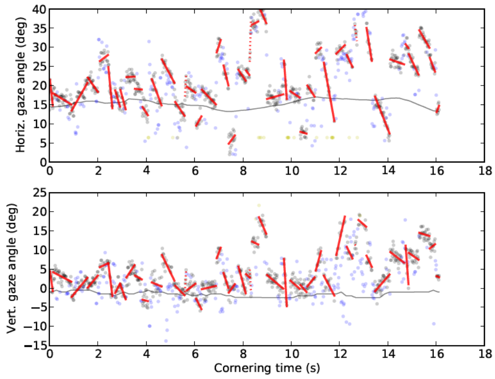

Subject 01, lap 4, noise std h: 1.159, v: 1.444

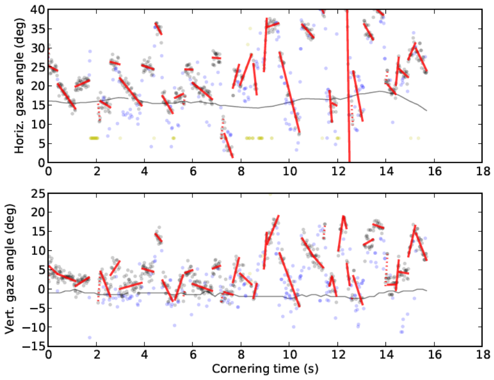

Subject 01, lap 5, noise std h: 1.253, v: 1.380

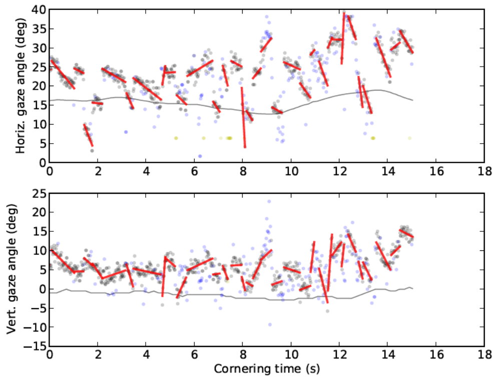

Subject 01, lap 6, noise std h: 1.214, v: 1.387

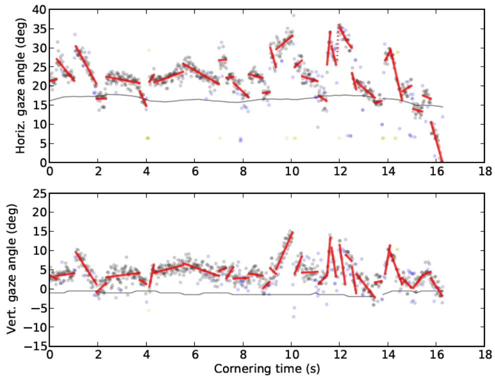

Subject 01, lap 7, noise std h: 1.419, v: 1.293

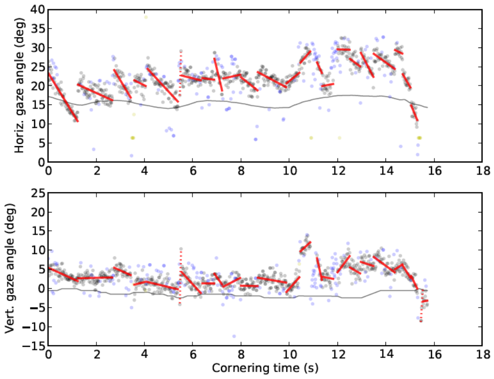

Subject 01, lap 8, noise std h: 1.395, v: 1.356

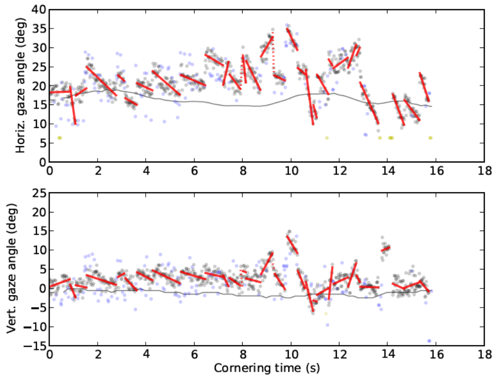

Subject 01, lap 9, noise std h: 1.328, v: 1.422

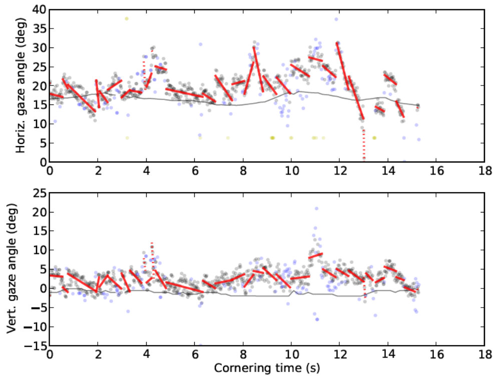

Subject 01, lap 10, noise std h: 1.274, v: 1.258

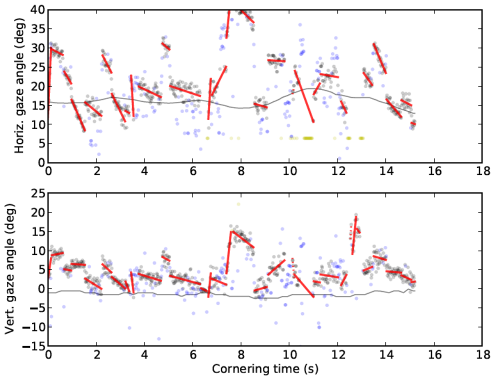

Subject 01, lap 11, noise std h: 1.412, v: 1.424

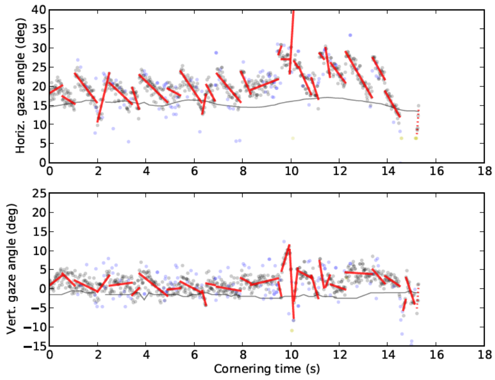

Subject 01, lap 12, noise std h: 1.300, v: 1.273

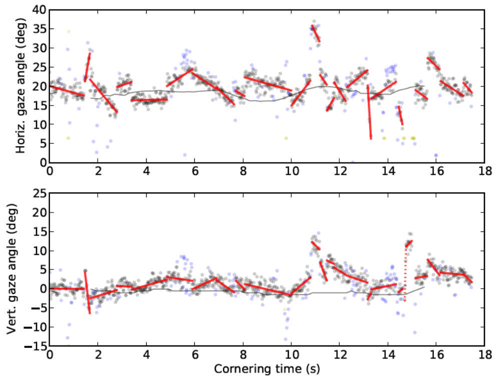

Subject 02, lap 1, noise std h: 1.016, v: 1.125

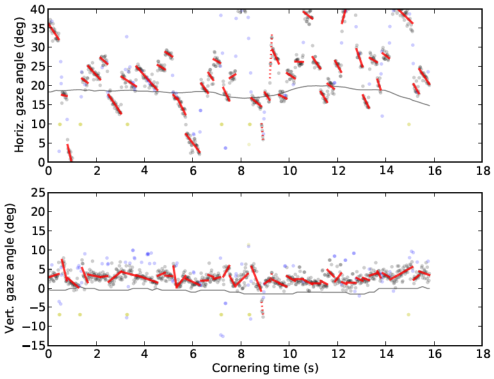

Subject 02, lap 2, noise std h: 0.960, v: 1.155

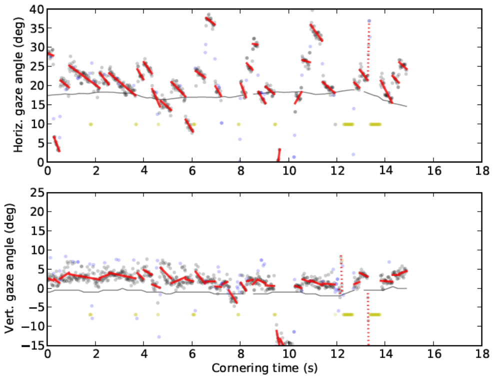

Subject 02, lap 3, noise std h: 0.967, v: 1.215

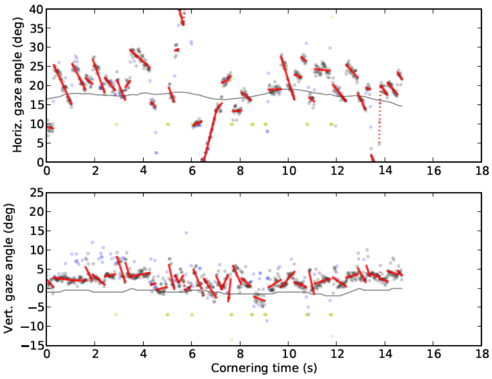

Subject 02, lap 4, noise std h: 1.002, v: 1.176

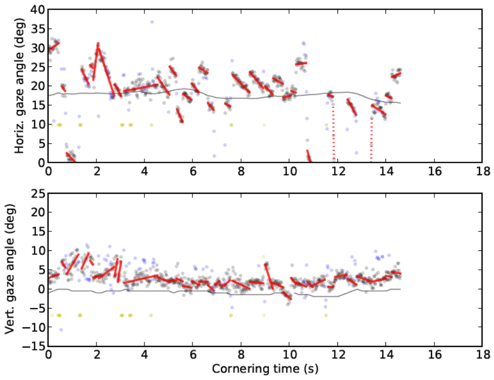

Subject 02, lap 5, noise std h: 0.974, v: 1.252

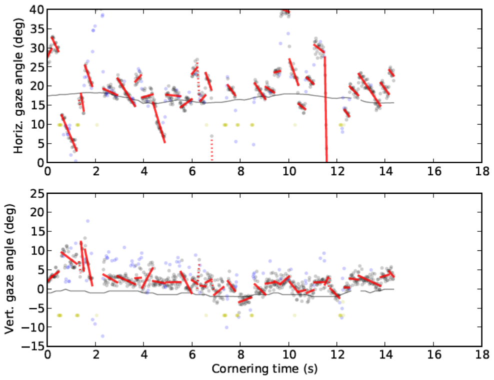

Subject 02, lap 6, noise std h: 1.004, v: 1.038

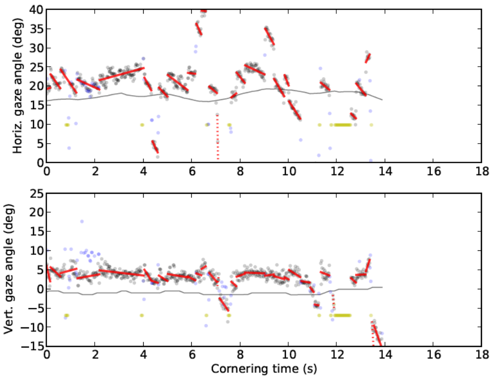

Subject 02, lap 7, noise std h: 0.899, v: 1.036

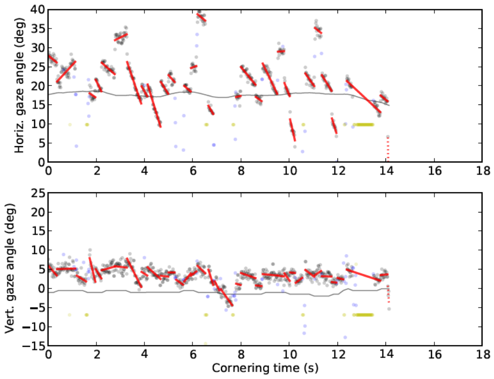

Subject 02, lap 8, noise std h: 1.107, v: 1.258

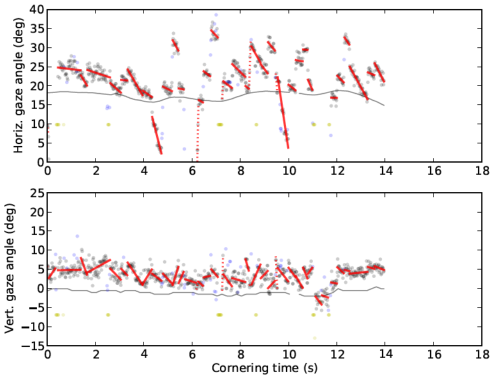

Subject 02, lap 9, noise std h: 1.048, v: 1.206

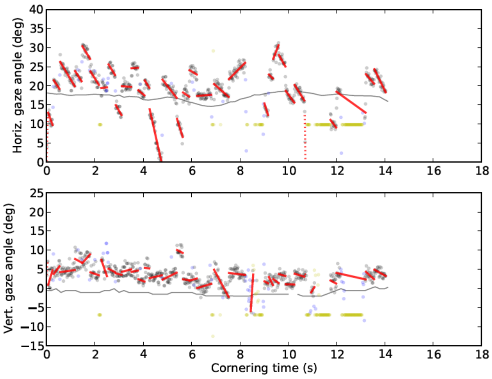

Subject 02, lap 10, noise std h: 0.981, v: 1.209

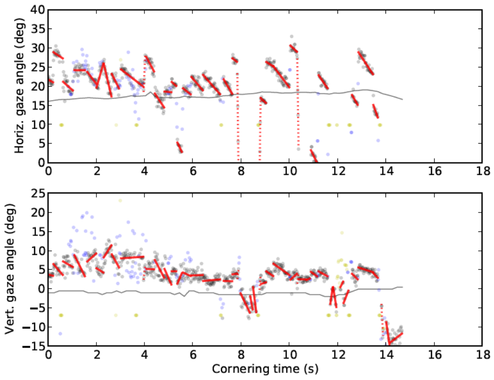

Subject 02, lap 11, noise std h: 1.159, v: 1.234

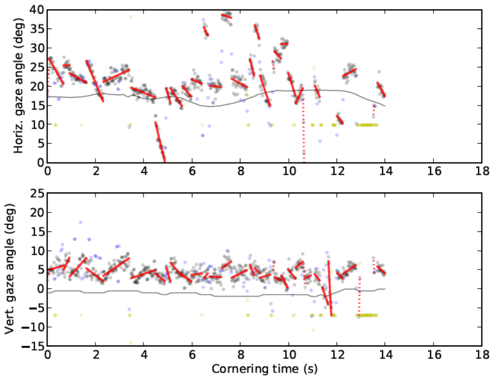

Subject 02, lap 12, noise std h: 1.003, v: 1.154

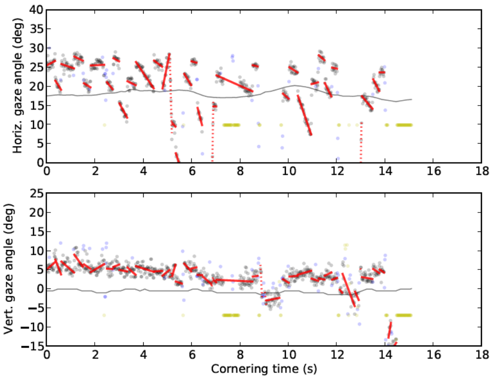

Subject 02, lap 13, noise std h: 1.072, v: 1.248

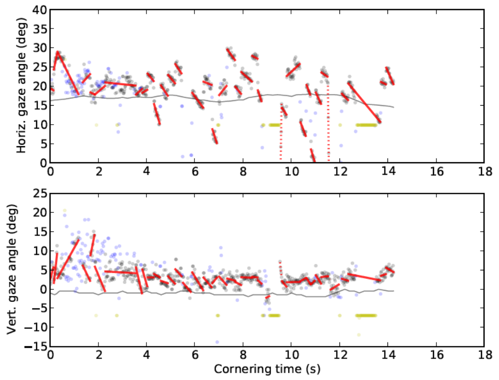

Subject 02, lap 14, noise std h: 1.044, v: 1.238

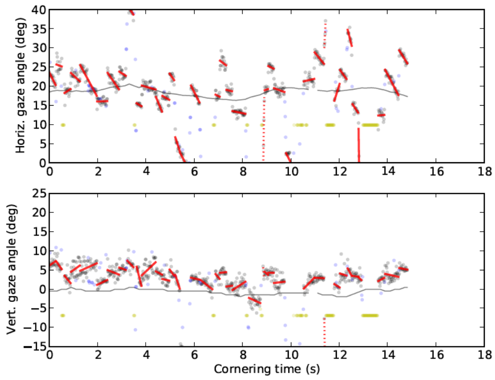

Subject 02, lap 15, noise std h: 1.023, v: 1.201

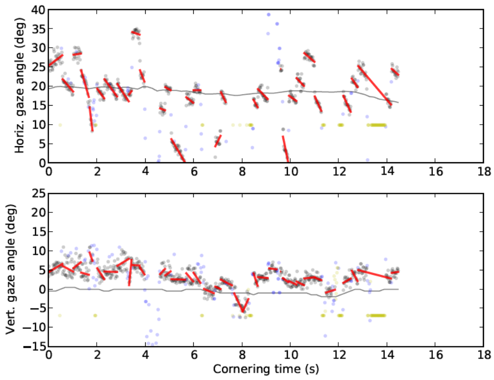

Subject 02, lap 16, noise std h: 1.003, v: 1.059

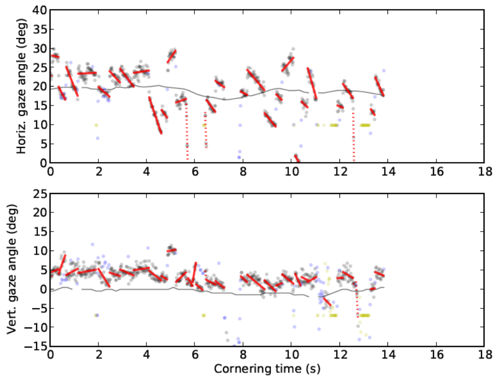

Subject 03, lap 1, noise std h: 1.028, v: 1.024

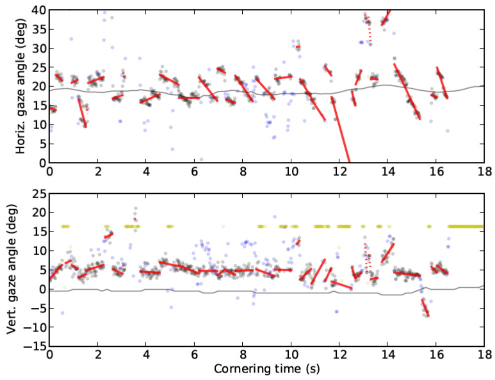

Subject 03, lap 2, noise std h: 0.945, v: 1.090

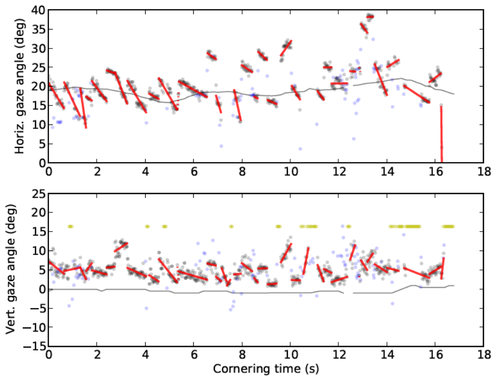

Subject 03, lap 3, noise std h: 0.916, v: 0.925

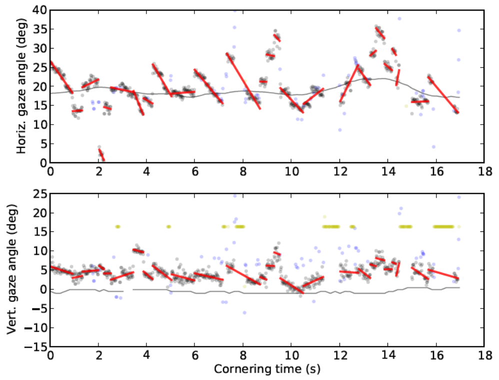

Subject 03, lap 4, noise std h: 0.866, v: 0.978

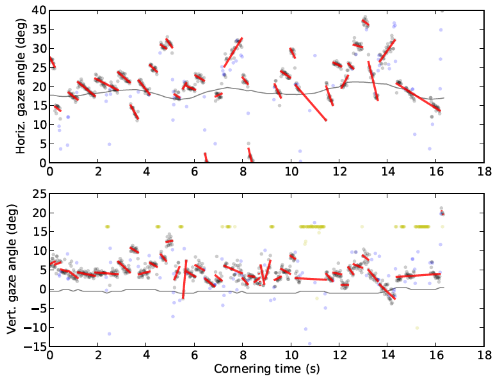

Subject 03, lap 5, noise std h: 0.895, v: 1.107

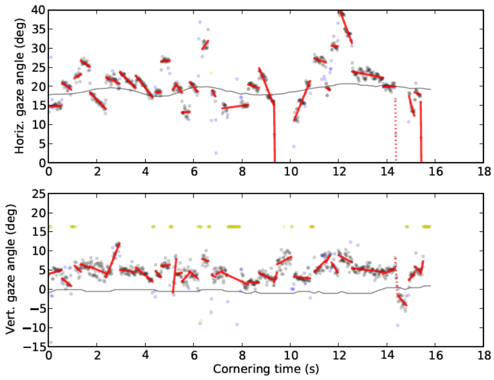

Subject 03, lap 6, noise std h: 0.786, v: 1.062

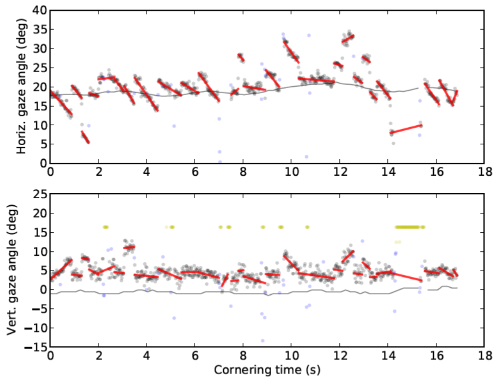

Subject 03, lap 7, noise std h: 0.877, v: 1.125

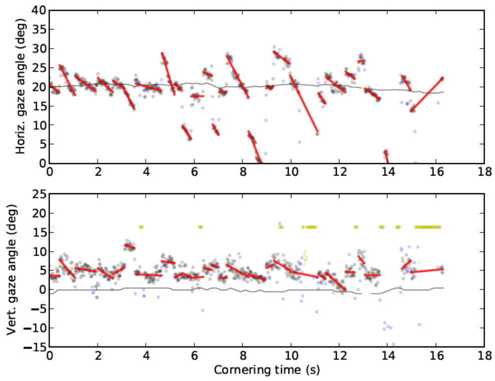

Subject 03, lap 8, noise std h: 0.816, v: 1.083

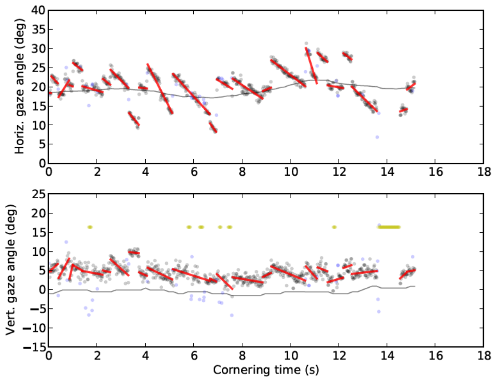

Subject 03, lap 9, noise std h: 1.054, v: 1.185

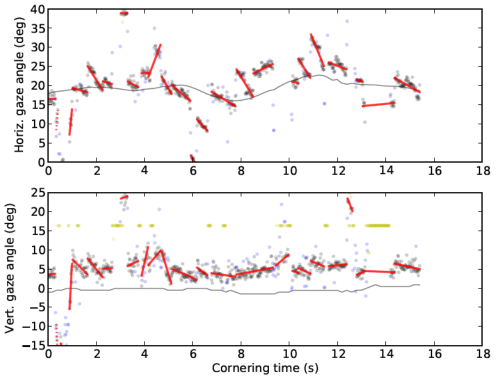

Subject 03, lap 10, noise std h: 0.879, v: 1.209

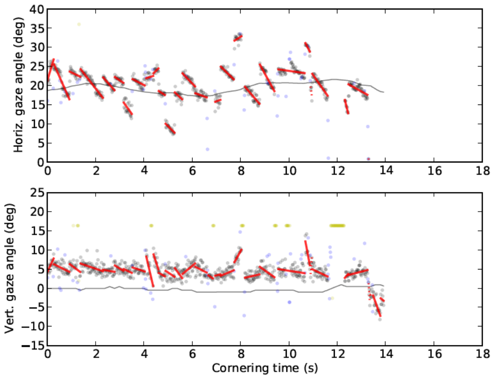

Subject 03, lap 11, noise std h: 0.935, v: 1.197

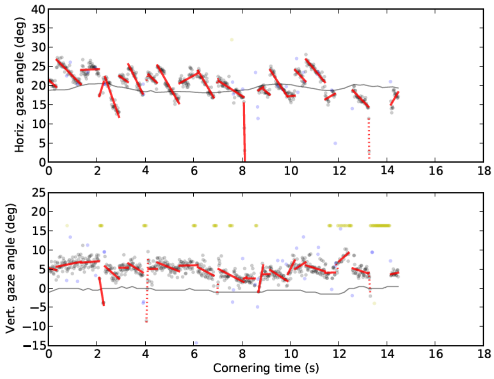

Subject 03, lap 12, noise std h: 0.898, v: 1.077

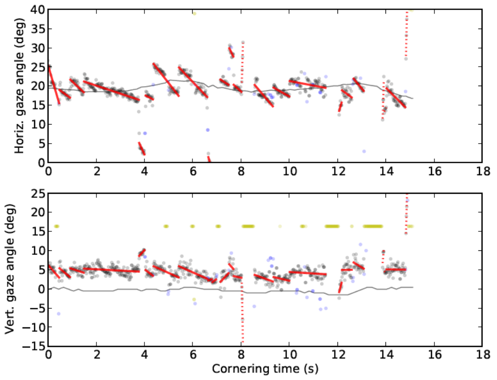

Subject 03, lap 13, noise std h: 0.813, v: 1.093

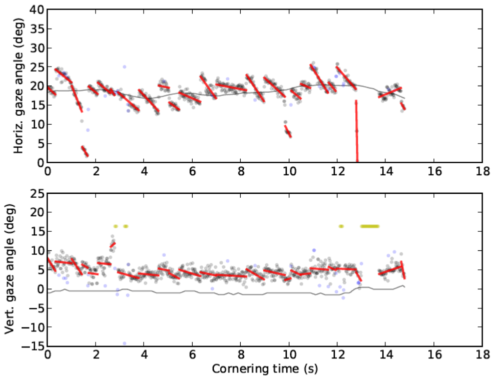

Subject 03, lap 14, noise std h: 0.947, v: 1.335

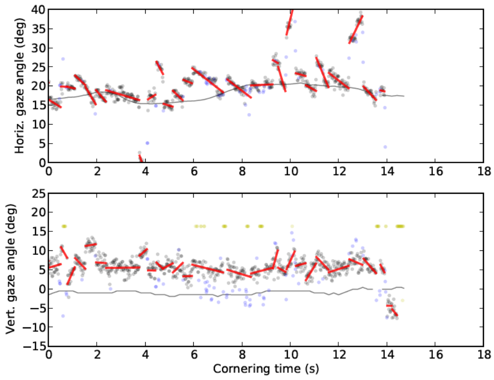

Subject 03, lap 15, noise std h: 0.832, v: 1.175

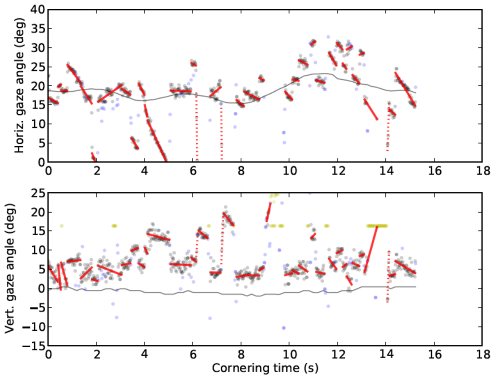

Subject 03, lap 16, noise std h: 0.946, v: 1.297

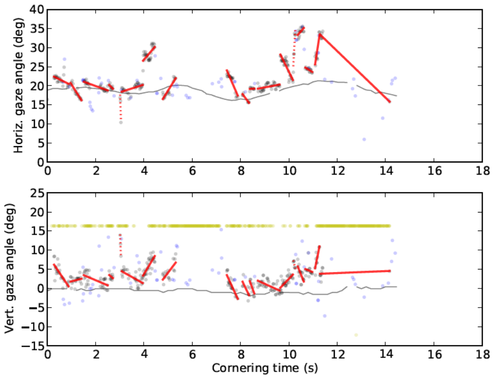

Subject 05, lap 1, noise std h: 0.991, v: 1.230

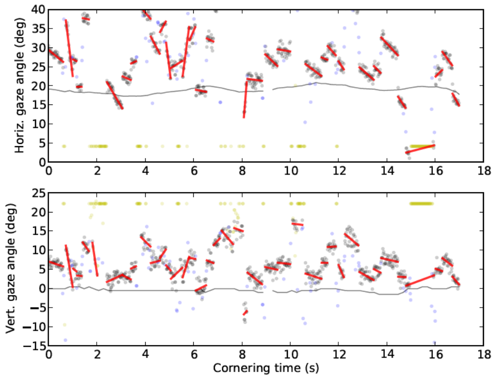

Subject 05, lap 2, noise std h: 1.041, v: 1.181

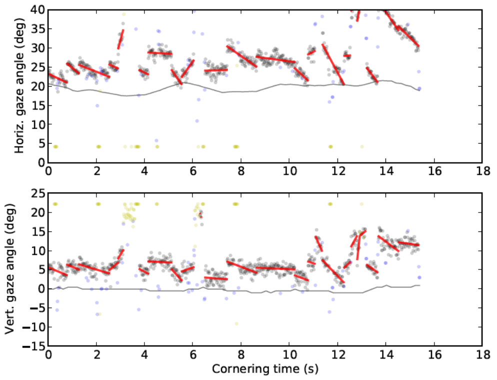

Subject 05, lap 3, noise std h: 0.999, v: 1.179

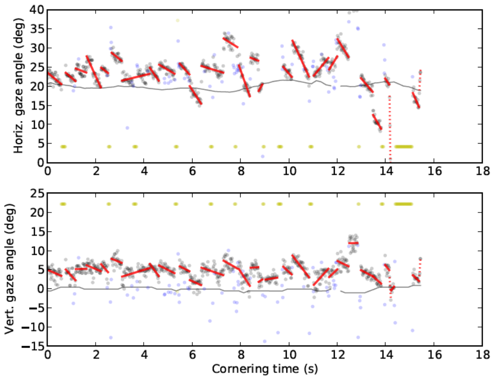

Subject 05, lap 4, noise std h: 0.893, v: 1.054

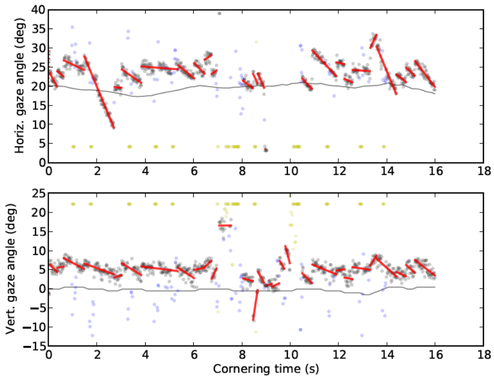

Subject 05, lap 5, noise std h: 0.892, v: 0.972

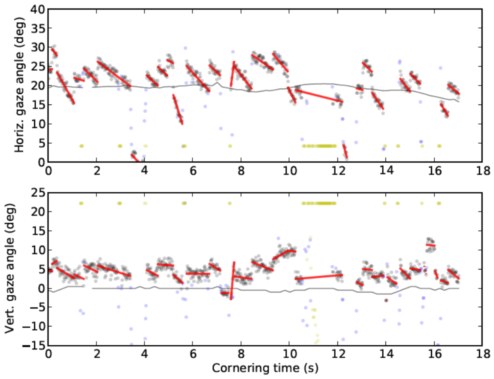

Subject 05, lap 6, noise std h: 0.807, v: 1.111

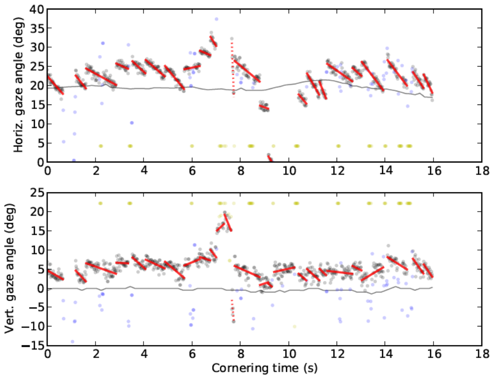

Subject 05, lap 7, noise std h: 0.916, v: 1.042

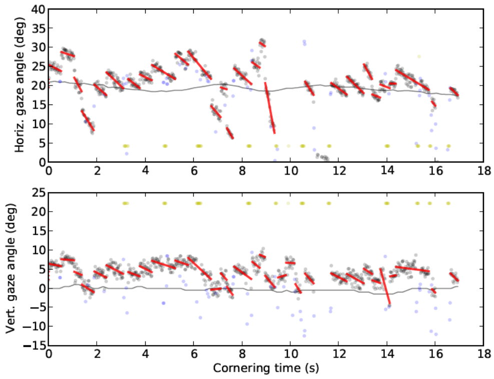

Subject 05, lap 8, noise std h: 0.864, v: 1.050

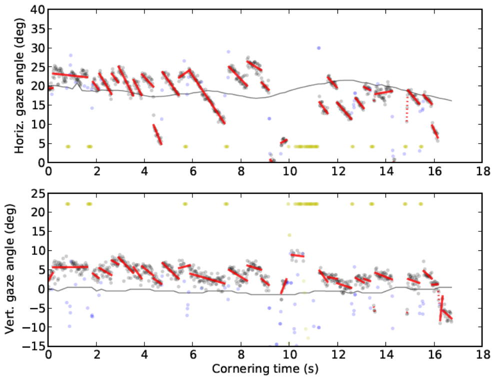

Subject 05, lap 9, noise std h: 0.863, v: 0.968

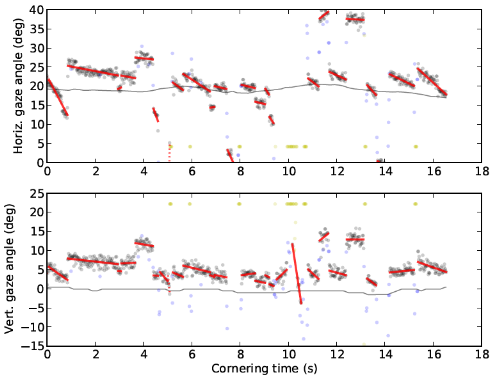

Subject 05, lap 10, noise std h: 0.850, v: 0.963

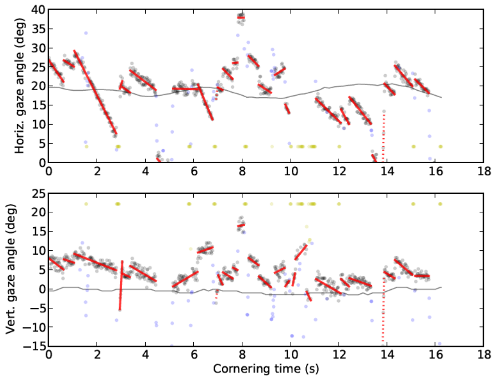

Subject 05, lap 11, noise std h: 0.847, v: 1.069

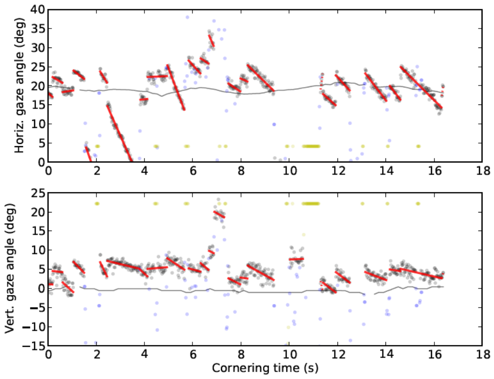

Subject 05, lap 12, noise std h: 0.756, v: 1.044

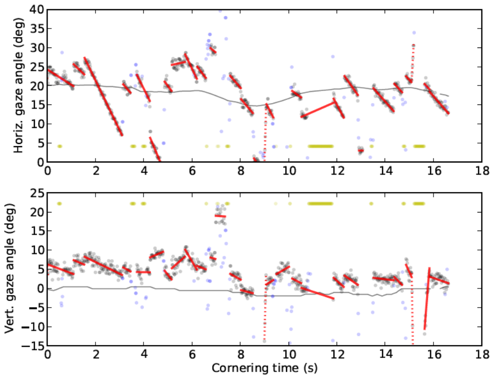

Subject 05, lap 13, noise std h: 0.848, v: 1.074

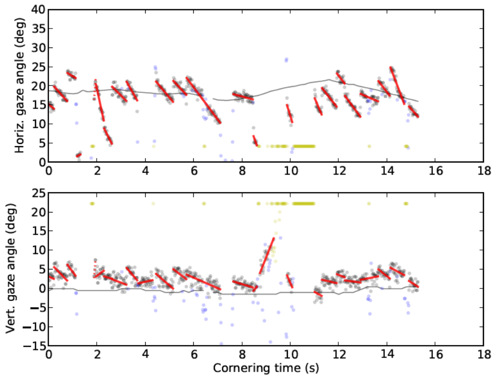

Subject 05, lap 14, noise std h: 0.814, v: 1.058

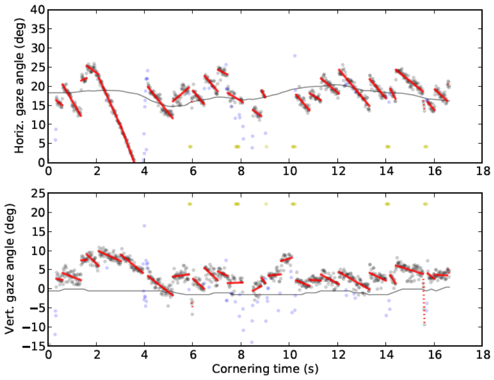

Subject 05, lap 15, noise std h: 0.836, v: 0.917

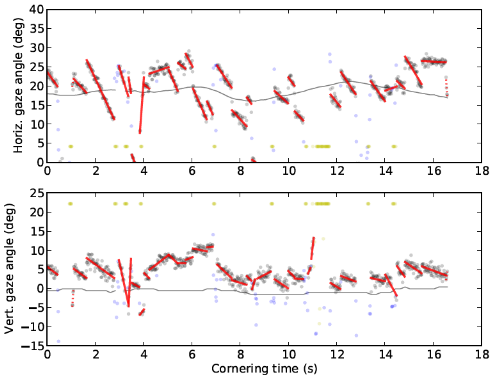

Subject 05, lap 16, noise std h: 0.844, v: 1.002

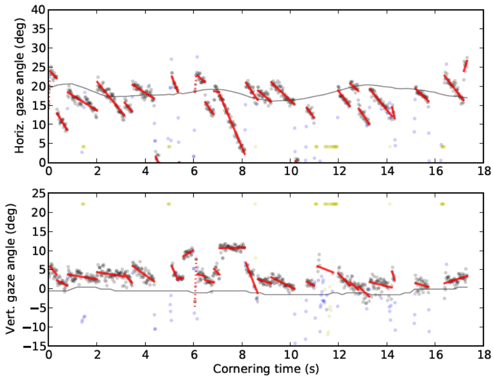

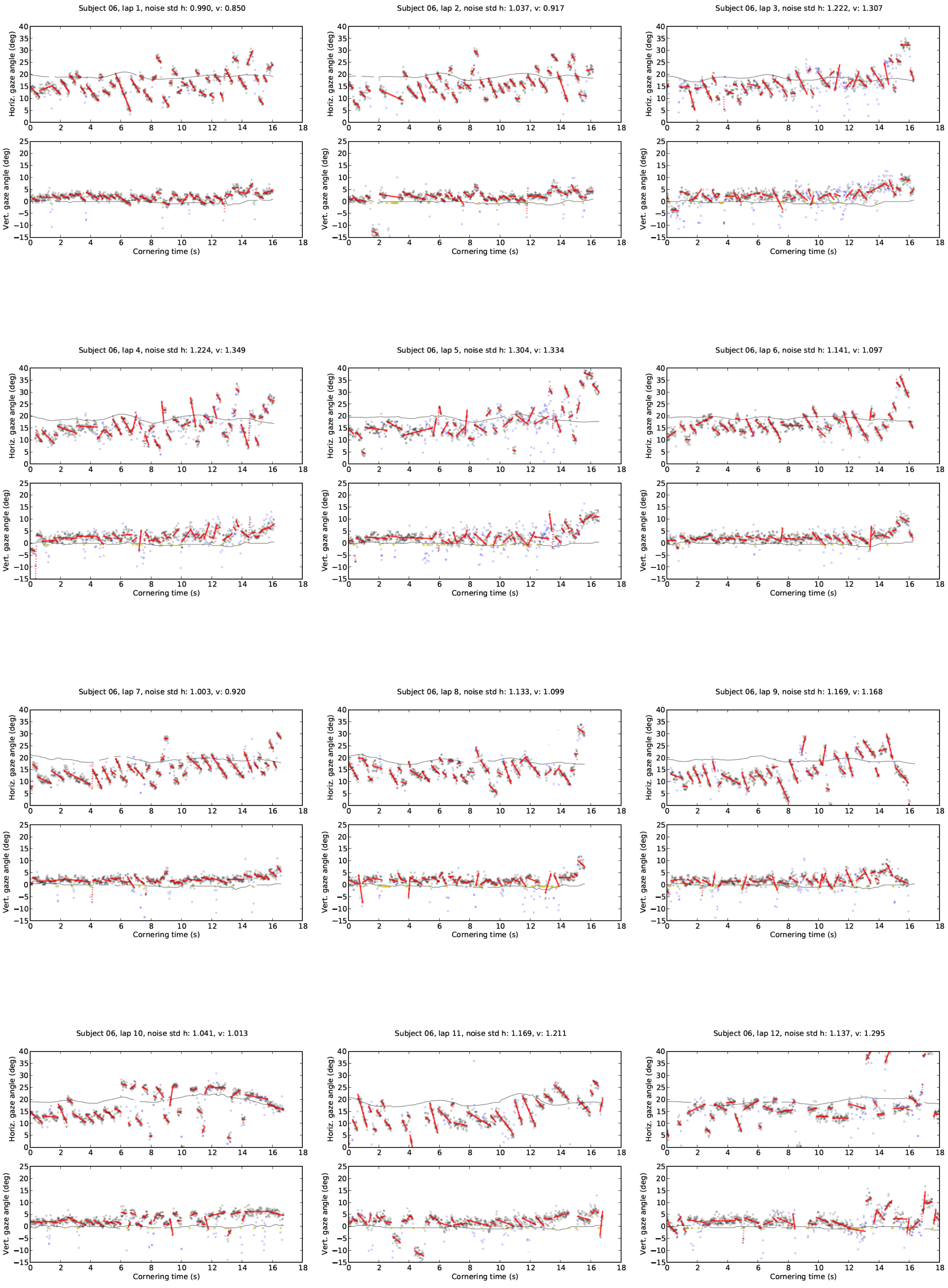
Subject 06, lap 1, noise std h: 0.990, v: 0.850
Subject 06, lap 2, noise std h: 1.037, v: 0.917
Subject 06, lap 3, noise std h: 1.222, v: 1.307
Subject 06, lap 4, noise std h: 1.224, v: 1.349
Subject 06, lap 5, noise std h: 1.304, v: 1.334
Subject 06, lap 6, noise std h: 1.141, v: 1.097
Subject 06, lap 7, noise std h: 1.003, v: 0.920
Subject 06, lap 8, noise std h: 1.133, v: 1.099
Subject 06, lap 9, noise std h: 1.169, v: 1.168
Subject 06, lap 10, noise std h: 1.041, v: 1.013
Subject 06, lap 11, noise std h: 1.169, v: 1.211
Subject 06, lap 12, noise std h: 1.137, v: 1.295
Horiz. gaze angle (deg)
Vert. gaze angle (deg)
Cornering time (s)

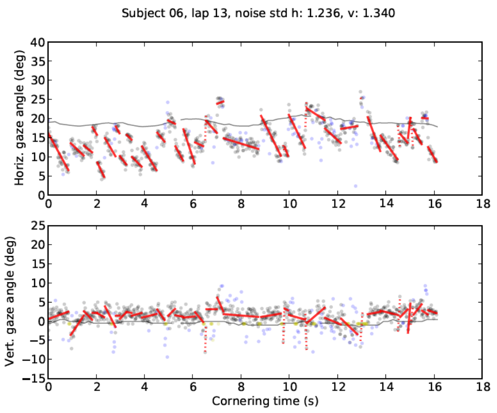
Subject 06, lap 13, noise std h: 1.236, v: 1.340
Horiz. gaze angle (deg)
Vert. gaze angle (deg)
Cornering time (s)

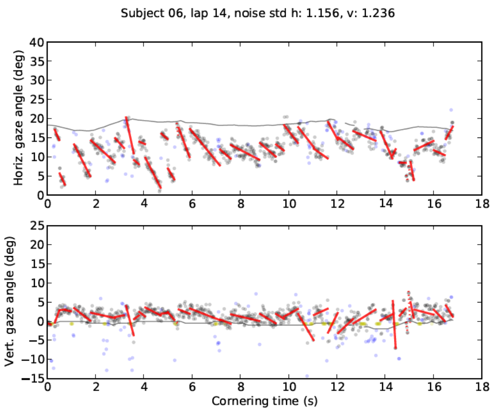
Subject 06, lap 14, noise std h: 1.156, v: 1.236
Horiz. gaze angle (deg)
Vert. gaze angle (deg)
Cornering time (s)

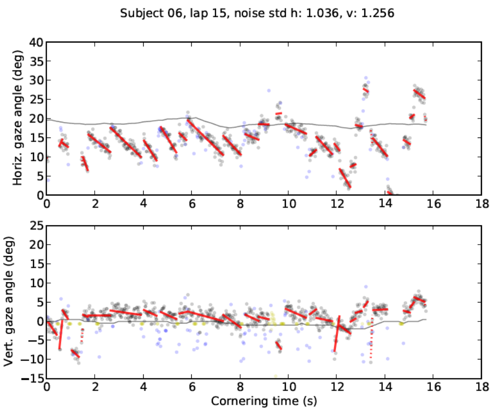
Subject 06, lap 15, noise std h: 1.036, v: 1.256
Horiz. gaze angle (deg)
Vert. gaze angle (deg)
Cornering time (s)

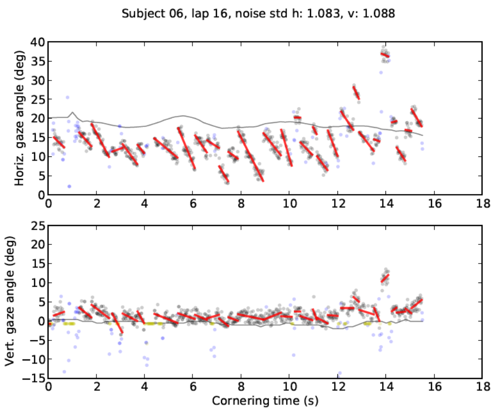
Subject 06, lap 16, noise std h: 1.083, v: 1.088
Horiz. gaze angle (deg)
Vert. gaze angle (deg)
Cornering time (s)

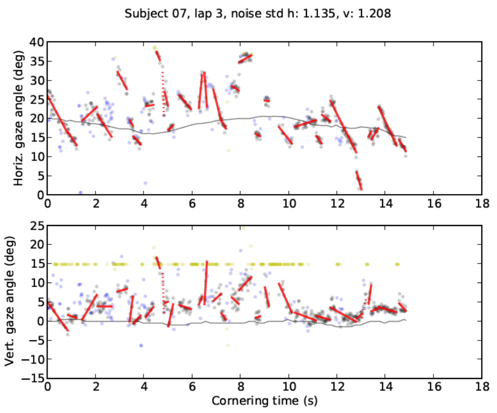
Subject 07, lap 3, noise std h: 1.135, v: 1.208
Horiz. gaze angle (deg)
Vert. gaze angle (deg)
Cornering time (s)

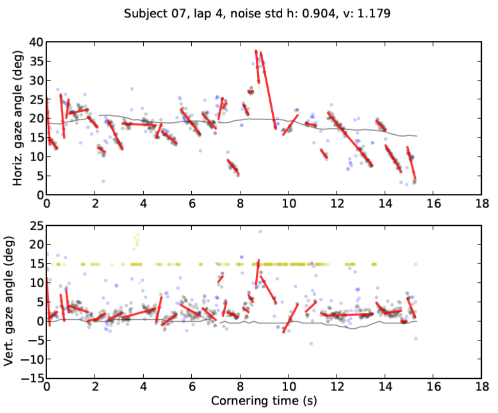
Subject 07, lap 4, noise std h: 0.904, v: 1.179
Horiz. gaze angle (deg)
Vert. gaze angle (deg)
Cornering time (s)

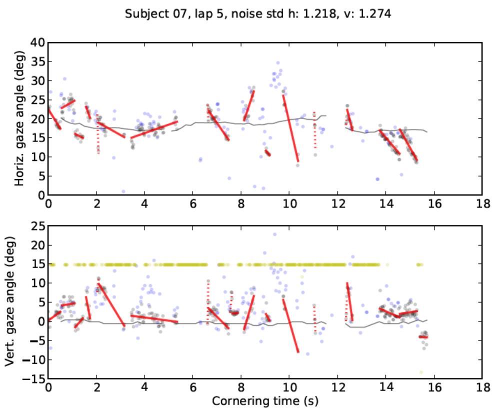
Subject 07, lap 5, noise std h: 1.218, v: 1.274
Horiz. gaze angle (deg)
Vert. gaze angle (deg)
Cornering time (s)

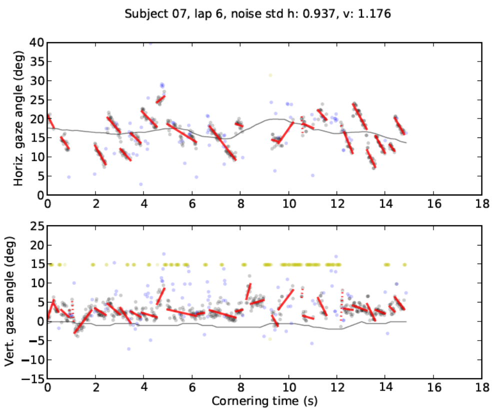
Subject 07, lap 6, noise std h: 0.937, v: 1.176
Horiz. gaze angle (deg)
Vert. gaze angle (deg)
Cornering time (s)

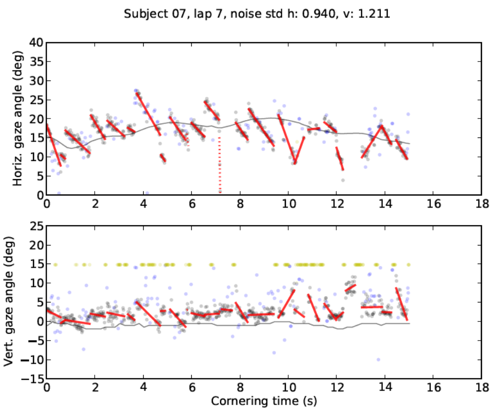
Subject 07, lap 7, noise std h: 0.940, v: 1.211
Horiz. gaze angle (deg)
Vert. gaze angle (deg)
Cornering time (s)

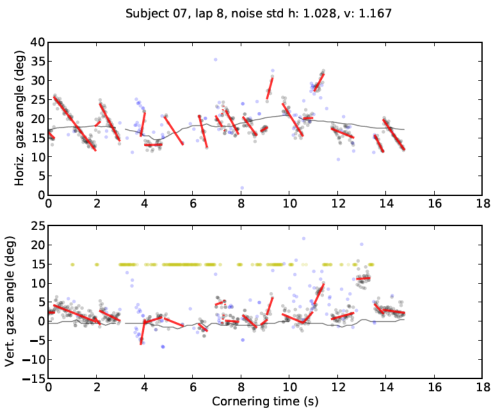
Subject 07, lap 8, noise std h: 1.028, v: 1.167
Horiz. gaze angle (deg)
Vert. gaze angle (deg)
Cornering time (s)

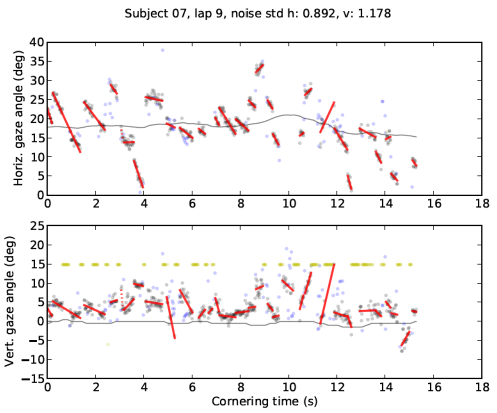
Subject 07, lap 9, noise std h: 0.892, v: 1.178
Horiz. gaze angle (deg)
Vert. gaze angle (deg)
Cornering time (s)

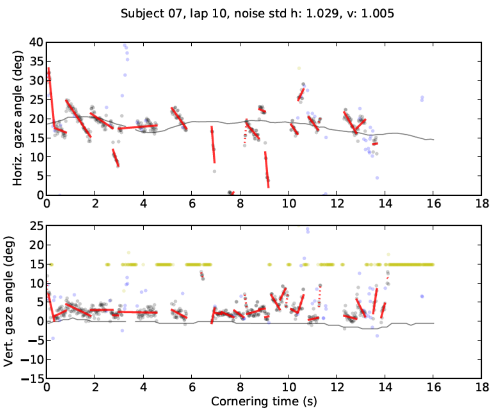
Subject 07, lap 10, noise std h: 1.029, v: 1.005
Horiz. gaze angle (deg)
Vert. gaze angle (deg)
Cornering time (s)

Subject 07, lap 11, noise std h: 0.848, v: 1.159

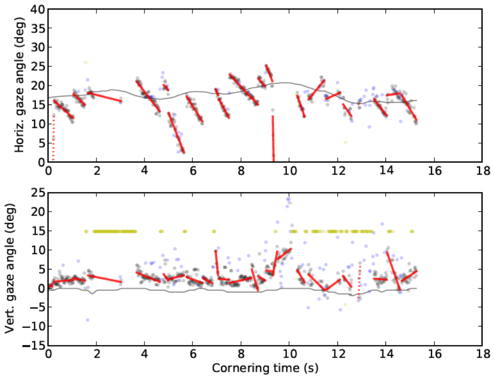

Subject 07, lap 12, noise std h: 0.772, v: 0.886

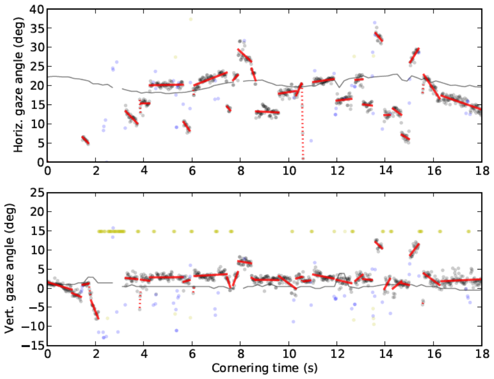

Subject 07, lap 13, noise std h: 0.944, v: 0.995

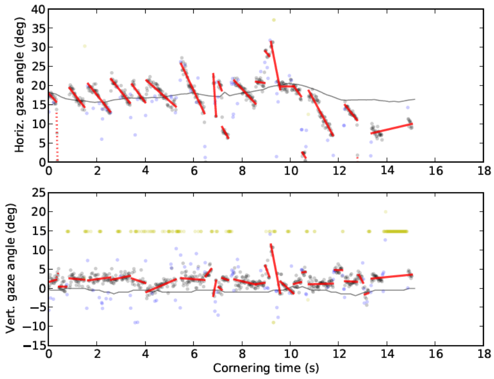

Subject 07, lap 14, noise std h: 0.823, v: 0.989

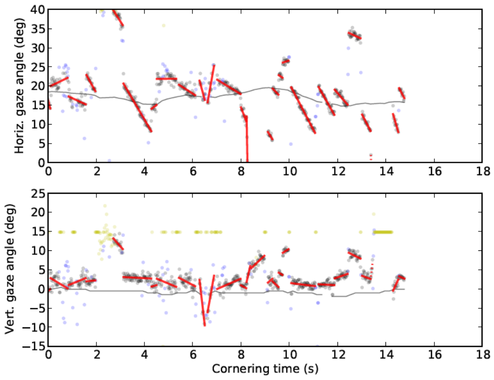

Subject 07, lap 15, noise std h: 0.934, v: 1.079

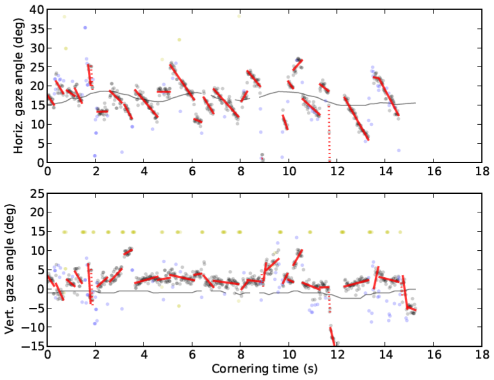

Subject 07, lap 16, noise std h: 0.980, v: 1.256

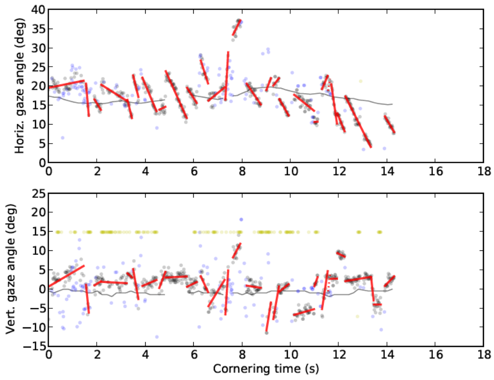

Subject 10, lap 1, noise std h: 0.816, v: 0.931

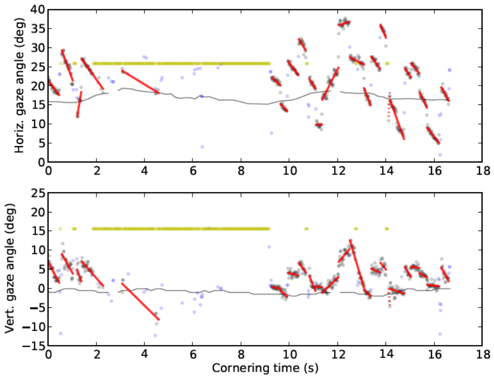

Subject 10, lap 2, noise std h: 0.787, v: 0.926

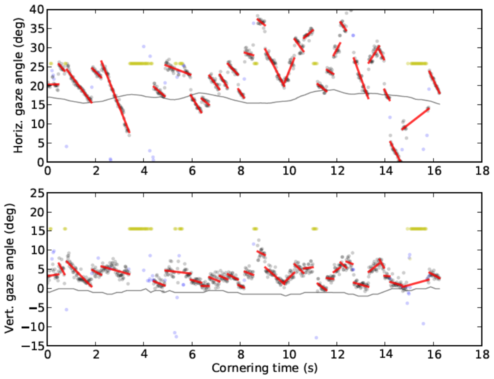

Subject 10, lap 3, noise std h: 0.750, v: 0.829

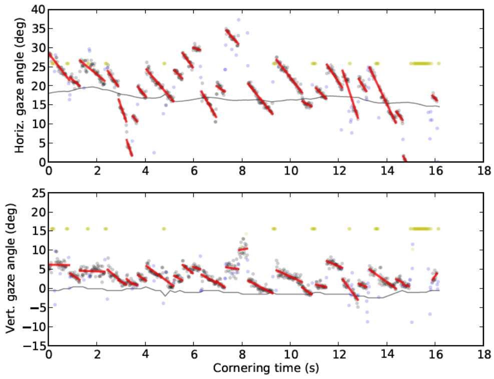

Subject 10, lap 4, noise std h: 0.715, v: 0.767

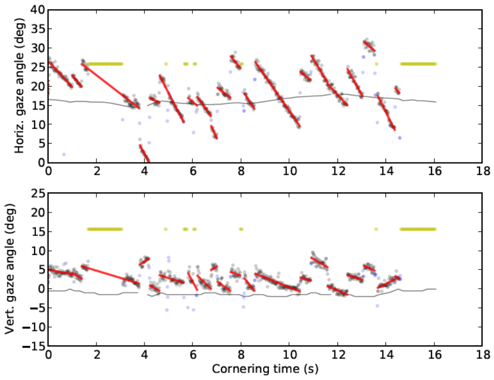

Subject 10, lap 5, noise std h: 0.830, v: 0.764

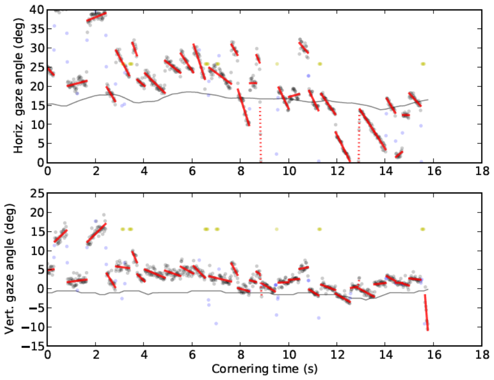

Subject 10, lap 6, noise std h: 0.740, v: 0.874

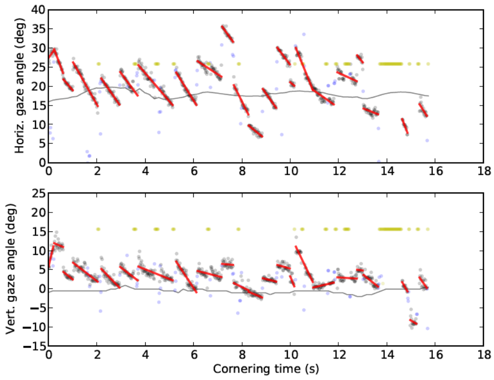

Subject 10, lap 7, noise std h: 0.711, v: 0.685

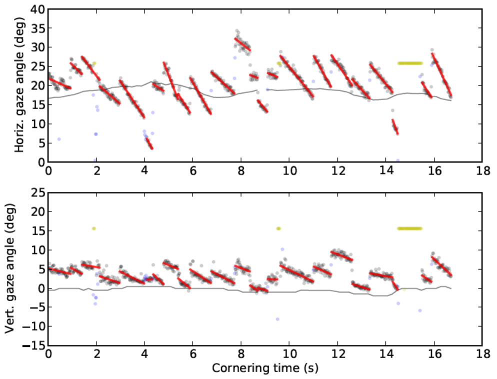

Subject 10, lap 8, noise std h: 0.731, v: 0.701

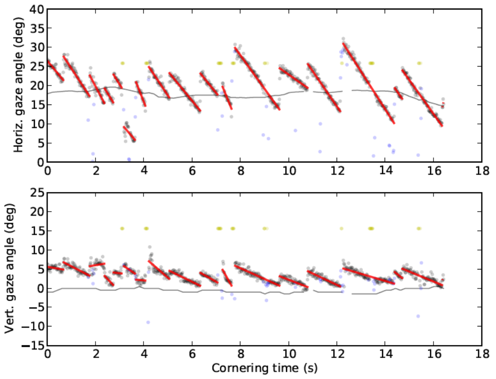

Subject 10, lap 9, noise std h: 0.839, v: 0.860

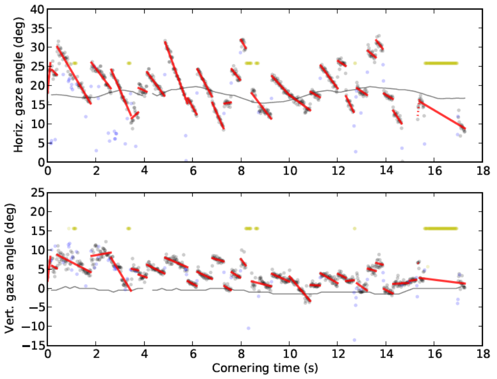

Subject 10, lap 10, noise std h: 0.906, v: 0.770

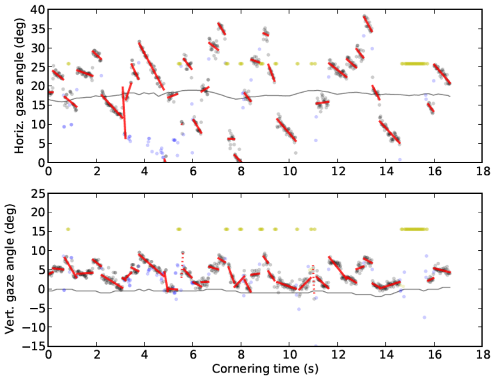

Subject 10, lap 11, noise std h: 0.709, v: 0.642

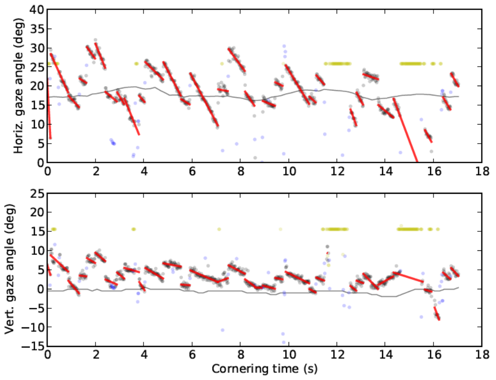

Subject 10, lap 12, noise std h: 0.722, v: 0.716

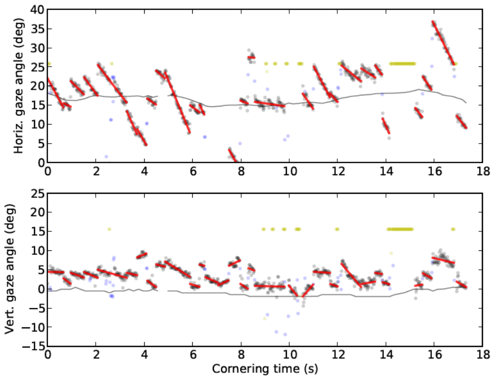

Subject 10, lap 13, noise std h: 0.768, v: 0.852

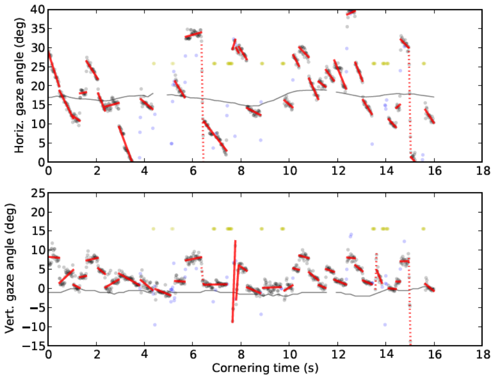

Subject 10, lap 14, noise std h: 0.758, v: 0.679

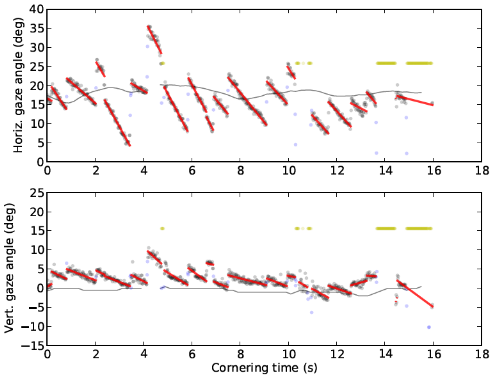

Subject 10, lap 15, noise std h: 0.863, v: 0.769

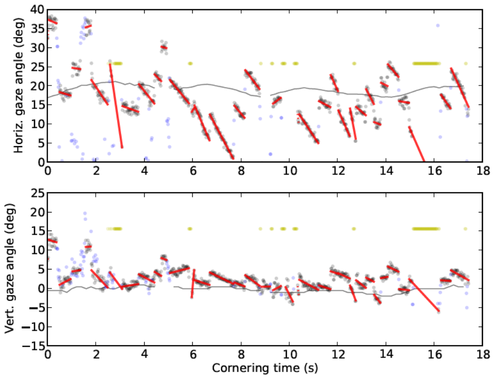

Subject 10, lap 16, noise std h: 0.743, v: 0.673

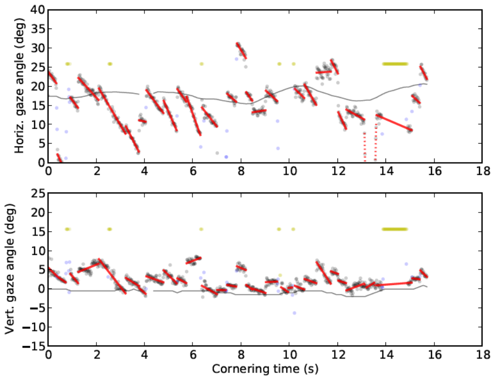

Subject 11, lap 1, noise std h: 0.879, v: 1.056

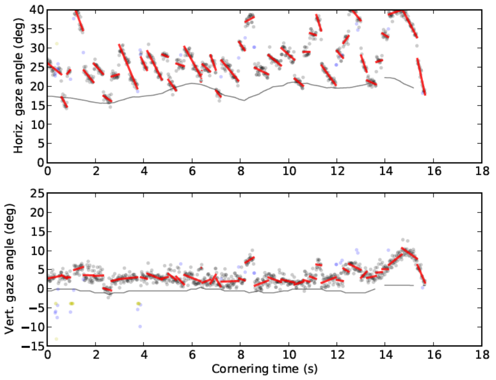

Subject 11, lap 2, noise std h: 0.972, v: 1.231

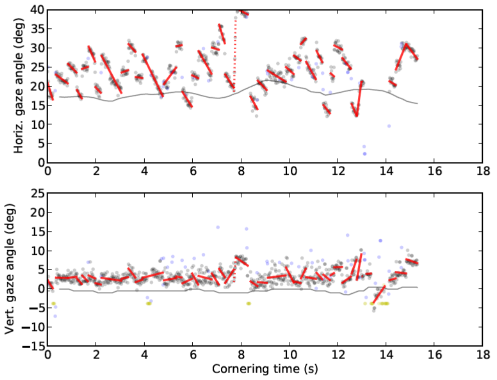

Subject 11, lap 3, noise std h: 0.940, v: 1.099

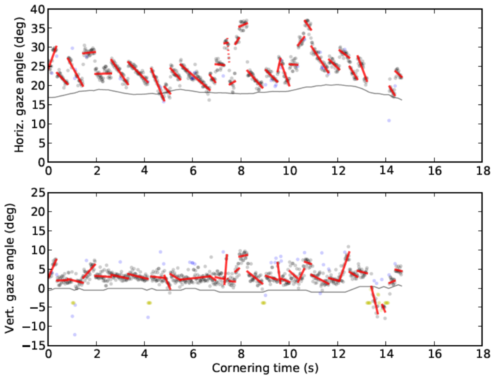

Subject 11, lap 4, noise std h: 0.973, v: 1.160

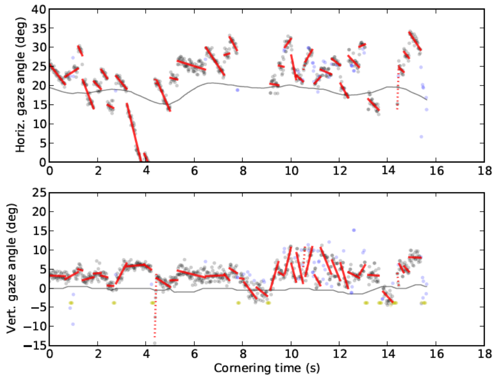

Subject 11, lap 5, noise std h: 0.979, v: 1.146

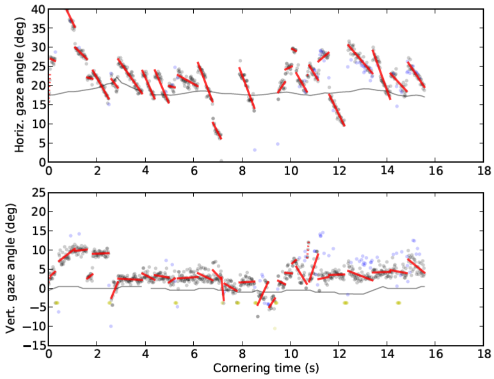

Subject 11, lap 6, noise std h: 0.876, v: 1.071

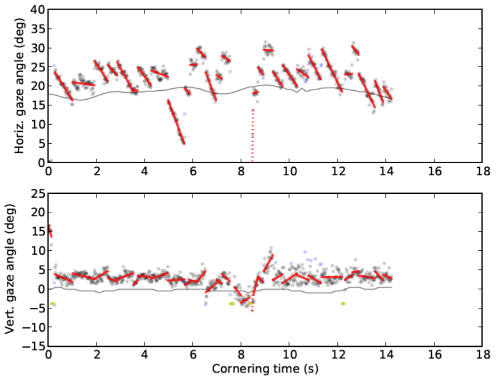

Subject 11, lap 7, noise std h: 0.877, v: 0.974

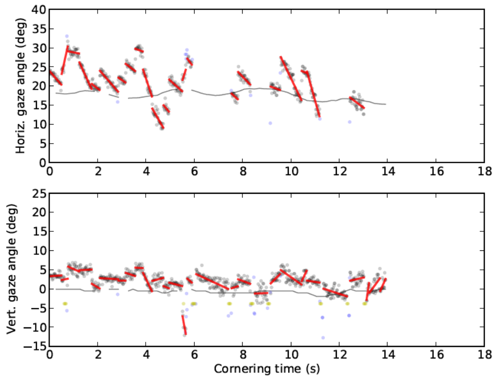

Subject 11, lap 8, noise std h: 0.901, v: 1.133

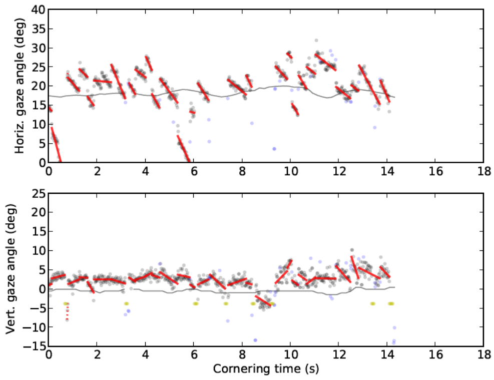

Subject 11, lap 9, noise std h: 0.873, v: 1.190

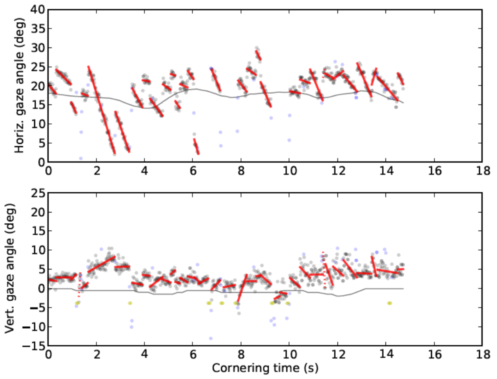

Subject 11, lap 10, noise std h: 0.929, v: 1.057

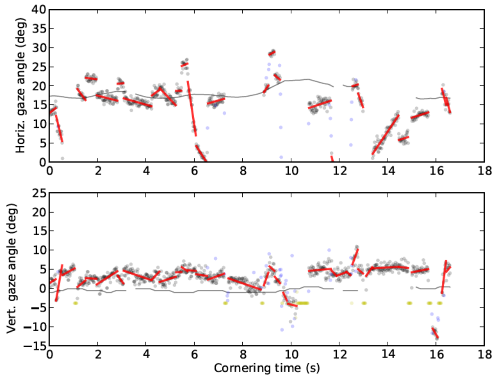

Subject 11, lap 11, noise std h: 0.906, v: 0.910

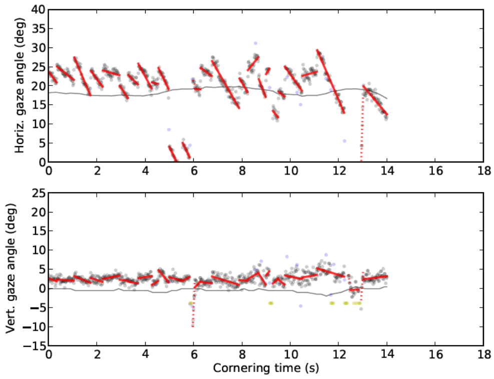

Subject 11, lap 12, noise std h: 0.845, v: 1.133

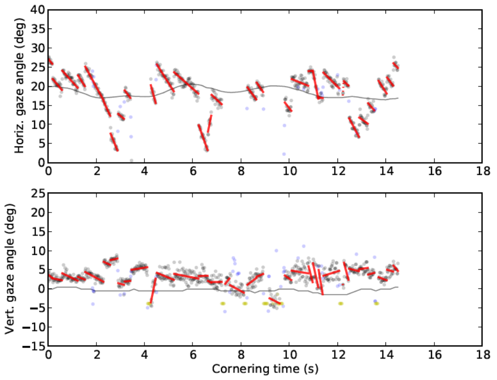

Subject 11, lap 13, noise std h: 0.954, v: 1.097

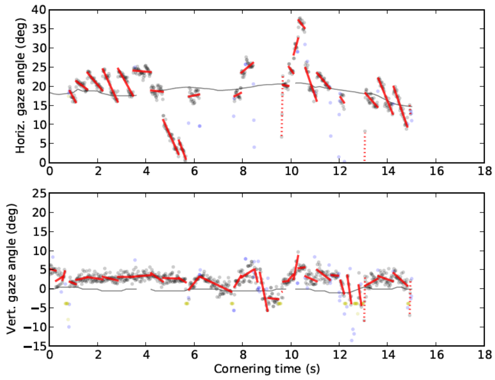

Subject 11, lap 14, noise std h: 0.868, v: 1.085

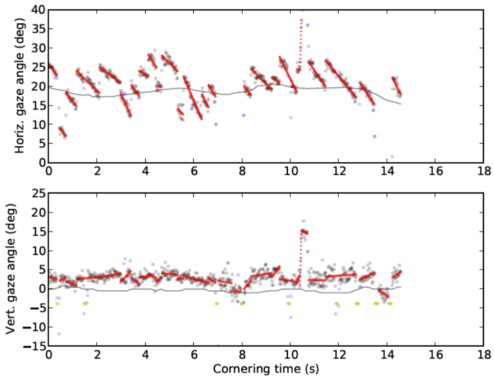

Subject 11, lap 15, noise std h: 0.950, v: 1.118

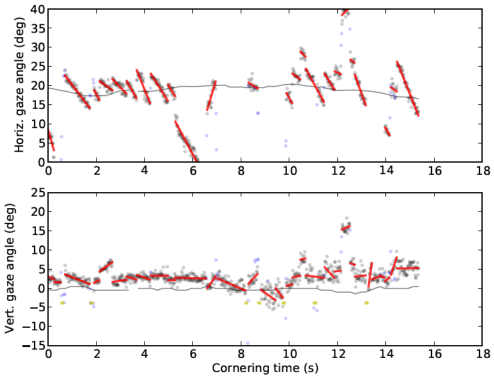

Subject 11, lap 16, noise std h: 0.904, v: 1.243

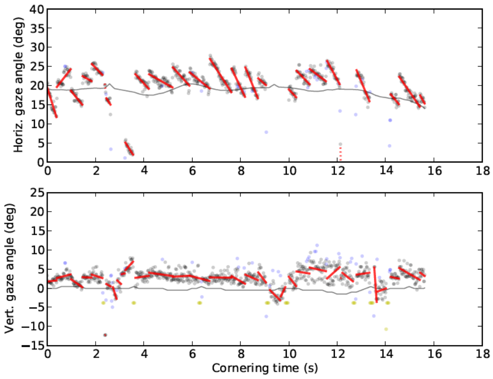

Subject 12, lap 1, noise std h: 0.761, v: 1.297

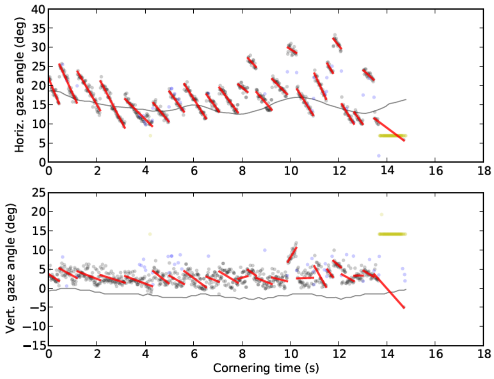

Subject 12, lap 2, noise std h: 0.837, v: 0.940

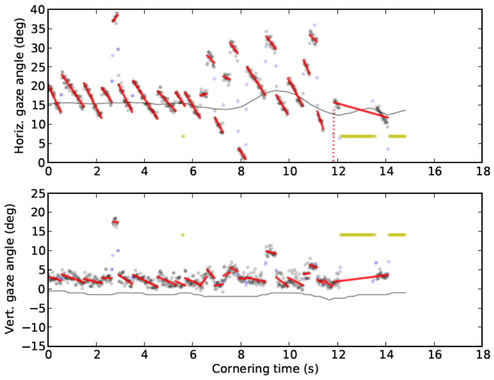

Subject 12, lap 3, noise std h: 0.836, v: 0.821

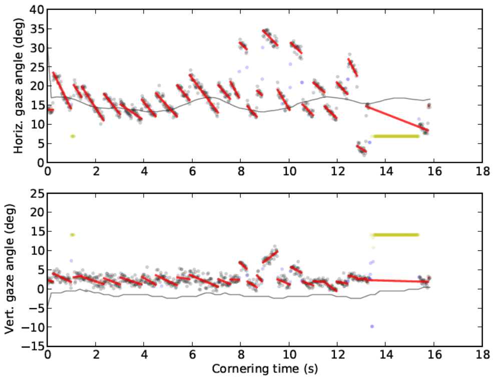

Subject 12, lap 4, noise std h: 0.833, v: 0.800

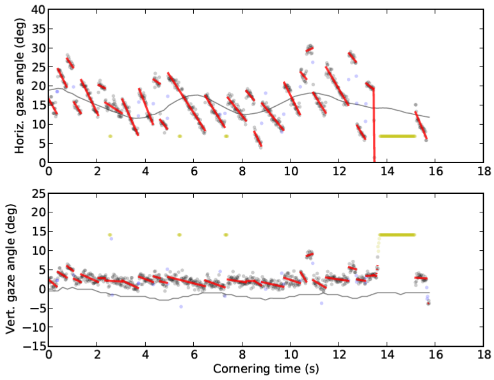

Subject 12, lap 5, noise std h: 0.804, v: 0.786

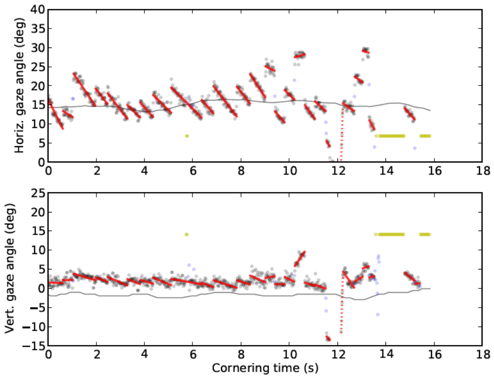

Subject 12, lap 6, noise std h: 0.815, v: 0.944

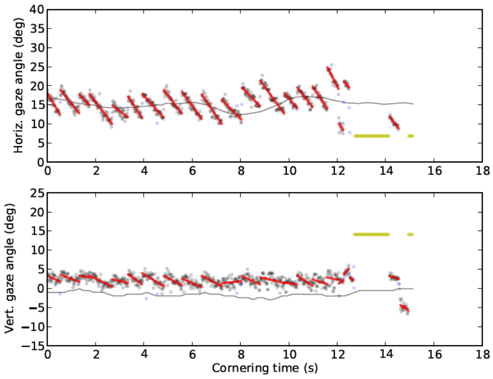

Subject 12, lap 7, noise std h: 0.841, v: 0.894

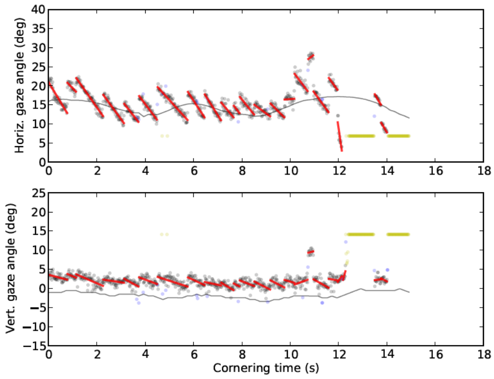

Subject 12, lap 8, noise std h: 0.872, v: 0.829

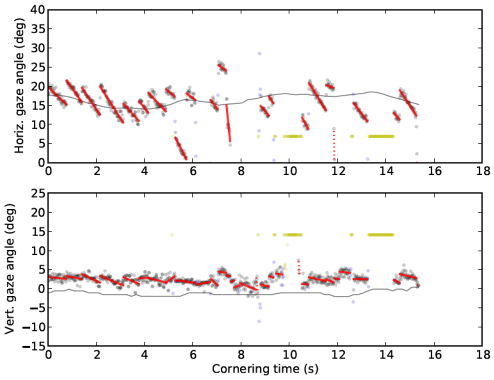

Subject 12, lap 9, noise std h: 0.782, v: 0.865

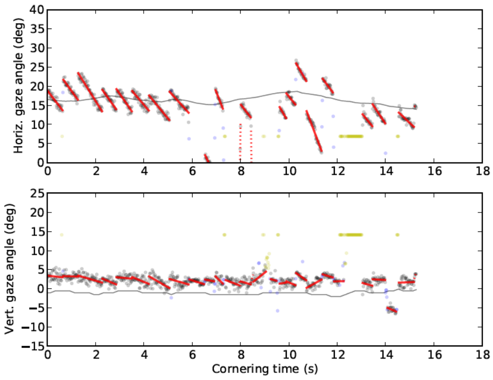

Subject 12, lap 10, noise std h: 0.753, v: 0.841

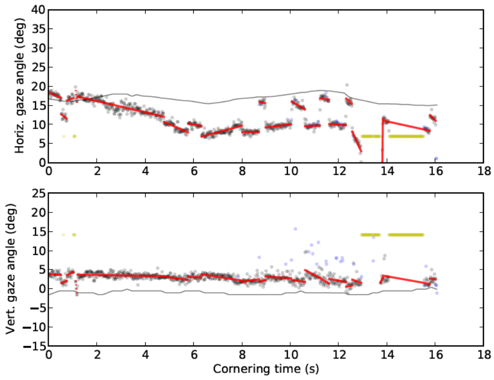

Subject 12, lap 11, noise std h: 0.885, v: 0.849

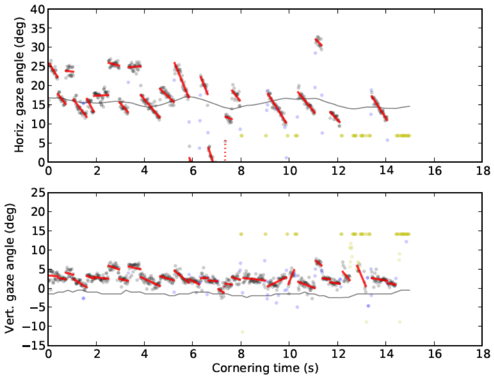

Subject 12, lap 12, noise std h: 0.753, v: 0.750

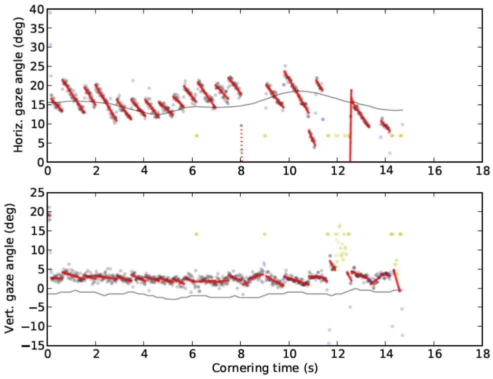

Subject 12, lap 13, noise std h: 0.853, v: 0.847

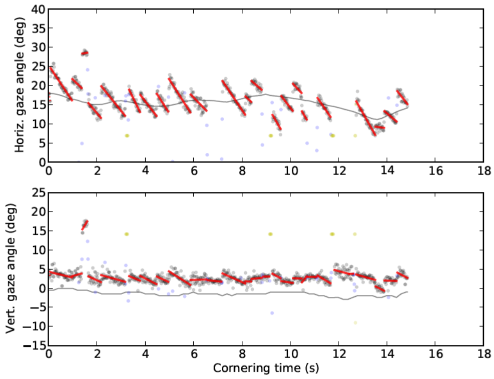

Subject 12, lap 14, noise std h: 0.872, v: 0.723

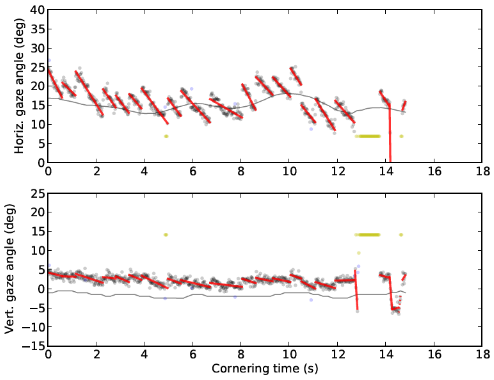

Subject 12, lap 15, noise std h: 0.871, v: 0.830

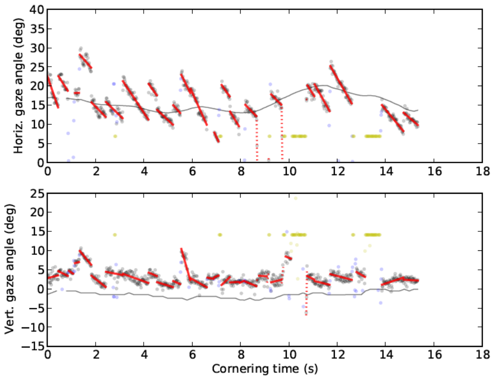

Subject 12, lap 16, noise std h: 0.765, v: 0.741

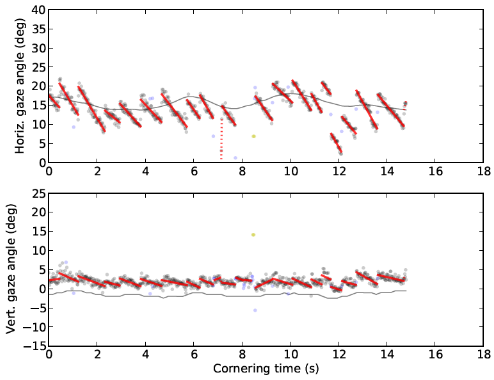

Subject 13, lap 1, noise std h: 1.124, v: 1.151

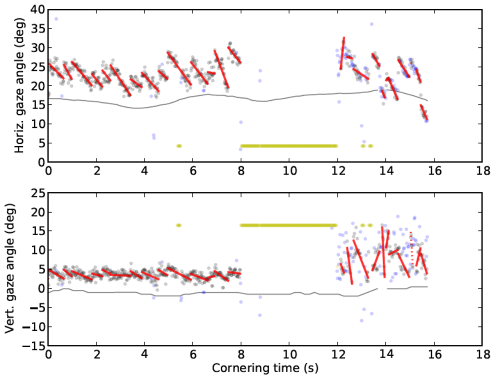

Subject 13, lap 2, noise std h: 1.075, v: 1.351

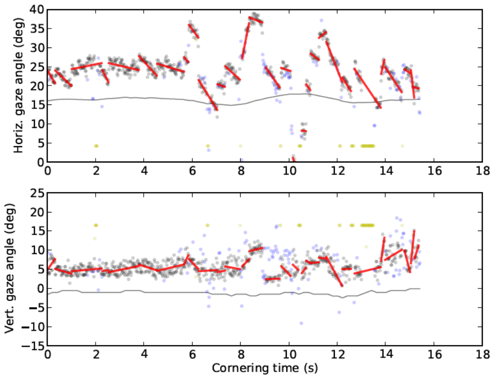

Subject 13, lap 3, noise std h: 0.990, v: 1.545

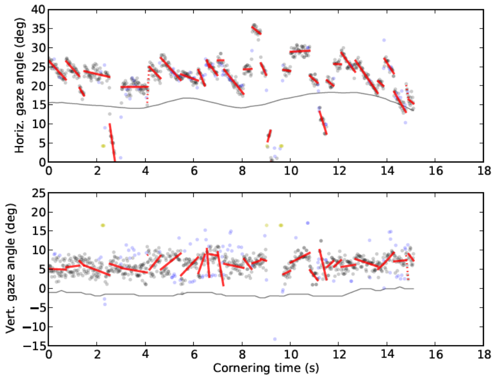

Subject 13, lap 4, noise std h: 1.044, v: 1.489

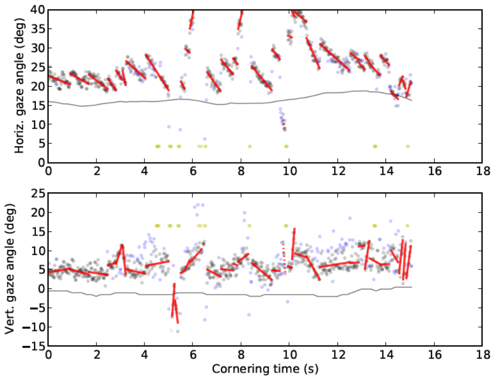

Subject 13, lap 5, noise std h: 1.072, v: 1.302

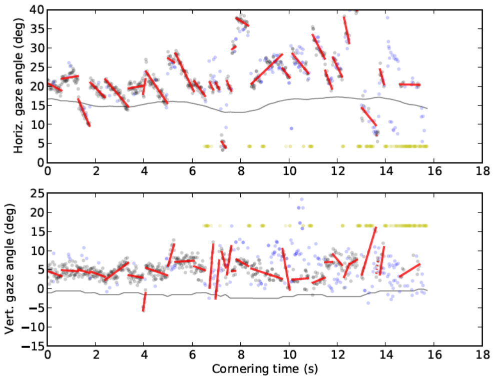

Subject 13, lap 6, noise std h: 1.108, v: 1.442

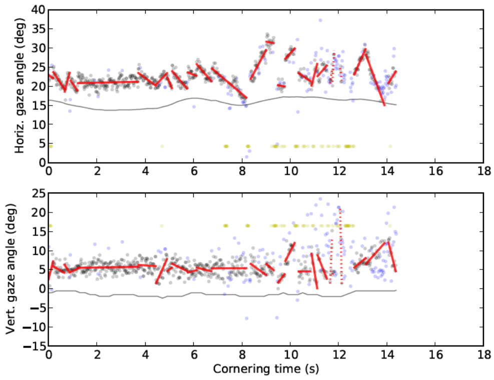

Subject 13, lap 7, noise std h: 1.034, v: 1.484

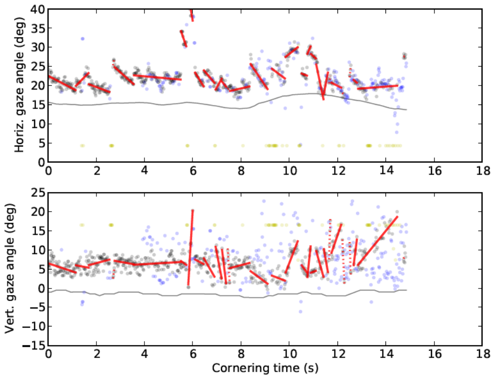

Subject 13, lap 8, noise std h: 1.116, v: 1.429

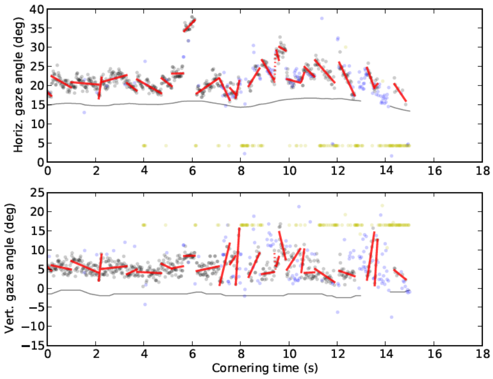

Subject 13, lap 9, noise std h: 1.053, v: 1.581

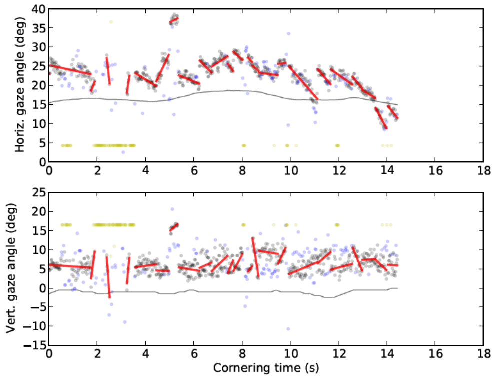

Subject 13, lap 10, noise std h: 1.042, v: 1.462

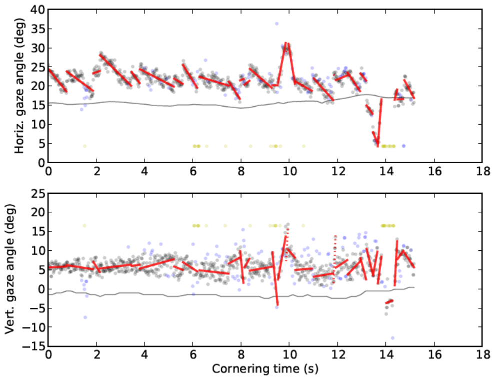

Subject 13, lap 11, noise std h: 1.012, v: 1.215

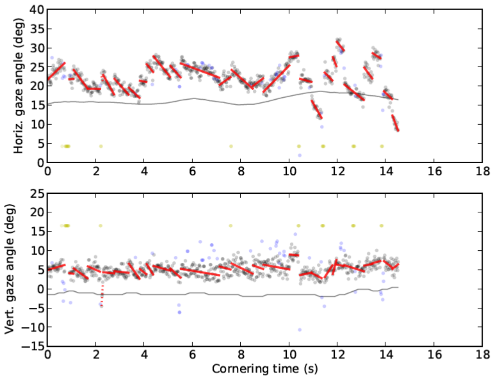

Subject 13, lap 12, noise std h: 1.165, v: 1.397

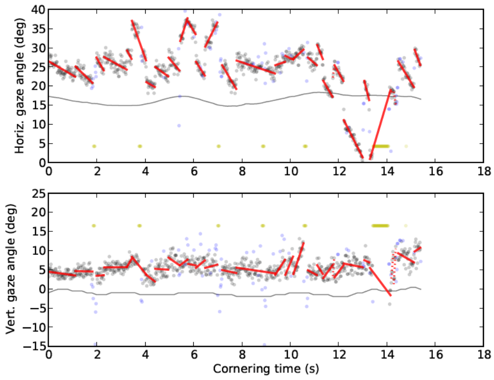

Subject 13, lap 13, noise std h: 1.062, v: 1.522

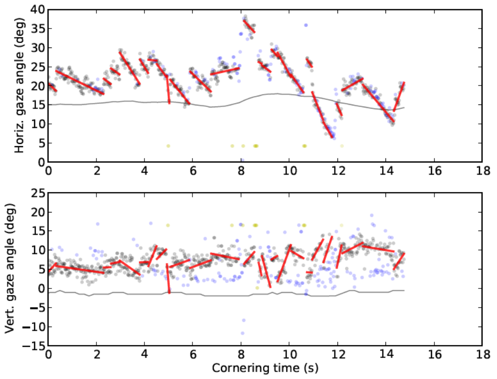

Subject 13, lap 14, noise std h: 1.187, v: 1.475

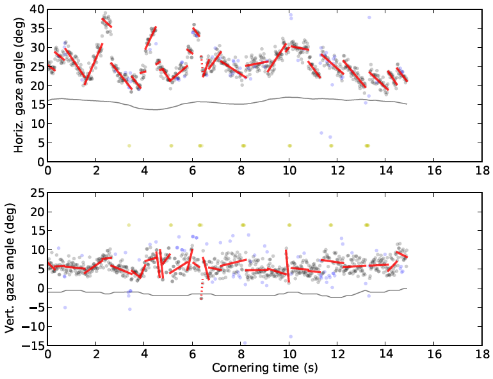

Subject 13, lap 15, noise std h: 1.163, v: 1.492

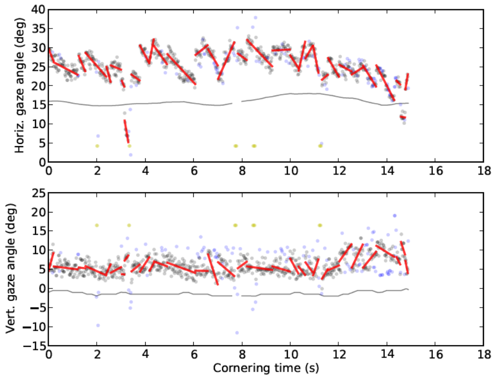

Subject 13, lap 16, noise std h: 1.291, v: 1.230

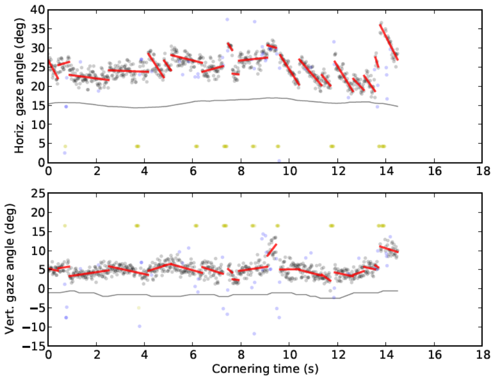

Subject 14, lap 1, noise std h: 1.075, v: 1.187

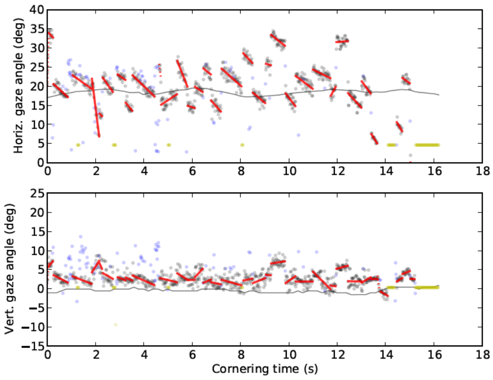

Subject 14, lap 2, noise std h: 1.007, v: 0.994

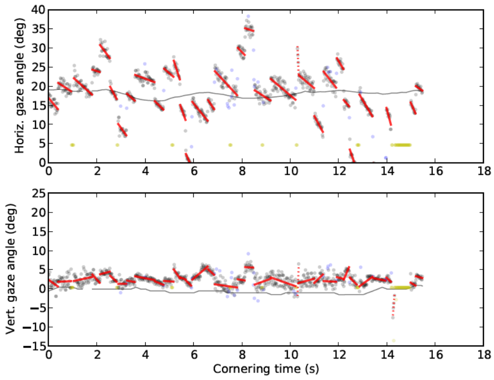

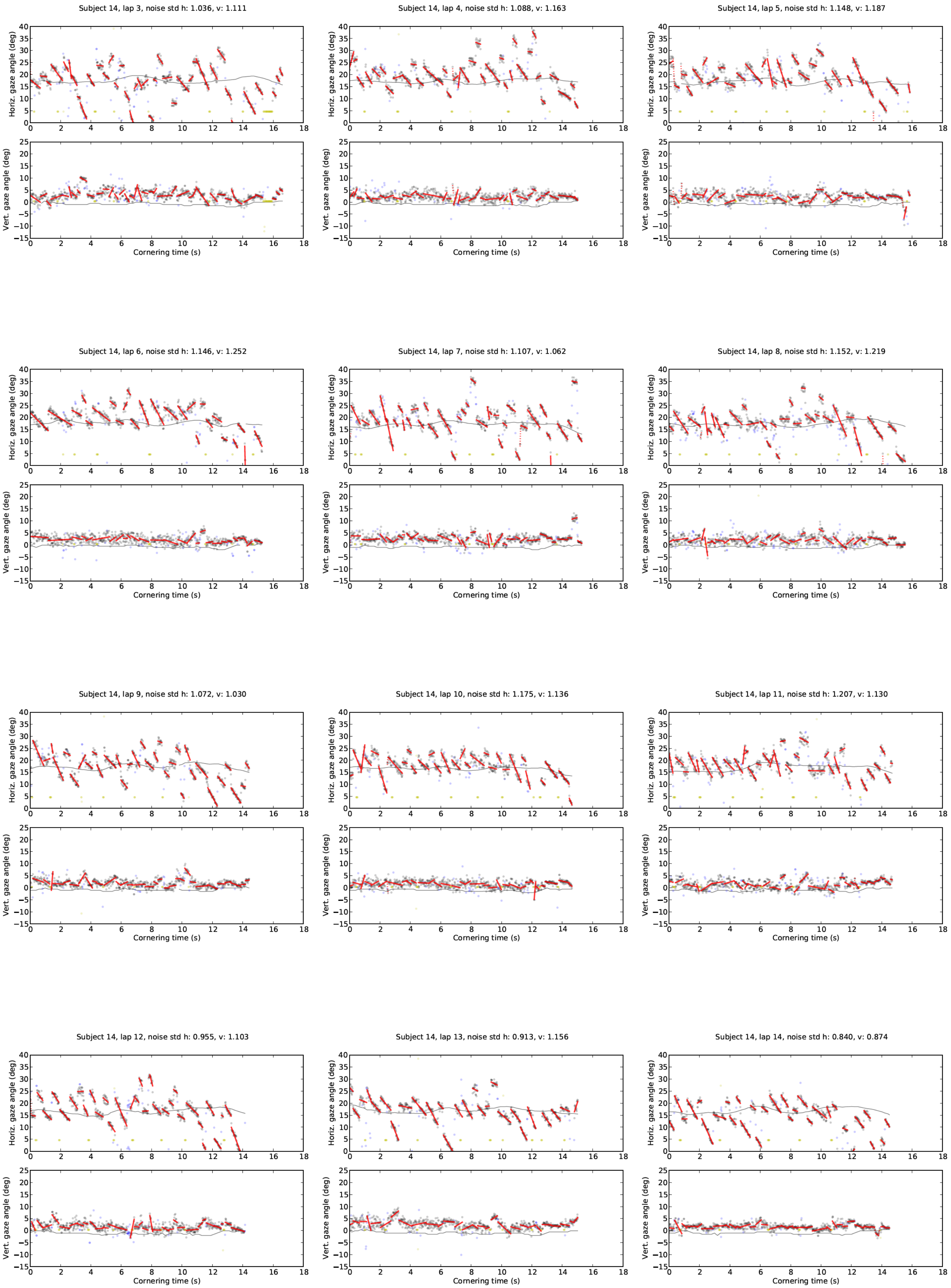

Subject 14, lap 3, noise std h: 1.036, v: 1.111
Subject 14, lap 4, noise std h: 1.088, v: 1.163
Subject 14, lap 5, noise std h: 1.148, v: 1.187
Subject 14, lap 6, noise std h: 1.146, v: 1.252
Subject 14, lap 7, noise std h: 1.107, v: 1.062
Subject 14, lap 8, noise std h: 1.152, v: 1.219
Subject 14, lap 9, noise std h: 1.072, v: 1.030
Subject 14, lap 10, noise std h: 1.175, v: 1.136
Subject 14, lap 11, noise std h: 1.207, v: 1.130
Subject 14, lap 12, noise std h: 0.955, v: 1.103
Subject 14, lap 13, noise std h: 0.913, v: 1.156
Subject 14, lap 14, noise std h: 0.840, v: 0.874
Horiz. gaze angle (deg)
Vert. gaze angle (deg)
Cornering time (s)

Subject 14, lap 15, noise std h: 0.762, v: 0.625

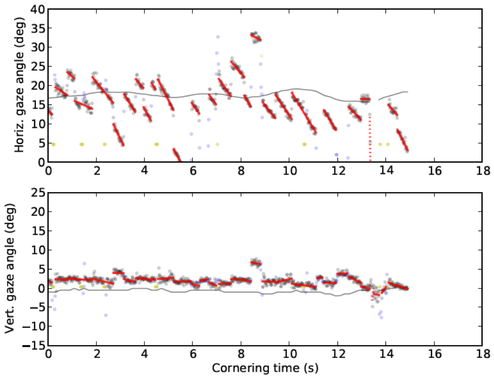

Subject 14, lap 16, noise std h: 0.967, v: 1.122

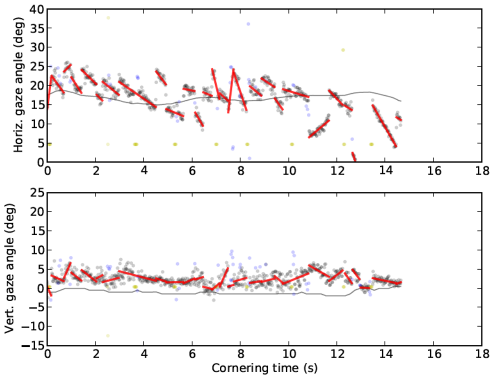

Subject 15, lap 1, noise std h: 0.962, v: 1.022

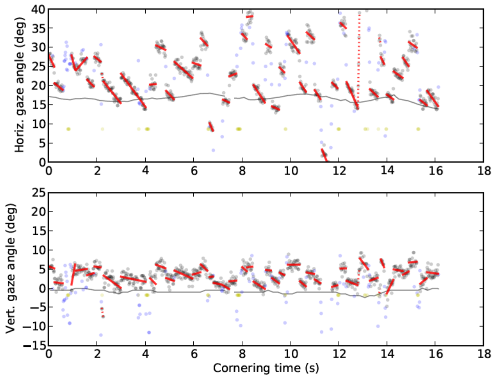

Subject 15, lap 2, noise std h: 1.061, v: 1.112

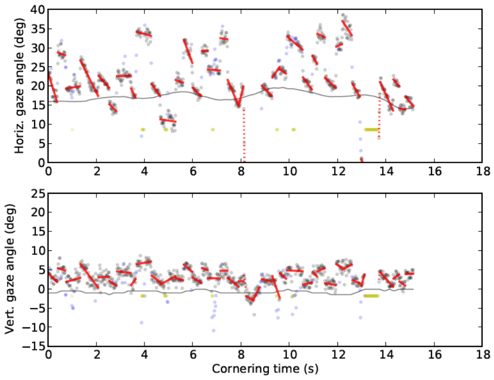

Subject 15, lap 3, noise std h: 0.978, v: 1.050

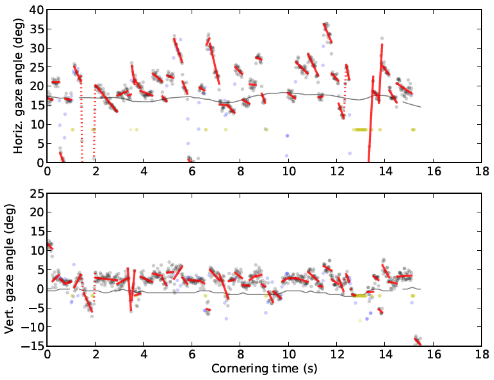

Subject 15, lap 4, noise std h: 0.960, v: 1.104

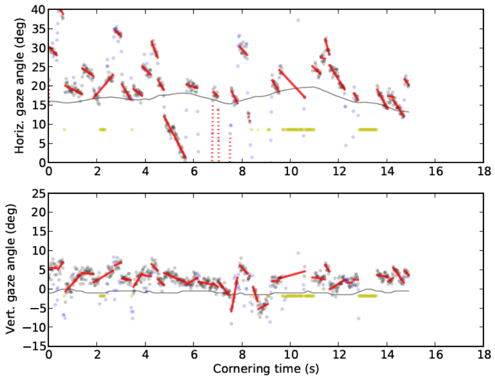

Subject 15, lap 5, noise std h: 1.039, v: 1.095

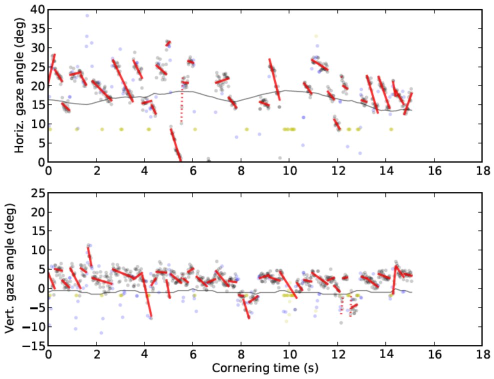

Subject 15, lap 6, noise std h: 1.039, v: 1.037

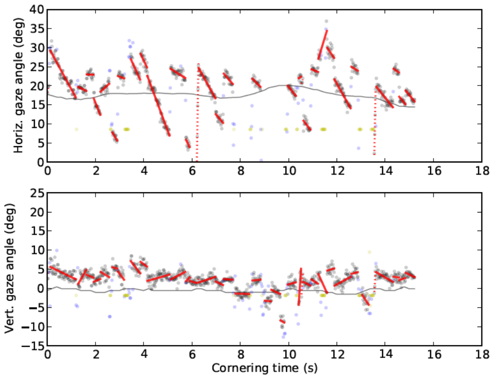

Subject 15, lap 7, noise std h: 1.037, v: 1.098

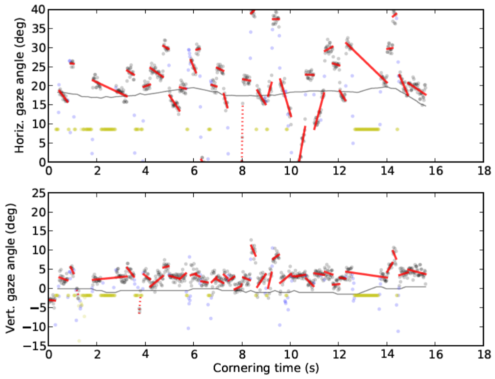

Subject 15, lap 8, noise std h: 1.025, v: 0.994

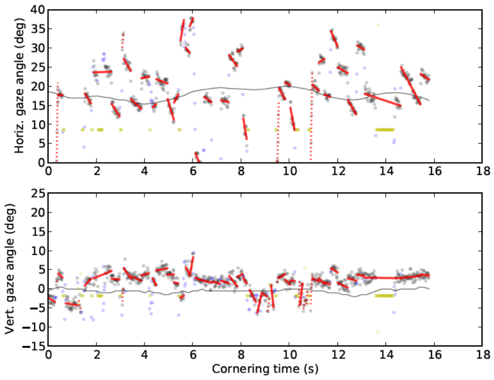

Subject 15, lap 9, noise std h: 1.020, v: 1.059

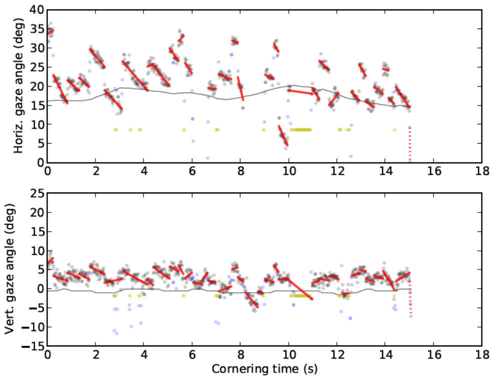

Subject 15, lap 10, noise std h: 1.017, v: 1.114

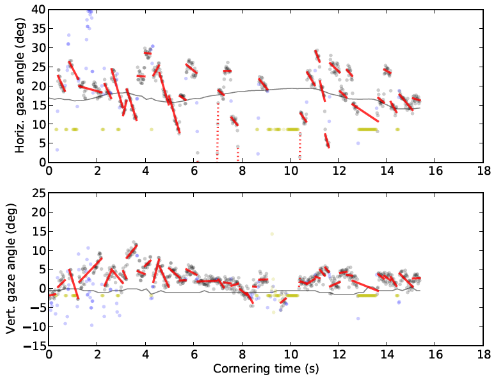

Subject 15, lap 11, noise std h: 0.945, v: 1.089

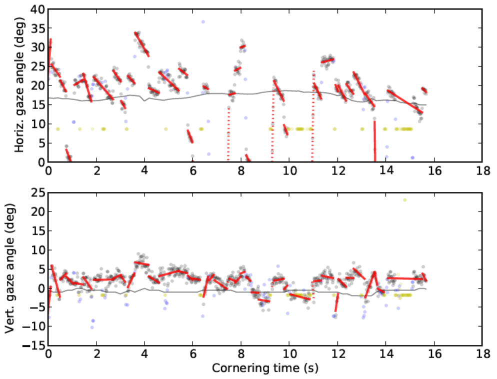

Subject 15, lap 12, noise std h: 0.972, v: 0.988

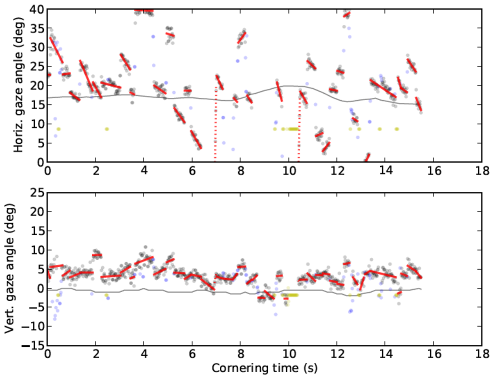

Subject 15, lap 13, noise std h: 1.017, v: 1.051

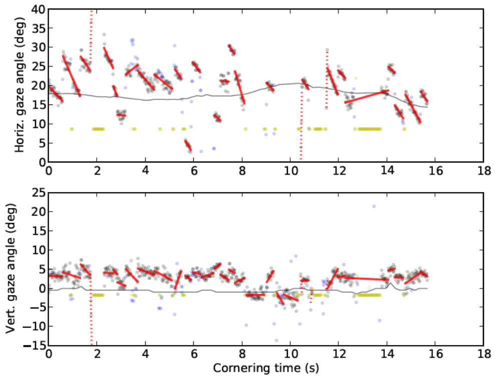

Subject 15, lap 14, noise std h: 0.991, v: 1.040

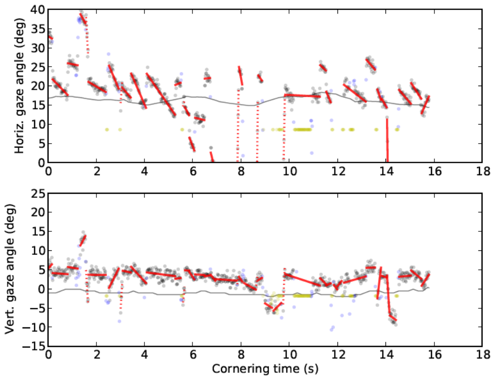

Subject 15, lap 15, noise std h: 0.966, v: 1.024

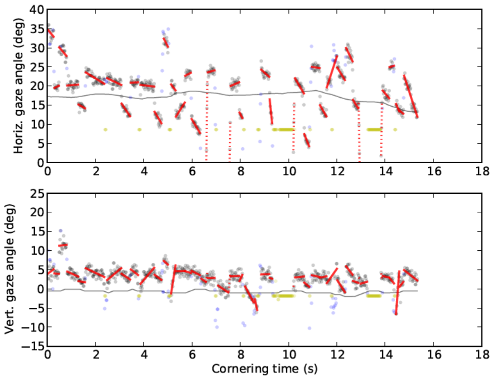

Subject 15, lap 16, noise std h: 0.941, v: 1.085

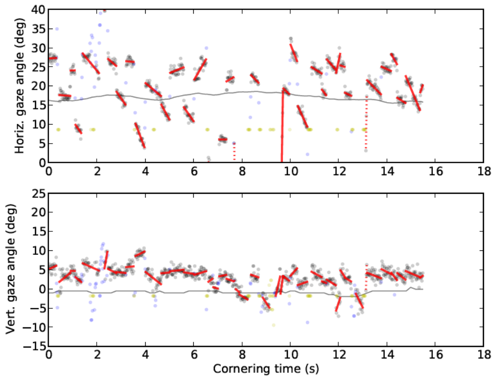

Subject 16, lap 1, noise std h: 0.989, v: 1.183

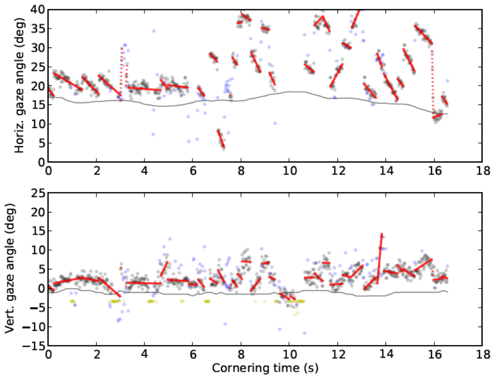

Subject 16, lap 2, noise std h: 1.025, v: 1.186

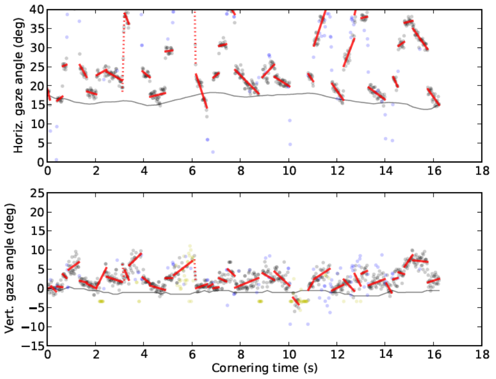

Subject 16, lap 3, noise std h: 1.131, v: 1.362

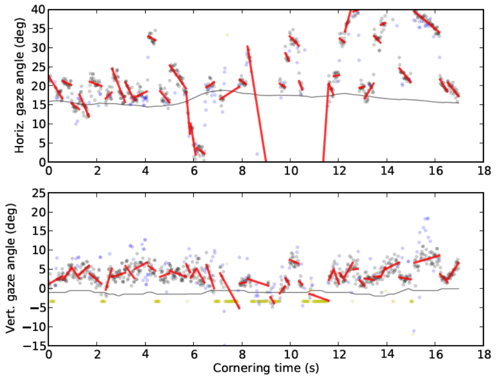

Subject 16, lap 4, noise std h: 1.022, v: 1.205

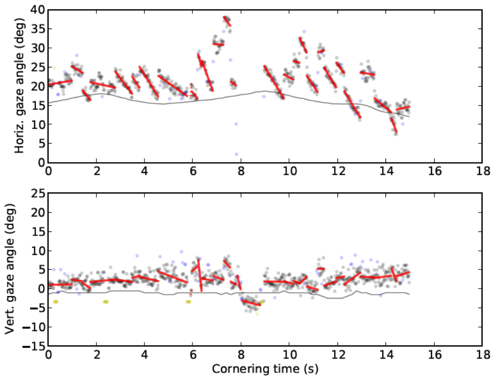

Subject 16, lap 5, noise std h: 0.955, v: 1.289

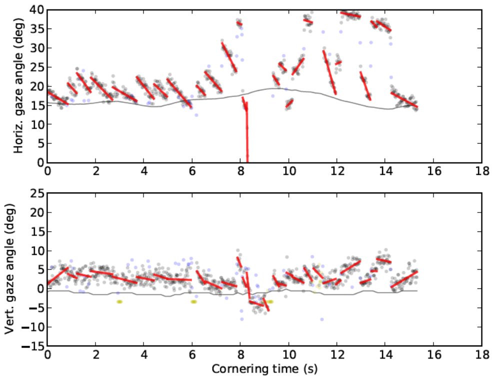

Subject 16, lap 6, noise std h: 0.970, v: 1.185

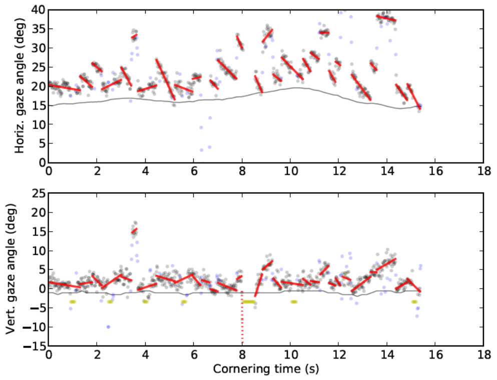

Subject 16, lap 7, noise std h: 1.051, v: 1.125

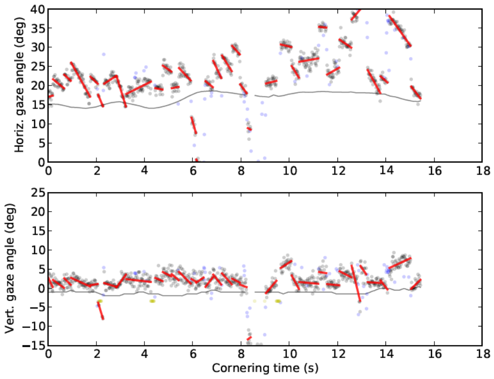

Subject 16, lap 8, noise std h: 1.181, v: 1.401

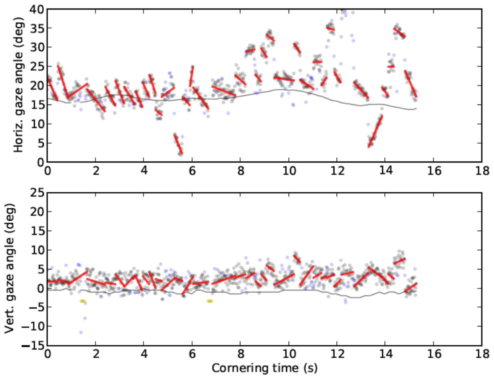

Subject 16, lap 9, noise std h: 1.271, v: 1.372

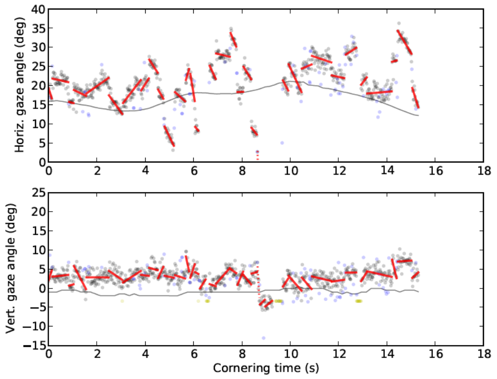

Subject 16, lap 10, noise std h: 1.160, v: 1.182

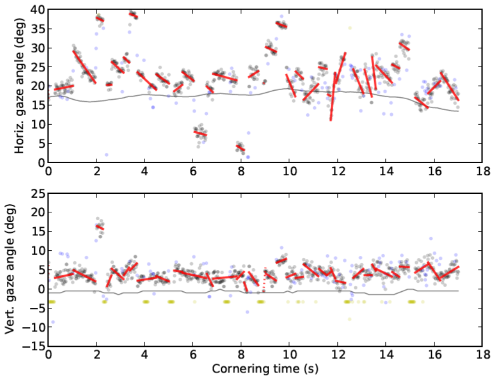

Subject 16, lap 11, noise std h: 1.316, v: 1.296

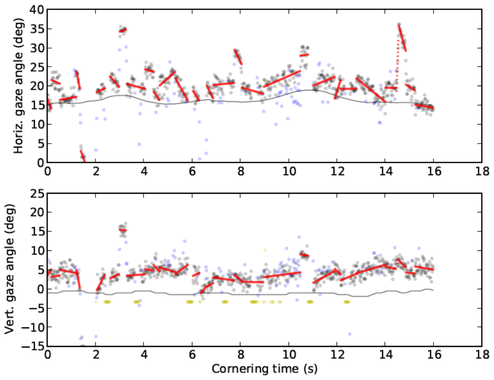

Subject 16, lap 12, noise std h: 1.274, v: 1.332

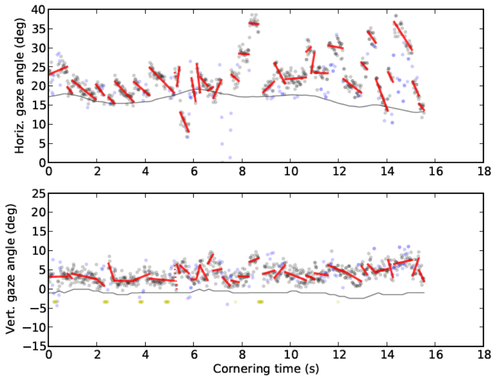

Subject 16, lap 13, noise std h: 1.260, v: 1.301

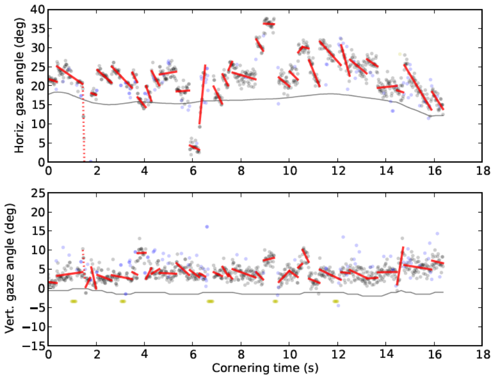

Subject 16, lap 14, noise std h: 1.254, v: 1.347

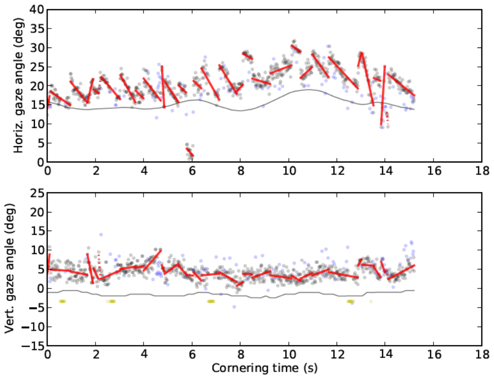

Subject 16, lap 15, noise std h: 1.351, v: 1.284

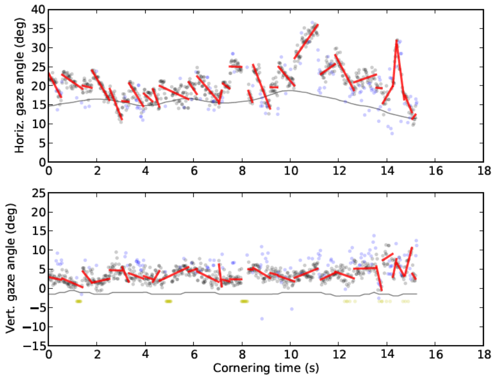

Subject 16, lap 16, noise std h: 1.270, v: 1.232

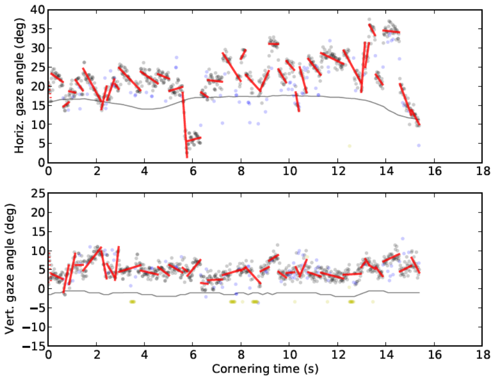

Subject 17, lap 1, noise std h: 0.887, v: 1.193

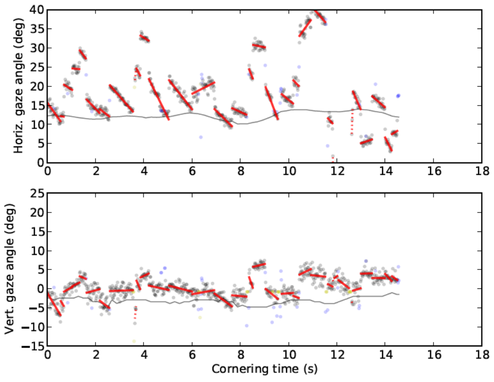

Subject 17, lap 2, noise std h: 0.882, v: 1.076

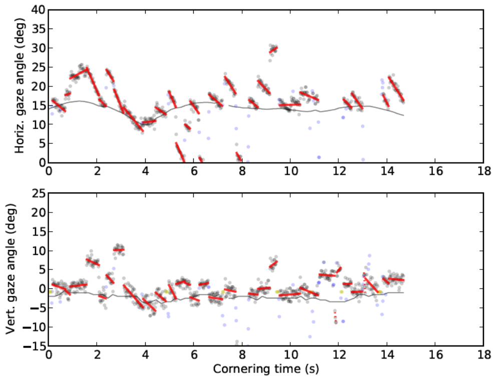

Subject 17, lap 3, noise std h: 0.848, v: 1.131

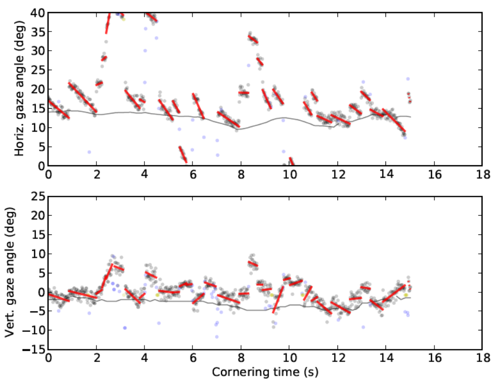

Subject 17, lap 4, noise std h: 0.902, v: 1.107

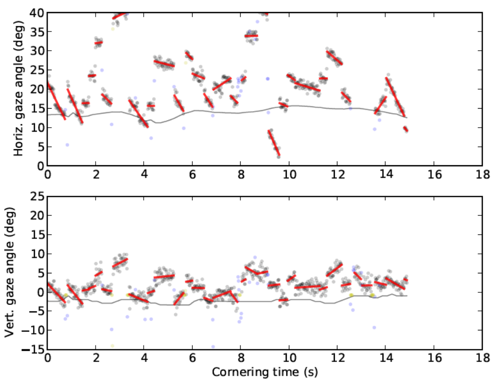

Subject 17, lap 5, noise std h: 0.873, v: 1.066

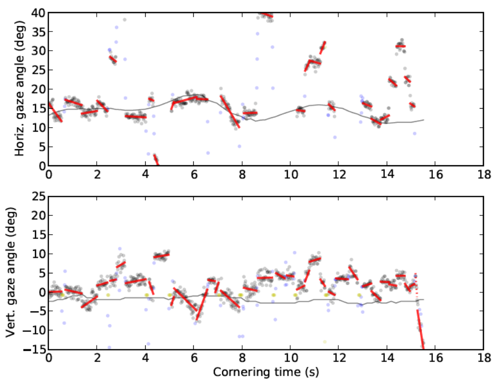

Subject 17, lap 6, noise std h: 0.883, v: 1.261

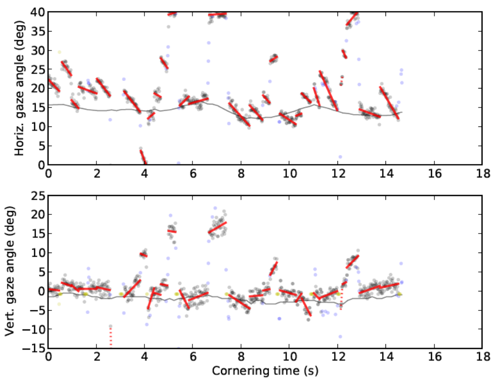

Subject 17, lap 7, noise std h: 0.855, v: 1.165

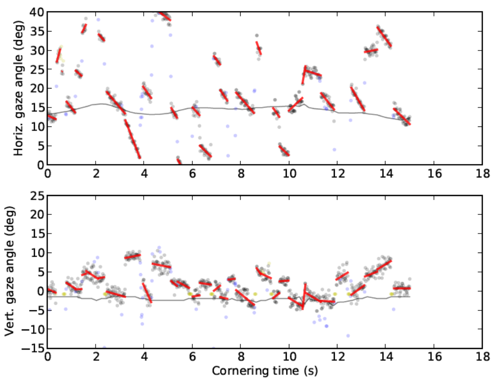

Subject 17, lap 8, noise std h: 0.862, v: 1.063

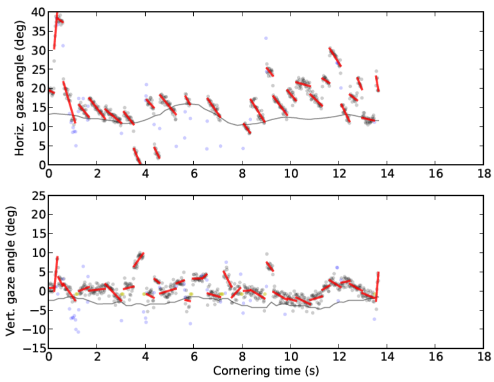

Subject 17, lap 9, noise std h: 0.943, v: 1.203

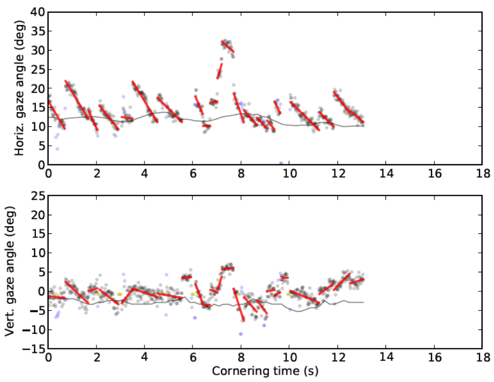

Subject 17, lap 10, noise std h: 0.891, v: 1.102

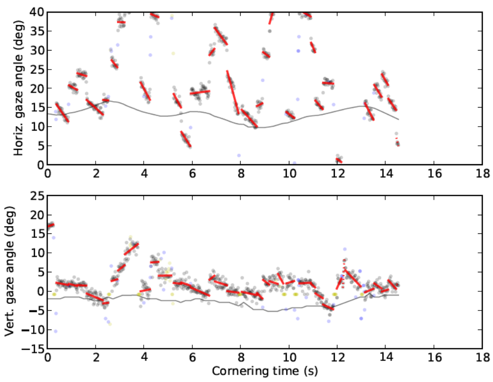

Subject 17, lap 11, noise std h: 0.881, v: 1.120

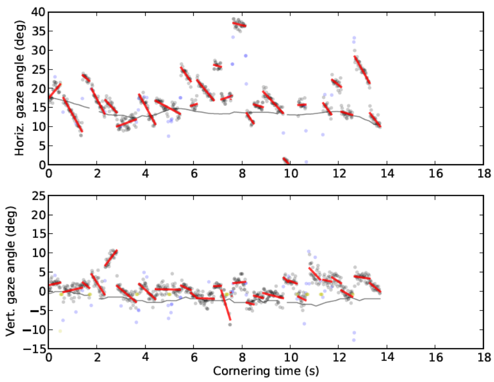

Subject 18, lap 1, noise std h: 0.763, v: 0.793

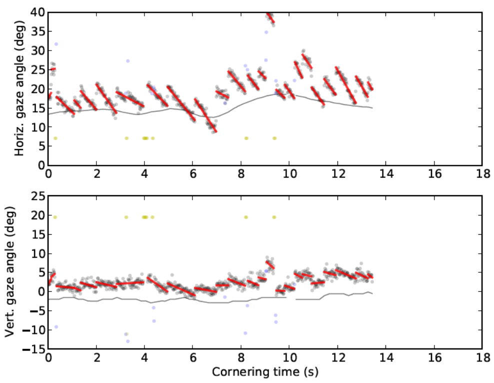

Subject 18, lap 2, noise std h: 0.787, v: 0.729

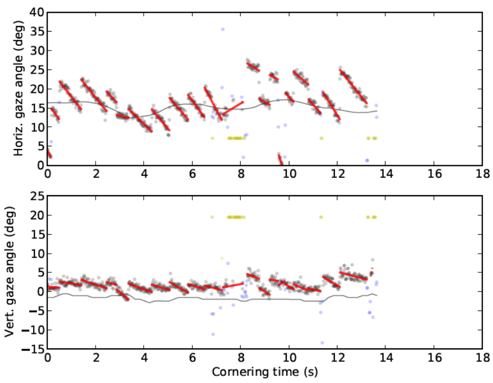

Subject 18, lap 3, noise std h: 0.794, v: 0.779

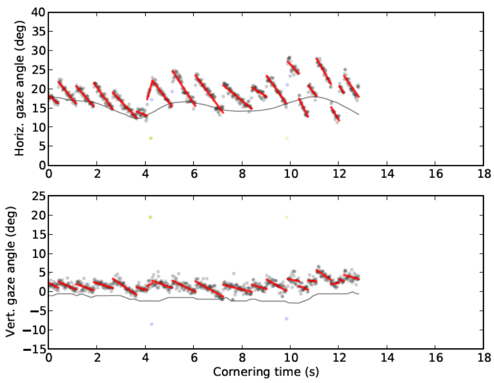

Subject 18, lap 4, noise std h: 0.795, v: 0.825

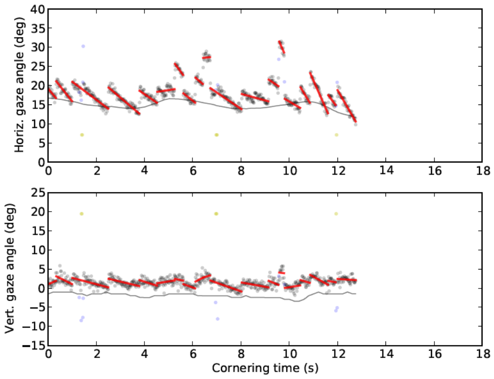

Subject 18, lap 5, noise std h: 0.727, v: 0.795

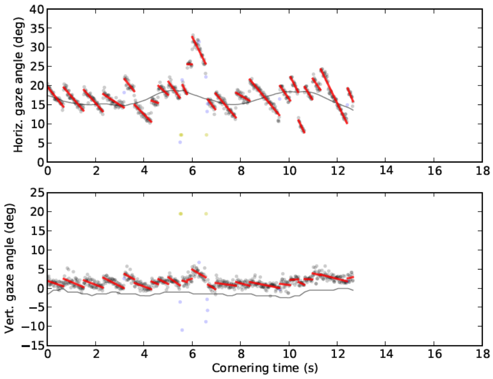

Subject 18, lap 6, noise std h: 0.851, v: 0.828

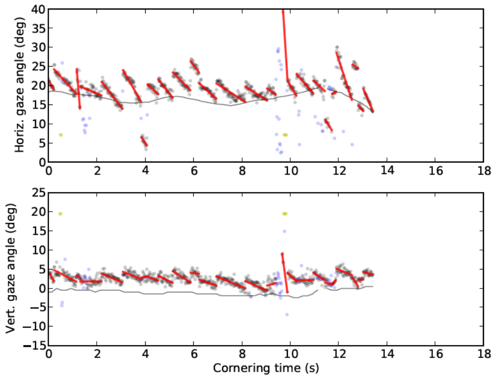

Subject 18, lap 7, noise std h: 0.882, v: 0.835

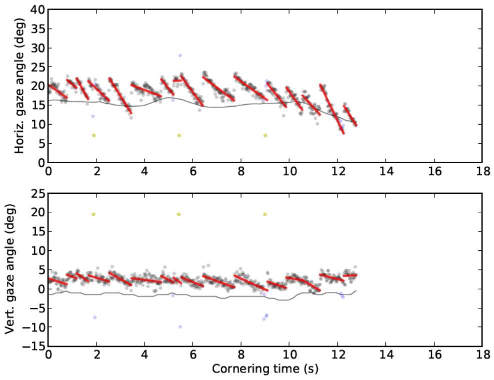

Subject 18, lap 8, noise std h: 0.823, v: 0.746

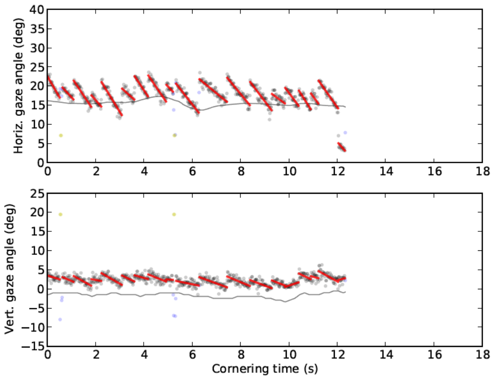

Subject 18, lap 9, noise std h: 0.826, v: 0.791

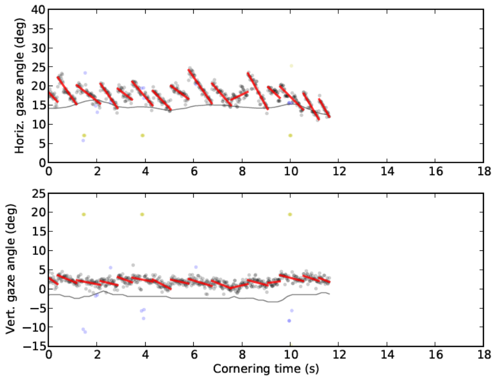

Subject 18, lap 10, noise std h: 0.856, v: 0.889

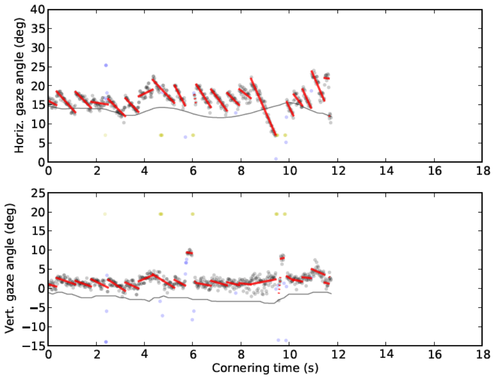

Subject 18, lap 11, noise std h: 0.813, v: 0.718

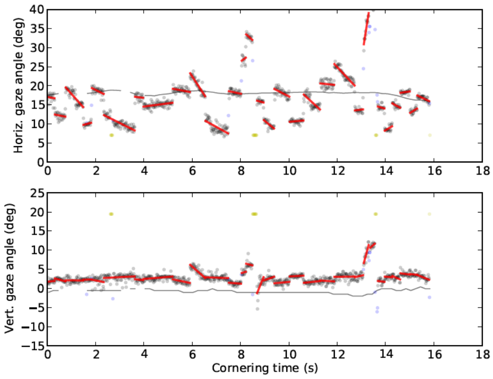

Subject 18, lap 12, noise std h: 0.872, v: 0.717

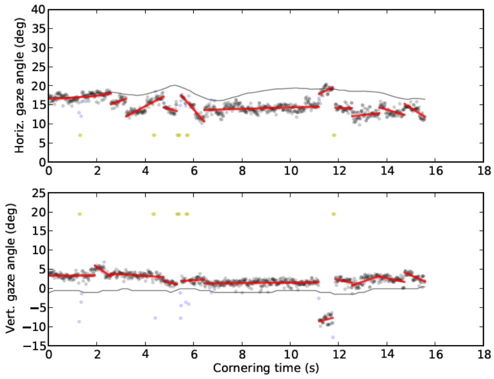

Subject 18, lap 13, noise std h: 0.779, v: 0.822

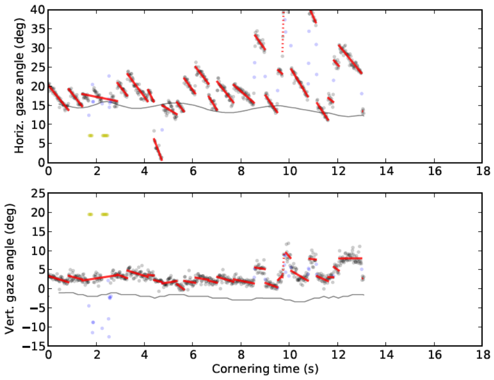

Subject 18, lap 14, noise std h: 0.778, v: 0.861

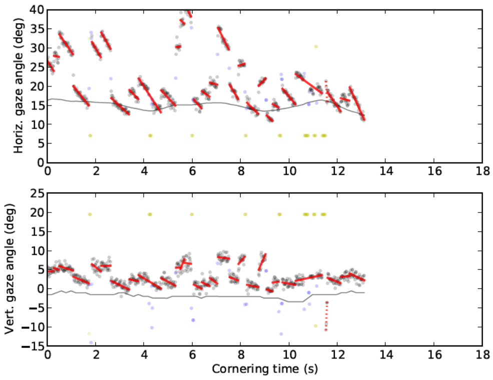

Subject 18, lap 15, noise std h: 0.793, v: 0.693

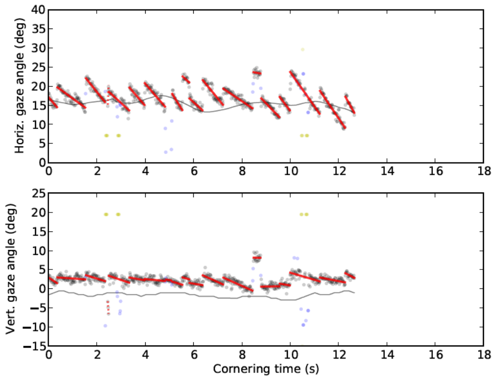

Subject 18, lap 16, noise std h: 0.809, v: 0.790

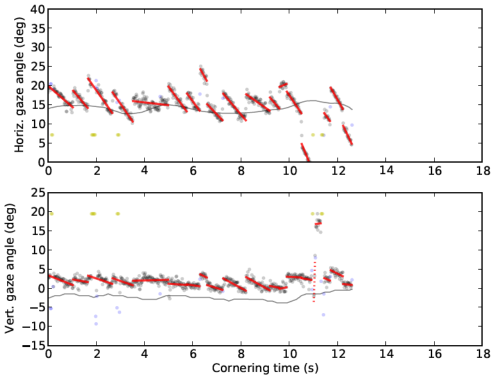

Subject 19, lap 1, noise std h: 0.810, v: 0.739

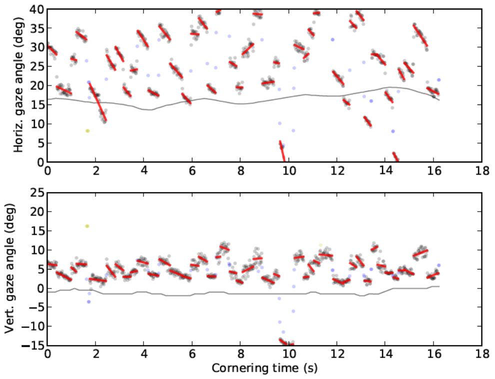

Subject 19, lap 2, noise std h: 0.759, v: 0.717

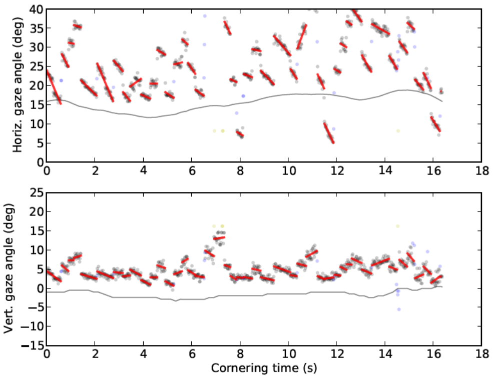

Subject 19, lap 3, noise std h: 0.812, v: 0.944

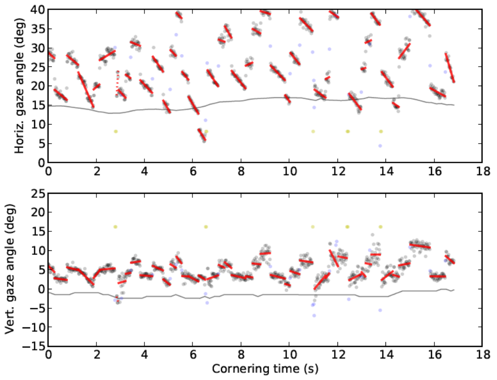

Subject 19, lap 4, noise std h: 0.822, v: 0.919

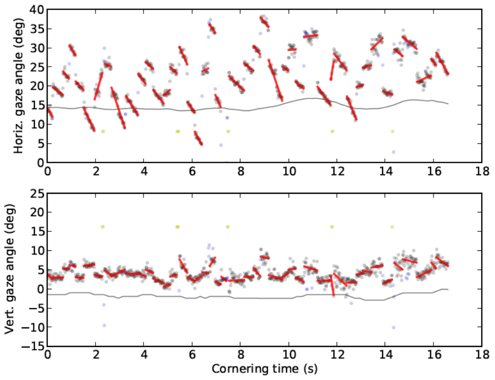

Subject 19, lap 5, noise std h: 0.902, v: 0.939

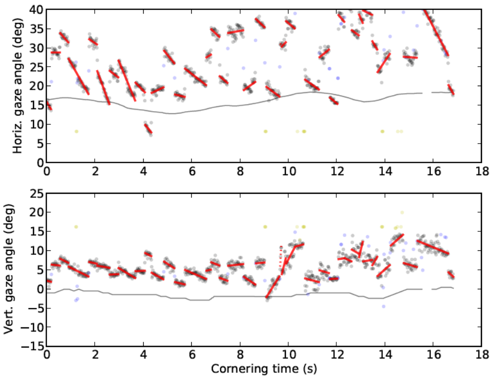

Subject 19, lap 6, noise std h: 0.948, v: 1.235

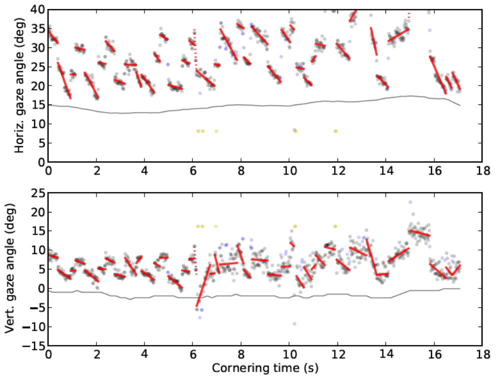

Subject 19, lap 7, noise std h: 0.872, v: 0.993

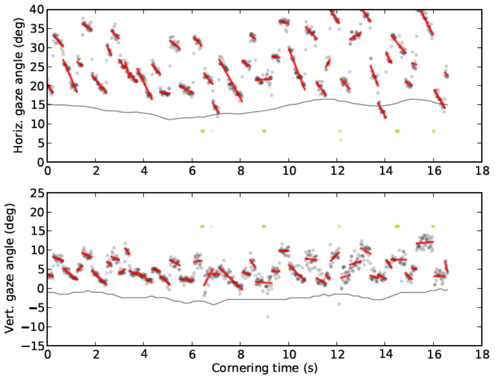

Subject 19, lap 8, noise std h: 0.865, v: 1.107

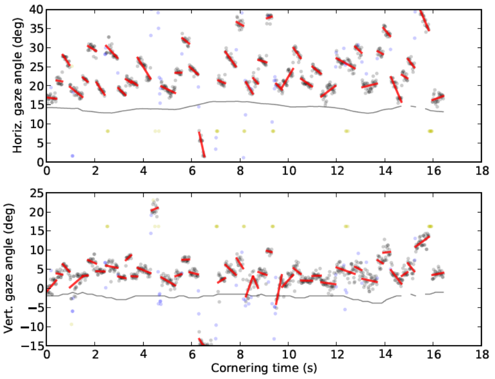

Subject 19, lap 9, noise std h: 0.914, v: 1.090

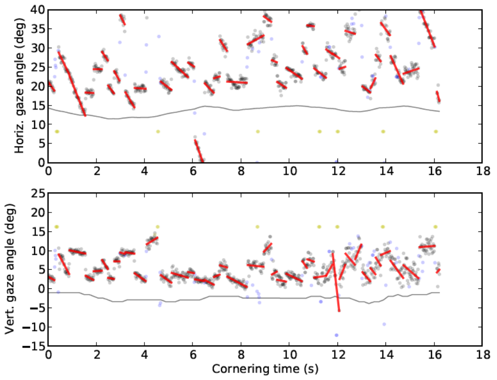

Subject 19, lap 10, noise std h: 0.821, v: 1.155

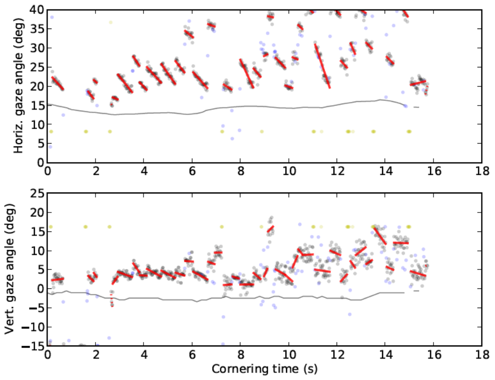

Subject 19, lap 11, noise std h: 0.841, v: 1.251

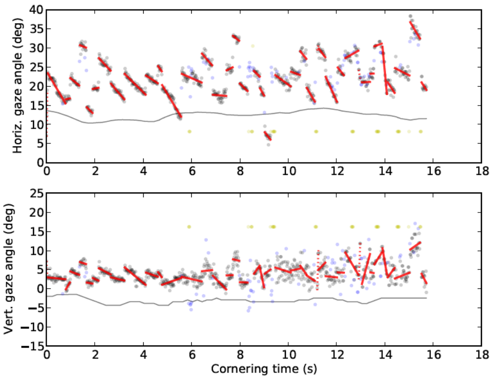

Subject 19, lap 12, noise std h: 0.947, v: 1.049

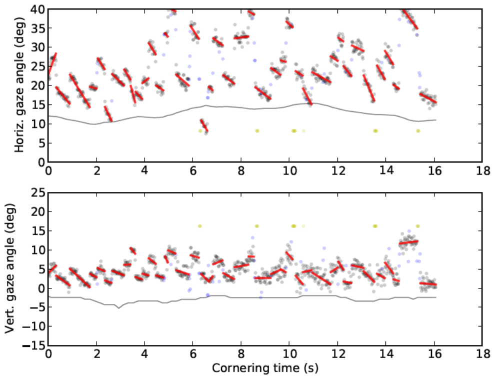

Subject 19, lap 13, noise std h: 0.799, v: 0.931

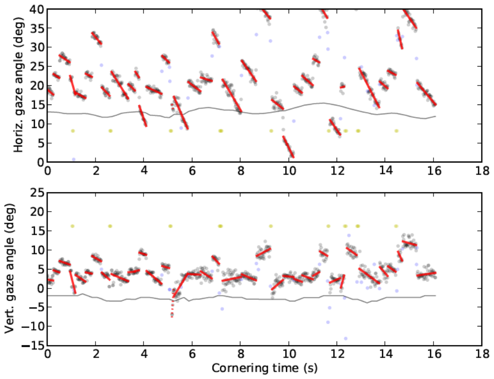

Subject 19, lap 14, noise std h: 0.881, v: 0.864

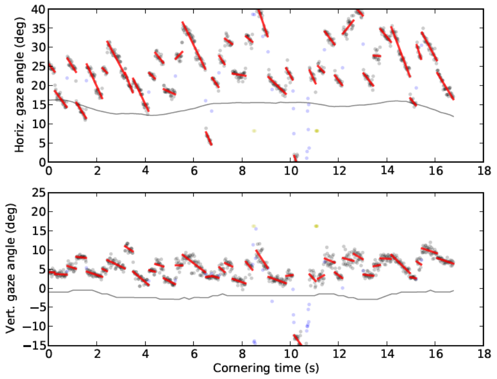

Subject 19, lap 15, noise std h: 0.769, v: 0.804

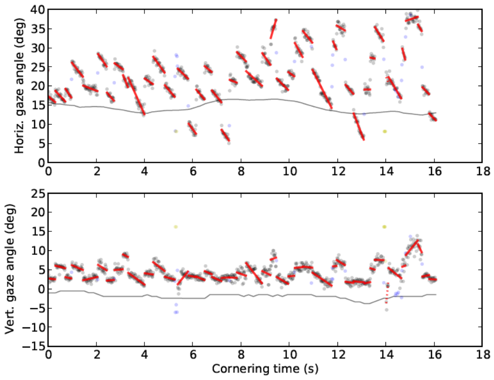

Subject 19, lap 16, noise std h: 0.892, v: 0.981

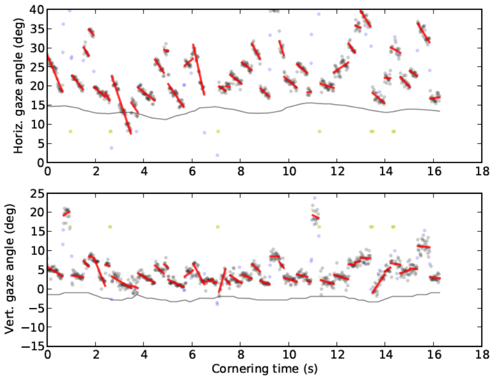

Subject 21, lap 1, noise std h: 1.044, v: 1.163

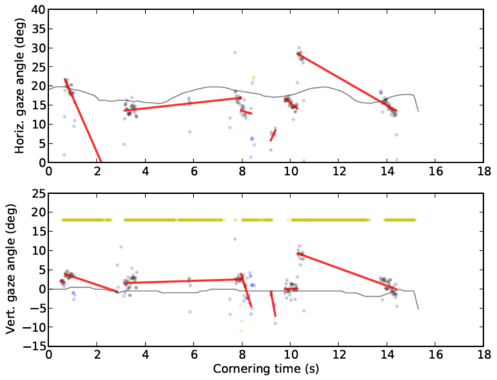

Subject 21, lap 2, noise std h: 0.935, v: 1.131

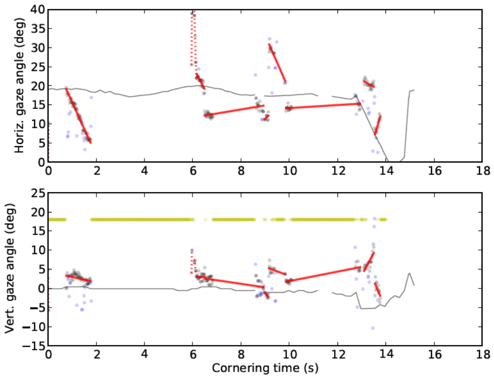

Subject 21, lap 3, noise std h: 1.007, v: 1.058

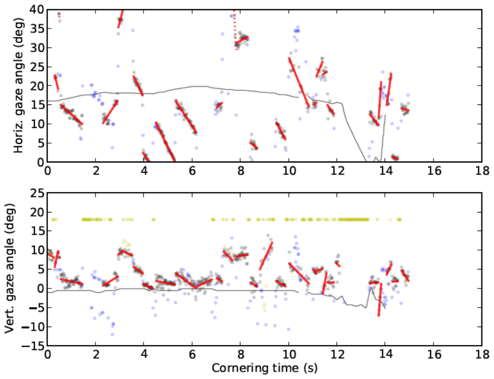

Subject 21, lap 4, noise std h: 0.825, v: 1.021

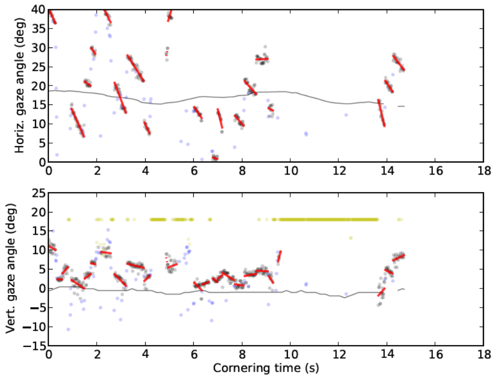

Subject 21, lap 5, noise std h: 1.038, v: 0.993

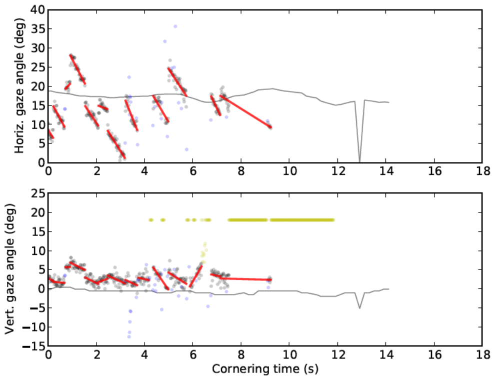

Subject 21, lap 6, noise std h: 1.030, v: 1.164

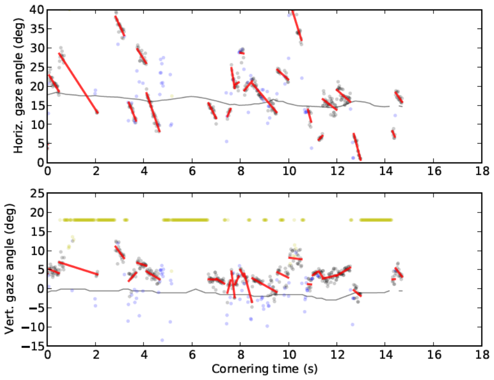

Subject 21, lap 7, noise std h: 1.114, v: 1.139

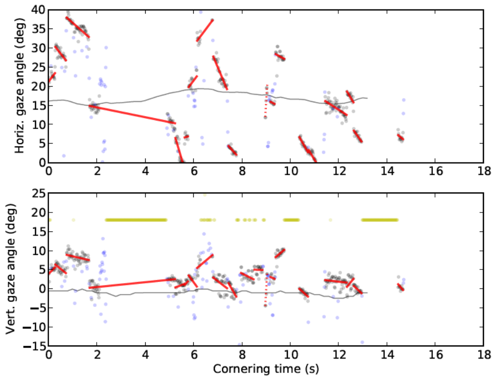

Subject 21, lap 8, noise std h: 0.861, v: 1.406

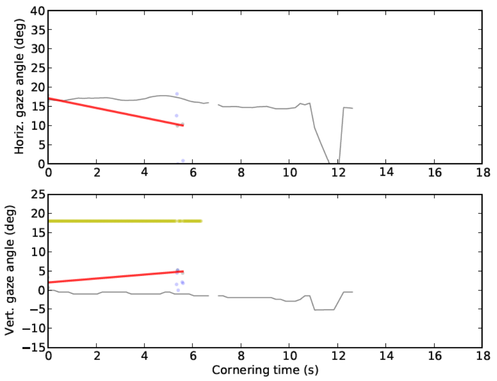

Subject 21, lap 9, noise std h: 1.069, v: 0.945

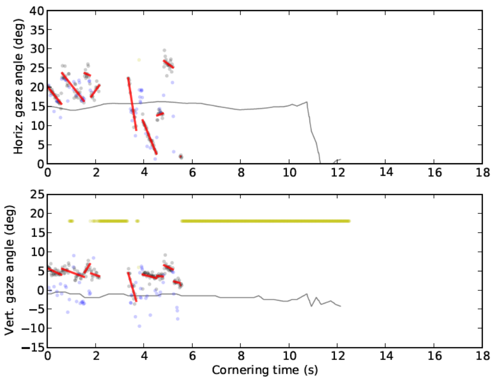

Subject 21, lap 10, noise std h: 1.488, v: 1.468

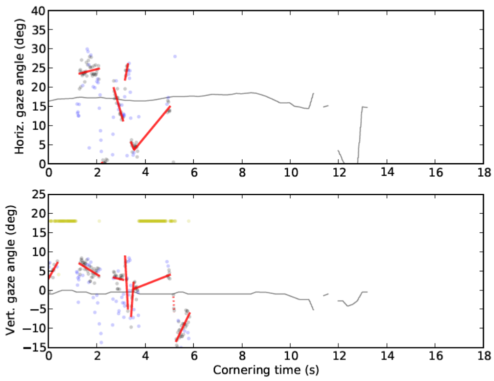

Subject 21, lap 11, noise std h: 0.989, v: 1.135

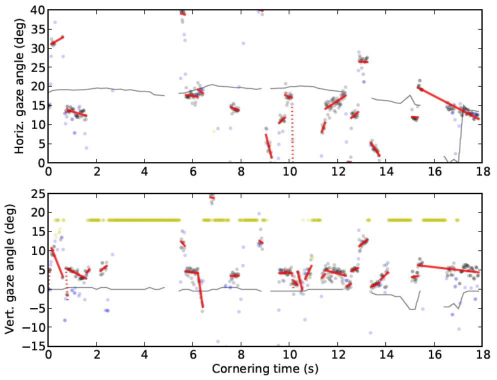

Subject 21, lap 12, noise std h: 0.930, v: 1.154

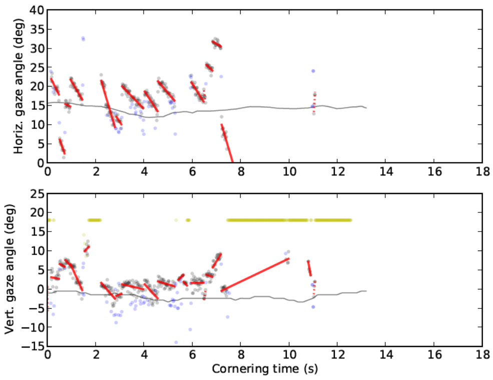

Subject 21, lap 13, noise std h: 1.065, v: 1.056

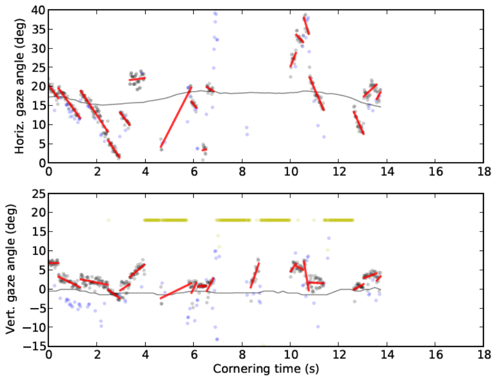

Subject 21, lap 14, noise std h: 1.289, v: 1.491

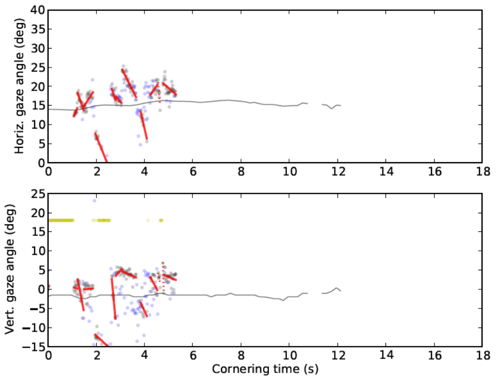

Subject 21, lap 15, noise std h: 0.968, v: 1.164

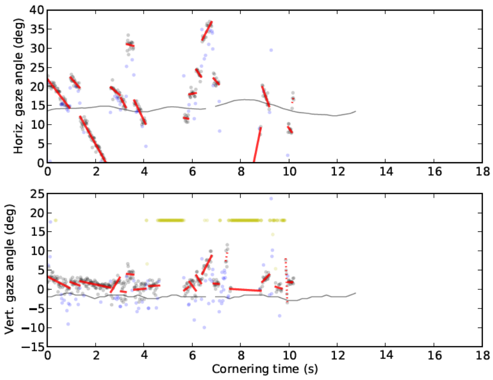

Subject 21, lap 16, noise std h: 0.878, v: 1.280

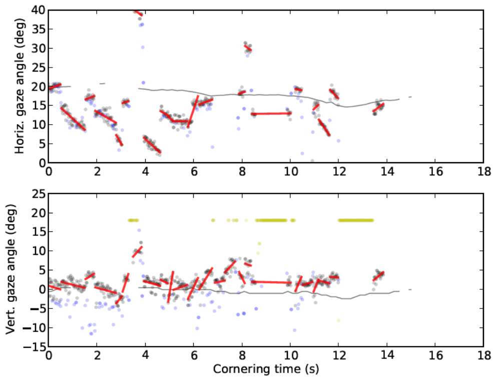

Supplement: Information S1 — (PDF) [file pone.0068326.s001.pdf]
